# Mapping the Apps: Ethical and Legal Issues with Crowdsourced Smartphone Data using mHealth Applications

**DOI:** 10.1007/s41649-024-00296-3

**Published:** 2024-06-18

**Authors:** Nada Farag, Alycia Noë, Dimitri Patrinos, Ma’n H. Zawati

**Affiliations:** Centre of Genomics and Policy, McGill University, Montreal, Canada

**Keywords:** Crowdsourcing, Algorithms, Data collection, Ethics, Law

## Abstract

More than 5 billion people in the world own a smartphone. More than half of these have been used to collect and process health-related data. As such, the existing volume of potentially exploitable health data is unprecedentedly large and growing rapidly. Mobile health applications (apps) on smartphones are some of the worst offenders and are increasingly being used for gathering and exchanging significant amounts of personal health data from the public. This data is often utilized for health research purposes and for algorithm training. While there are advantages to utilizing this data for expanding health knowledge, there are associated risks for the users of these apps, such as privacy concerns and the protection of their data. Consequently, gaining a deeper comprehension of how apps collect and crowdsource data is crucial. To explore how apps are crowdsourcing data and to identify potential ethical, legal, and social issues (ELSI), we conducted an examination of the Apple App Store and the Google Play Store in North America and Europe to identify apps that could potentially gather health data through crowdsourcing. Subsequently, we analyzed their privacy policies, terms of use, and other related documentation to gain insights into the utilization of users’ data and the possibility of repurposing it for research or algorithm training purposes. More specifically, we reviewed privacy policies to identify clauses pertaining to the following key categories: research, data sharing, privacy/confidentiality, commercialization, and return of findings. Based on the results of these app search, we developed an App Atlas that presents apps which crowdsource data for research or algorithm training. We identified 46 apps available in the European and Canadian markets that either openly crowdsource health data for research or algorithm training or retain the legal or technical capability to do so. This app search showed an overall lack of consistency and transparency in privacy policies that poses challenges to user comprehensibility, trust, and informed consent. A significant proportion of applications presented contradictions or exhibited considerable ambiguity. For instance, the vast majority of privacy policies in the App Atlas contain ambiguous or contradictory language regarding the sharing of users’ data with third parties. This raises a number of ethico-legal concerns which will require further academic and policy attention to ensure a balance between protecting individual interests and maximizing the scientific utility of crowdsourced data. This article represents a key first step in better understanding these concerns and bringing attention to this important issue.

**Supplementary Information:**

The online version contains supplementary material available at 10.1007/s41649-024-00296-3.

## Introduction

As of 2020, the IQVIA reports that there are over 350,000 smartphone mobile health (mHealth) applications (apps) available for download on the Apple or Google Play (Android) stores (IQVIA 2021). This number has grown ten-fold since 2014 (35,000 identified mHealth apps in 2014; Sunyaev et al. 2015). Smartphone users are increasingly using mHealth apps for various purposes, including general health and wellness, illness prevention and management, and tracking health measures (Baxter et al. 2020). mHealth apps have also been proposed as promising strategies to aid in the care of patients with chronic health conditions (Langford et al. 2019). To do so, however, these apps collect and process an unfathomable amount of data from users, including biometric data, health-related symptoms, and genetic data (Bohr and Memarzadeh 2020).

The widespread use of mHealth apps has created new opportunities for crowdsourcing health data, allowing researchers to leverage this data to expand biomedical knowledge. For example, data collected through mHealth apps can transform what used to be “case-based studies to large-scale, data-driven research” (Luo et al. 2016). This was observed in 2020 when 23andMe conducted a study of on COVID-19 that found a correlation between blood type and COVID severity (Shelton et al. 2021). To obtain these results, they studied over one million participants (1,051,032), an audience that would not have been accessible without the use of the data collected on their mHealth application (Shelton et al. 2021).

This example is illustrative of the fact that data collection on these applications is a double-edged sword. While this data now makes large-scale research possible, it raises a number of concerns, such as privacy, transparency, and data protection, especially considering that much of the data collected is shared with third parties (Lang et al. 2020). One study by Tangari et al. (2021) found “serious problems with privacy and inconsistent privacy practices” in the surveyed apps, including lack of a privacy policy, non-compliance with privacy policies, and insecure transmission of user data. Given that many apps collect and process sensitive information, including genetic data, data usage is a key concern for users. This concern is particularly salient given that many app users do not read or understand privacy policies before using the application (Obar and Oeldorf-Hirsch 2020).

These issues are amplified by the use of mHealth data for algorithm training and by the fact that, in many jurisdictions, mHealth apps are not regulated, situating them in a “poorly understood legal space” (Lang and Zawati 2018). Due to the legal and ethical obligations that researchers have when using human data, these ambiguities are likely to deter researchers from using data collected by mHealth apps, hindering their use as a potential source of data for health research and ultimately impeding the benefits that may come from large datasets. Furthermore, with more than half of all smartphone users collecting some form of health data on their devices, it is necessary to understand the implications of widespread data collection and sharing for users and researchers, and how these risks can be mitigated through effective policymaking and governance.

To facilitate the use of smartphone app crowdsourcing in research and to better protect users’ data, it is therefore necessary to better understand the ethical, legal, and social issues (ELSI) associated with these applications (Lang et al. 2023). To do so, we first identified mHealth applications that are presently being used in the EU and Canada, the authors’ respective jurisdictions, for research and algorithm training. Then, the study aimed to identify ELSI issues currently found in mHealth apps on the market.

Accordingly, in this article, we present an “App Atlas,” which documents the landscape of mobile health apps used for health research or algorithm training. The App Atlas is comprised of seven tables (Tables 14–20) that explicitly outline the approaches and the issues raised by these applications’ policies, which are further analyzed by this paper. In addition to providing a snapshot of apps likely engaging in data crowdsourcing, this atlas also highlights the ELSI issues associated with this current landscape, in particular issues of transparency, privacy, confidentiality, and data sharing. This atlas and the analysis of ELSI issues will serve as a useful tool for policymakers for the development of policy and guidance surrounding data crowdsourcing using mHealth apps. Indeed, the ELSI issues identified in this article will require further attention to ensure that users are well informed and that their privacy is adequately protected.

## Methodology

### Search Criteria

Between August 2022 and February 2023, we identified smartphone apps which crowdsource data for research or algorithm training in both the Apple App Store (iOS) and Google Play Store (Android). A series of individual searches were conducted simultaneously in Canada and the EU. The following keywords were used when searching: “research project,” “research participant,” “research study,” “clinical trial,” “citizen science health,” “crowdsourcing health,” “contribute health,” “genetic,” “genetic health,” and “DNA health.”

The search terms were selected after an initial pilot search using terms which we felt would be relevant to the topic of crowdsourcing data for health research and algorithm development. After the initial search, we adjusted these terms due to limitations in the functionality of the Apple App Store and Google Play Store. First, these stores both rely on Natural Language Processing rather than the Boolean logic relied on by most legal research databases. Accordingly, we assumed shorter more complete phrases would generate more relevant results than stringing together long specific search terms. Second, the app marketplaces are set up to allow developers to deliberately try and connect to potential customers or audiences. We thus attempted to craft keywords that would likely be mentioned in the description or title. For instance, for more generic terms, we added the term “health” to the search as including this term ensured that results would include health-related apps. For terms more likely to bring health or genetic related results, we did not include the term “health.”

The searches were conducted on the authors’ smartphones. We recorded the first 50 results for both the Apple App Store and the Google Play Store in Canada and the EU. The results of the searches were compiled into Excel spreadsheets and duplicates were removed.

### Inclusion Criteria

To be selected for the App Atlas, the inclusion criteria were: (1) the app needed to be designed to collect or share health or genomic data for research purposes or delivered algorithmic recommendations based on a user submitted health or genomic data (app function), (2) the app developer discloses that they use data for research purposes or for algorithmic product or service development (developer disclosures), and (3) the developer currently uses the app data for research or algorithmic purposes or has publics research that uses app data (developer activities). Therefore, apps were excluded if their privacy documents were unable to be located and if they were not currently being used for these activities. Apps were also excluded if they were not available in English, were not free to download, access, or use, and if they were intended for use in clinical settings or for use to support clinical studies conducted primarily in a clinical setting.

### App Screening Process

To screen for apps that are likely to engage in crowdsourcing for research and algorithm training purposes, we looked primarily at app title, app description, and other app marketplace disclosures. We screened and removed apps that did not collect health-related data, as well as those that were no longer active, contained dead links, or that were only available to certain citizens.

### App Selection Process

After the initial screening, we followed a set of predetermined selection criteria to systematically identify, screen, and choose smartphone apps, similar to the way that the PRISMA guidelines (Moher et al. 2010) are used to find articles for Systematic Reviews and Meta-Analyses.

We subsequently assessed the eligibility of the apps based on the inclusion and exclusion criteria by reviewing webpages, privacy policies, terms of use, and other publicly available corporate communications to confirm the extent to which reviewed applications use users’ data for research purposes or to train system algorithms.

We began by classifying the information contained in the privacy policies into five categories: research, data sharing, privacy/confidentiality, commercialization, and return of findings. This exercise relied on a classification grid consistent with previous scholarship on mHealth research (Lang et al. 2020). We then identified specific clauses on safeguards for shared data, ambiguous language in the privacy policies, whether the privacy policies addressed genetic data and artificial intelligence, and their research consent provisions. To ensure a comprehensive understanding of the privacy landscape, we expanded our search to include the information contained in the app descriptions, websites, and other external documents. This information was condensed into a table, referred to as the “App Atlas.”

### Content Analysis

Privacy policies and documentation from the final selection of apps were systematically assessed to identify ELSI issues raised by the use of participant data for research or algorithm training. The information concerning these issues was entered into the App Atlas, with the goal of highlighting ambiguities and concerns in these policies in an organized fashion. Within the App Atlas, the following information is outlined, as observed within privacy policies and app websites:

1. Safeguards for data sharing.
2. Ambiguous language surrounding data sharing.
3. Explicit consent for research (opt-in).
4. Consent to research (opt-out).
5. Whether genetic data is used.
6. Whether data may be used in research.
7. Safeguards for privacy/confidentiality.
8. Whether AI/Algorithm is mentioned in website.
9. Whether AI/Algorithm is mentioned in the privacy policy.
10. Research mentions in website and privacy policies.
11. Data-sharing policies across website and privacy policies.
12. Privacy and confidentiality practices across website and privacy policies.
13. Commercialization practices across website and privacy policies.
14. Return of findings practices across website and privacy policies.

## Results

The App Atlas was formulated with the goal of making information regarding ELSI issue of mHealth apps more readily accessible and comparable. It allows mHealth app users to become more conscientious readers of privacy policies and documentation and provides a useful starting point for policymakers to update current regulations and guidelines to better take into account crowdsourced data and mHealth apps.

The primary challenge encountered in the process of data compilation for the App Atlas was the varied format and content of privacy policies. This made information pertaining to ELSI issues much more difficult to locate for our research team. Furthermore, when we expanded our search to include websites and app descriptions, in addition to privacy policies, there was a wide variety of pertinent information located in several difficult to find locations. This was a hurdle not only for the App Atlas, but also represents an everyday challenge to users who are attempting to educate themselves of what data the app uses and how that data is stored and shared.

Of the 50 applications originally shortlisted for this project, 46 had privacy policies that were available and in English (Table 1). These privacy policies were reviewed with five focuses: research/algorithm training, data sharing, privacy/confidentiality, commercialization, and return of findings. These themes were chosen as they arose frequently in the app review process, and have not previously been collectively examined in the literature.

**Table 1:** Applications analyzed in the App Atlas

1. 23andMe - DNA Testing	
2. Ada - Check your Health	
3. Ancestry: Family History & DNA	
4. Apple Research	
5. CovidWatcher	
6. DNA ID, Inc.	
7. DnaNudge	
8. FLARe Research	
9. Gene Doe	
10. GenePlanet	
11. Mass Science	
12. My Toolbox Genomics	
13. MyGeneRank	
14. OH Data Port	
15. Pattern Health	
16. Project Serotonin	
17. StuffThatWorks	
18. Urban Mind	
19. Withings Health mate	
20. ActiveDay - Activity Study	
21. ADHD - Cognitive Research	
22. Andaman7 Private Health Record	
23. Atlas Health	
24. Behavidence Research App	
25. Better- Rewards for Health	
26. Chemo Brain Cognitive Research	
27. Depression Cognitive Research	
28. DNA Fit	
29. Dyscalculia Cognitive Research	
30. Dyslexia Cognitive Research	
31. Fibromyalgia – Research	
32. Google Fit	
33. Happiness Project- Play Games for Science	
34. Healthy Minds Program	
35. Hevy Gym Log Workout	
36. Huawei Health	
37. InsideTracker	
38. Insomnia - Cognitive Research	
39. Medisafe Pill & Med Reminder	
40. MyTherapy Pill Reminder	
41. NeuroPsy Research	
42. Parkinson's Cognitive Research	
43. Renpho Health	
44. Smart Omix by Sharecare	
45. Symptom & Mood Tracker	
46. Symptomate - Symptom checker	

### Research/Algorithm Training Purpose

Although all applications collect health data, only 36/46 privacy policies stated that information collected through the app may be used in research (Tables 2 and Appendix 1). The phrasing of disclosures of the possible use of data in research was often simple, such as “data may be used […] for research and development purposes, as well as scientific publications” (Symptomate-Symptom checker). However, occasionally, these statements were convoluted and hidden in sections of the privacy policy, such as the service provider section of the Parkinson’s Cognitive Research app that states: “partners provide us with services globally, including […] research, and surveys. They will have access to your information as reasonably necessary to perform these tasks on our behalf.”

**Table 2:** mHealth applications that may be used in research

1. 23andMe - DNA Testing	
2. Ada - Check your Health	
3. Ancestry: Family History & DNA	
4. Apple Research	
5. CovidWatcher	
6. DNA ID, Inc.	
7. DnaNudge	
8. GenePlanet	
9. Mass Science	
10. My Toolbox Genomics	
11. MyGeneRank	
12. OH Data Port	
13. Pattern Health	
14. Project Serotonin	
15. StuffThatWorks	
16. Urban Mind	
17. Withings Health mate	
18. ADHD Cognitive Research	
19. Behavidence Research App	
20. Better- Rewards for Health	
21. Chemo Brain Cognitive Research	
22. Depression Cognitive Research	
23. DNA Fit	
24. Dyscalculia Cognitive Research	
25. Dyslexia Cognitive Research	
26. Fibromyalgia - Research	
27. Google Fit	
28. Happiness Project- Play Games for Science	
29. Healthy Minds Program	
30. InsideTracker	
31. Insomnia - Cognitive Research	
32. Medisafe Pill & Med Reminder	
33. NeuroPsy Research	
34. Parkinson's Cognitive Research	
35. Renpho Health	
36. Symptomate - Symptom Checker	

One of the most significant distinctions in research approaches was observed between apps designed to facilitate a specific research project, those used for mass data collection, and those designed for commercial purposes but repurposed for research. Of the applications studied, 23 could be classified as commercial applications, 14 were designed to facilitate a specific research project, and 10 were data collection apps used to recruit participants for mass research (Tables 3 and Appendix 2).

**Table 3:** Categorization of applications in the App Atlas

Commercial	Research project	Mass research
1. 23andMe – DNA Testing	1. CovidWatcher	1. Ada- Check your Health
2. Ancestry: Family History & DNA	2. Expanseeker	2. Apple Research
3. DnaNudge	3. UrbanMind	3. DNA ID, Inc.
4. GenePlanet	4. Active Day	4. FLARe Research
5. My Toolbox Genomics	5. ADHD – Cognitive Research	5. GeneDoe
6. MyGeneRank	6. Better – Rewards for Health	6. Mass Sciences
7. Pattern Health	7. Chemo Brain –Cognitive Research	7. OH Data Port
8. Project Serotonin	8. Depression – Cognitive Research	8. Andaman7 Private Health Record
9. StuffThatWorks	9. Dyscalculia – Cognitive Research	9. NeuroPsy Research
10. Withings Health Mate	10. Dyslexia – Cognitive Research	10. Smart Omix by Sharecare
11. Altas Health	11. Fibromyalgia – Cognitive Research	
12. Behavidence Research App	12. Happiness Project – Play Games for Science	
13. DNA Fit	13. Insomnia – Cognitive Research	
14. Google Fit	14. Parkinson’s – Cognitive Research	
15. Healthy Minds Program		
16. Hevy Gym Log Workout		
17. Huawei Health		
18. InsiderTracker		
19. Medisafe Pill & Med Reminder		
20. MyTherapy Pill Reminder		
21. Renpho Health		
22. Symptom & Mood Tracker		
23. Symptomate – Symptom Checker		

Applications designed to facilitate a specific research project often described their research purpose within their privacy policies, with a clear outline of what was being consented to, whereas commercial applications tended to have “umbrella” provisions relating to consent, using broader statements. For example, CovidWatcher, Columbia University’s COVID-19 study app explicitly states: “CovidWatcher is a research study at Columbia University to understand the development of the coronavirus COVID-19 pandemic, its symptoms, its impact on individuals’ attitudes, behaviors, and daily lives”. On the other hand, GenePlanet, a commercial application, merely states: “GenePlanet can process Personal Data for research purposes to gain new potential insights/findings in science”. From these examples, it is easily observed that applications with specific research projects use much more precise language in their privacy policies than commercial applications. Furthermore, unlike apps designed for mass research and for commercial uses, apps designed for specific research projects were more likely to have an informed consent form, although these forms were only accessible in 8/46 applications (Table 4).

**Table 4:** Applications with accessible informed consent forms

1. Ancestry: Family History & DNA	
2. Apple Research	
3. CovidWatcher	
4. MyToolbox Genomics	
5. MyGeneRank	
6. Behavidence	
7. Better- Rewards for Health	
8. GoogleFit	

Interestingly, apps designed for mass data collection often lacked privacy information for the app user. Rather, their privacy documents addressed the researchers who may use the data gathered by the app, rather than the users, who are poised to become participants of the research. For example, Google Fit provides information to researchers directing them to limit the use of participant data collection and providing instructions for de-identification of data. On the topic of anonymization of data, commercial applications generally reserved the right to use de-identified, anonymized, or aggregate data for research without obtaining consent. For example, InsideTracker reserves the right to “disclose aggregated, or other non-Personal Information or information about […] users without restriction” (Table 5). Keywords that hint towards unconstrained sharing of anonymized data are “without restriction” and “sole discretion” as used by InsideTracker, Medisafe Pill & Med Reminder, and Project Serotonin, among others. This anonymization approach was used in 14/46 applications.

**Table 5:** Use of anonymized data

App name	Use
23andMe	“Your **de-identified genetic information** and/or self-reported information may be used for research.”
Ada- Check your Health	“Please be aware that information of your use of the branded care options might be shared with our partners in aggregated or anonymous form **even when you revoked your consent.**”
Ancestry: Family History and DNA	“Ancestry may disclose user information in an **aggregated form** as part of the Services or our marketing, or in scientific publications published by us or our research partners.”
Apple Research	“Apple **will not have access to any contact information or other identifying data** that you provide through the Research app.”
DNA Nudge	“From time to time, we may use **automatic data collection technologies to collect anonymized product data** for research, development, and statistical purposes.”
Gene Planet	“Your […] Biological Sample and Personal Data will be **stored in pseudonymized** form until you request their destruction. In cases when they are used for research purposed, they will be **anonymised**.”
Mass Science	“Only **pseudo-anonymised** and processed data will be available to our analysts.”
My Toolbox Genomics	“From time to time, we provide a limited set of information to service providers who help us develop and aggregate information on our customers’ […] We and our service providers **take steps to minimize the use of any Personal Information** (if Personal Information is needed at all).”
MyGeneRank	“Your […] Biological Sample and Personal Data will be **stored in pseudonymized** form until you request their destruction. In cases when they are used for research purposed, they will be **anonymised**.”
OH Data Port	“Even if you use a pseudonym, you should **be aware that data could be connected to your identity**.”
Pattern Health	“We **may disclose Anonymized User-Provided and Automatically Collected Information**: -As required by law […] -With trusted service providers [...] -If Pattern Health is involved in a merger, acquisition, or sale […] -To analytics and customer support companies.
Project Serotonin	“We reserve the right to use Anonymous Data and aggregated and other de-identified information for any purpose and **disclose Anonymous Data to third parties at our sole discretion**, including for research purposes.”
StuffThatWorks	“We employ **industry best practices** to keep your information secure and private including […] **de-identification and anonymization of your personal information**.”
Urban Mind	“When you use the Urban Mind app, you provide information about your age, gender, occupation, lifestyle, and well-being. […] **You will not be asked to provide your name, contact details, or any other information which could identify you.**”
Withings Health Mate	RETENTION PERIOD: **Pseudonymized Data** is retained until account removal. 2. **Anonymization of data for research purposes**”
ADHD – Cognitive Research	“We may share non-personal information that is aggregated or de-identified so that it **cannot reasonably be used to identify an individual**.”
Atlas Health	“Your data is **stored in anonymised form on the Atlas servers** in the United Kingdom, meaning that only Atlas software can read this information.”
Behavidence Research App	“The data is **completely anonymized and never stored together with any personal information**. Even Behavidence employees can not access any of your personal information (e.g: Name, Address, etc).”
Google Fit	“Health Research Applications or Web Services must comply with the below requirements. These requirements apply to the participant data, including the raw data obtained from Google Fit, APIs, and **data aggregated, anonymized, or derived from the participant data or the raw data**.”
Happiness Project- Play Games for Science	“Is it anonymous? Yes. The **data collection is anonymou**s and furthermore we won’t use your information at any point to try and identify you. **We don’t ask for your name or for any personal details**.”
Healthy Minds Program	"We **may also share de-identified and aggregated data with third parties** for research, analytics, or other purposes allowed by applicable law.”
Inside Tracker	"We **may disclose aggregated, or other non-Personal Information or information about our users without restriction**.”
Medisafe Pill and Med Reminder	“We may **de-identify and aggregate Personal Information**; aggregated data will not contain any information that could be used to contact or identify you. […] aggregated data that may be disclosed to or utilized by us, our partners and by third parties **without restriction**, on commercial terms that can be determined in our **sole discretion**.”
MyTherapy Pill Reminder	“We work together with reputable research partners […] who support us with scientific analyses of aggregated user data. **Your data privacy is not compromised in this process and data cannot be traced back to any specific user.**”
NeuroPsy Research	“The **fully anonymized data from these studies are made available on the Internet** as open data under the CC-BY-SA license.”
Renpho Health	“We **may use aggregated, deidentified data** from the above sources for statistical analysis, research, commercial, and other purposes.”
Symptom and Mood Tracker	“Bearable **tracks as little personally identifiable data as possible** to protect its users. We purposefully do not ask for information like Name, Age, Sex, and Gender so as to improve user privacy.”

31/46 applications addressed research consent in their privacy policies (Table 6). Consent for research was either made separate from the consent for using the application (e.g., Ancestry: Family History & DNA) or, where the application was solely designed for research, the applications terms doubled as the research consent terms (e.g., Urban Mind). Therefore, by using the application, users accept the terms of the application as the terms of the research.

**Table 6:** mHealth applications that addressed research consent

1. 23andMe - DNA Testing	
2. Ada - Check your Health	
3. Ancestry: Family History & DNA	
4. Apple Research	
5. CovidWatcher	
6. DNA ID, Inc.	
7. GenePlanet	
8. Mass Science	
9. My Toolbox Genomics	
10. MyGeneRank	
11. OH Data Port	
12. StuffThatWorks	
13. ADHD - Cognitive Research	
14. Behavidence Research App	
15. Better- Rewards for Health	
16. Chemo Brain Cognitive Research	
17. Depression Cognitive Research	
18. DNA Fit	
19. Dyscalculia Cognitive Research	
20. Dyslexia Cognitive Research	
21. Fibromyalgia - Research	
22. Google Fit	
23. Healthy Minds Program	
24. Inside Tracker	
25. Insomnia - Cognitive Research	
26. NeuroPsy Research	
27. Parkinson's Cognitive Research	
28. Renpho Health	
29. Urban Mind	
30. Withings Health Mate	
31. Happiness Project- Play Games for Science	

29/46 applications required specific consent to research on an opt-in basis, while 3/46 employed an opt-out approach to consent (Table 7). However, the opt-in/opt-out distinction was typically ambiguous, and the instructions were often either not discussed, unclear, or required users to reach out to a particular person for their request. To illustrate the confusing language used to discuss consent for research, it is instructive to look closer at the privacy documents of the app DNA ID. DNA ID promotes its “double opt-in rule” by clearly “we won’t share your data with anyone you don’t want”; however, reading more of their privacy documentation suggests that their system for data sharing is an opt-out system, rather than an opt-in system, as “your DNA is the baseline for establishing a share.” Furthermore, there are no instructions provided by DNA ID as to how to opt-in or opt-out.

**Table 7:** Opt-in/opt-out consent models

Applications requiring opt-in consent	Applications using an opt-out consent approach
1. 23andMe - DNA Testing	1. Withings Health Mate
2. Ada - Check your Health	2. Happiness Project- Play Games for Science
3. Ancestry: Family History & DNA	
4. Apple Research	
5. CovidWatcher	
6. DNA ID, Inc.	
7. GenePlanet	
8. Mass Science	
9. My Toolbox Genomics	
10. MyGeneRank	
11. OH Data Port	
12. StuffThatWorks	
13. ADHD - Cognitive Research	
14. Behavidence Research App	
15. Better- Rewards for Health	
16. Chemo Brain Cognitive Research	
17. Depression Cognitive Research	
18. DNA Fit	
19. Dyscalculia Cognitive Research	
20. Dyslexia Cognitive Research	
21. Fibromyalgia - Research	
22. Google Fit	
23. Healthy Minds Program	
24. Inside Tracker	
25. Insomnia - Cognitive Research	
26. NeuroPsy Research	
27. Parkinson's Cognitive Research	
28. Renpho Health	
29. Urban Mind	

When expanding the search for consent-related information to beyond privacy policies, 38/46 of applications contained information on research either in their app descriptions available on the Apple Store or Google Play store or on app-affiliated websites. on their website or in the app store descriptions. This leaves 8 applications that use user information for research, but do not mention it on their websites or in app descriptions. Users of these 8 applications would therefore only be unaware of the possible use of their data for research unless they closely read the privacy policy. Moreover, only 8/46 of the websites provide an informed consent form that discussed potential risks of research participation.

Another issue is the ease of finding information regarding these apps use of data in research. Across all websites, app descriptions, and privacy policies, 42/46 apps mention research in one or several locations (Table 8). Yet, there is no uniformity as to where this information can be found. Information on research could be contained in a separate link, within the FAQ section, in the privacy policy, or elsewhere. This makes it extremely difficult for users to easily find information on their privacy concerns, even when making efforts to search for the information. Moreover, for apps designed to facilitate mass data collection, their research information pages were often targeted towards the researchers, with little information for users (e.g., Google Fit).

**Table 8:** Applications with research-related privacy information in multiple locations

1. 23andMe - DNA Testing	
2. Ada - Check your Health	
3. Ancestry: Family History & DNA	
4. Apple Research	
5. CovidWatcher	
6. DNA ID, Inc.	
7. DnaNudge	
8. FLARe Research	
9. GenePlanet	
10. Mass Science	
11. My Toolbox Genomics	
12. MyGeneRank	
13. OH Data Port	
14. Pattern Health	
15. Project Serotonin	
16. StuffThatWorks	
17. Urban Mind	
18. Withings Health mate	
19. ADHD - Cognitive Research	
20. Andaman7 Private Health Record	
21. Atlas Health	
22. Behavidence Research App	
23. Better- Rewards for Health	
24. Chemo Brain Cognitive Research	
25. Depression Cognitive Research	
26. DNA Fit	
27. Dyscalculia Cognitive Research	
28. Dyslexia Cognitive Research	
29. Fibromyalgia - Research	
30. Google Fit	
31. Happiness Project- Play Games for Science	
32. Healthy Minds Program	
33. InsideTracker	
34. Insomnia - Cognitive Research	
35. Medisafe Pill & Med Reminder	
36. MyTherapy Pill Reminder	
37. NeuroPsy Research	
38. Parkinson's Cognitive Research	
39. Renpho Health	
40. Smart Omix by Sharecare	
41. Symptom & Mood Tracker	
42. Symptomate - Symptom checker	

Based on the mHealth applications in the App Atlas, apps can be broadly categorized into 4 subtypes: genetic applications, symptom checker applications, mental health applications, and tracker applications. While there are overlaps between these categories, the categories were chosen based on the types and extent of user information collected. For genetic applications, such as 23andMe, Ancestry, Inside Tracker, and DNA Nudge, algorithms are used to characterize genetic composition, link user DNA with other users in the same family tree, and provide recommendations for food, drink, and skincare based on genetics. Symptom checker applications, such as Ada and StuffThatWorks, utilize a medical AI to link inputted symptoms with diagnoses and treatments. These AIs often incorporate their user data into their algorithm to improve their services over time. This can also serve as a powerful tool for crowdsourcing scientific advancements. Mental health apps, such as Behavidence and Symptomate, use an AI-based algorithm to predict mental wellbeing based on cellphone use and interaction. Finally, tracker apps, such as Renpho Health and Medisafe, help users track and visualize their data, and may also provide recommendations based on this data.

### Data Sharing

In general, applications linked to a particular research project (e.g., CovidWatcher) had the shortest list of entities with which the information would be shared. The standard data-sharing privacy policies state that data would be shared with legal authorities and/or the government under valid subpoena or warrant, and in the case of a merger/acquisition of the business for commercial applications (Appendix 3). Unlike other statements in mHealth app privacy policies, exceptions to the privacy and confidentiality of health data collected are explicitly stated, in clear language. For example, Project Serotonin declares: “We may share some or all of your Personal Data in connection with or during negotiation of a merger.” Furthermore, while 23andMe and Ancestry have been in public discourse for sharing personal data with police for criminal investigations (Kaiser 2019), their privacy documents allow for this behaviour in certain circumstances: “23andMe will not provide information to law enforcement unless required to by law to comply with a valid court order, subpoena, or search warrant.” Although the public is concerned about the data-sharing policies of 23andMe and Ancestry, as these are widely used apps, they were also the only 2 applications that explicitly mentioned never sharing personal information with employers or insurance companies. This means the door is frequently left open for sharing data with organizations that users are unaware of.

Additionally, privacy policies of mHealth apps frequently indicated they would share personal information with their service providers for data processing/storage. This possibility of sharing personal information with third party service providers is often described in a vague manner to leave plenty of leeway for the app to share personal information that the user may not want to be shared. For example, StuffThatWorks states: “We are partnering with a number of selected service providers [… who] may receive or otherwise have access to your Personal Information.” This statement lacks any description of what the service providers are doing beyond services that “facilitate and enhance” the StuffThatWorks app and suggests that the personal data shared with service providers does not need to be de-identified or anonymized as it could limit the enhancement of the app. The only comfort provided for the user is that any data disclosures to service providers are subject to “confidentiality obligations.”

As alluded to above, many applications identified a distinction between sharing personal information and “de-identified” information, with the suggestion personal information being more protected. The sharing of personal information was restricted to service providers or circumstances when users provided explicit consent (even though, it is often unclear as to the procedure used to obtain this consent). De-identified information, however, could often be shared without any further consent beyond the user using the mHealth app itself. For instance, InsiderTracker makes anonymized blood test information and self-reported health information available to third parties for research purposes without consent, but will not share an identifying information without prior consent. These supposed protections were frequently reinforced by legislation such as the Personal Health Information Protection Act (PHIPA), the Health Insurance Portability and Accountability Act (HIPPA), or the General Data Protection Regulation (GDPR). 11/46 apps explicitly mention region-specific privacy legislation, such as GDPR, as a way to further reassure users of their protections (Tables 9 and Appendix 4). Although the terms and conditions in the Canadian and EU stores are the same, a separate section would be added for the European Union or other regions in these applications. These mentions of privacy legislation are often very vague and presented with a tone of “just trust us” because we are, as Andaman7 Private Health Record says “fully GDPR and HIPAA complaint.”

**Table 9:** Applications that mention privacy legislation

1. GenePlanet	
2. Andaman7 Private Health Record	
3. Symptom & Mood Tracker	
4. Symptomate – Symptom Checker	
5. Atlas Health	
6. Pattern Health	
7. Ada – Check your Health	
8. Withings Health Mate	
9. DNA Fit	
10. MyTherapy Pill Reminder	
11. Active Day	

Overall, sections addressing data sharing were unclear. A worrying 40/46 applications contained contradictory or ambiguous language surrounding data sharing. Users have little chance of understanding how their personal data would be treated, assuming users spend time reading the privacy documents. It was hard to differentiate between the when personal data and de-identified data would be shared, whether user consent was required for this sharing, whether that consent was on an opt-in or opt-out basis, and the procedures for how user would go about providing consent for data sharing. After all, only 8/46 applications provided a method of contact (i.e., email address) for the user to send questions pertaining to issues of data sharing, privacy, and confidentiality (Table 10).

**Table 10:** Applications with a clear method of contact

1. DNA ID, Inc.	
2. My GeneRank	
3. StuffThatWorks	
4. Atlas Health	
5. Behavidence Research App	
6. DNA Fit	
7. MyTherapy Pill Reminder	
8. Smart Mix by Sharecare	

### Privacy/Confidentiality

27/46 applications describe safeguards or protocols to protect user information (Tables 11 and Appendix 4). Most applications contained a waiver of liability in case of a data breach. This waiver often included a disclaimer that third party websites and businesses may be embedded into the application, and that these have their own privacy policies that the application cannot be held responsible for. The waivers of liability were often similar. In fact, 8/46 apps contained the same general statement that underscored there is no guarantee of data security for the user: “We implement security safeguards designed to protect your data, such as HTTPS. We regularly monitor our systems for possible vulnerabilities and attacks. However, we cannot warrant the security of any information that you send us. There is no guarantee that data may not be accessed, disclosed, altered, or destroyed by breach of any of our physical, technical, or managerial safeguards.”

**Table 11:** Applications with safeguards to protect user information

App	Safeguard
23andMe – DNA Testing	“We **implement procedures and maintain contractual terms** with each service provider and contractor **to protect the confidentiality and security of your Personal Information**.”
Ada – Check your Health*	“We may share and present the results as summarized statistics to our partners, e.g. in the public health and scientific community, always on an **irreversibly anonymized basis**.”
CovidWatcher	“Your data may be shared as part of research collaboration with other research teams. However, **only the ‘de-identified’ part of your data will be shared**. We will not share any of your data with any non-research third party.”
DnaNudge	“We ensure **confidentiality obligations are put in place when dealing with our Related Parties and other third parties**”
GenePlanet	“**We only work with companies that meet and commit to our safety standards**”
MyGeneRank	“We may **share your coded study data (data without your name, date of birth, or email address) with other researchers** as permitted by the informed consent for MyGeneRank studies. Individual level genetic data collected by the MyGeneRank website and online services will not be shared.”
Pattern Health	“We provide **physical, electronic, and procedural safeguards to protect information we process and maintain**. For example, we limit access to this information to authorized employees and contractors who need to know that information in order to operate, develop or improve our products.”
Project Serotonin	“While we **implement procedures and contractual obligations on our service providers designed to protect the confidentiality and security of your information**, we cannot guarantee the confidentiality and security of your information due to the inherent risks associated with storing and transmitting data electronically.”
StuffThatWorks	“When disclosing information to our ---Partners or otherwise selling user information for scientific or market research purposes, we **make sure to anonymize and/or remove all Personal Information or other personally-identifying indicators in the data** (de-identification) to minimize the possibility of accidental member identification.”
Urban Mind	“The information you provide may also be shared securely with organisations who provide services to us in connection with the research purposes, for example data storage, as stated in this Privacy Policy. **We will not disclose your information to unrelated organisations or third parties under any circumstances**.”
Withings Health mate	“**Our subcontractors are required to comply with both the GDPR**.”
ADHD – Cognitive Research	“We transfer information to our corporate affiliates, service providers, and other partners who process it for us, based on our instructions, and in compliance with this policy and any other appropriate confidentiality and security measures. **They will have access to your information as reasonably necessary to perform these tasks on our behalf and are obligated not to disclose or use it for other purposes.**”
Andaman7 Private Health Record	“A7S **contractually ensures that the subcontractor cannot process this data for own purposes** independent of the purposes for which A7S uses the subcontractor.”
Atlas Health	“These third parties have access to your Personal Data **only to perform these tasks on our behalf and are obligated not to disclose or use it for any other purpose.**”
Behavidence Research App	“These companies are authorized to use your statistical information, **which does not contain any identifying details about you, in this context only as necessary to provide these services to us and not for their own promotional purposes.”**
Better- Rewards for Health	“We do not sell any of your Personal Information or Personal Health Data to any third parties. **Your information is encrypted and not shared unless you specify otherwise**.”
Chemo Brain Cognitive Research	“We transfer information to our corporate affiliates, service providers, and other partners who process it for us, **based on our instructions, and in compliance with this policy and any other appropriate confidentiality and security measures**. They will have **access to your information as reasonably necessary to perform these tasks** on our behalf and are obligated not to disclose or use it for other purposes.”
Depression Cognitive Research	“We transfer information to our corporate affiliates, service providers, and other partners who process it for us, **based on our instructions, and in compliance with this policy and any other appropriate confidentiality and security measures**. They will have **access to your information as reasonably necessary to perform these tasks** on our behalf and are obligated not to disclose or use it for other purposes.”
DNA Fit	“Research contractors will be screened and will be subject to the rules established by Prenetics, any Information sharing agreements that we may implement, this Privacy Policy and the Consent Document. Any Processors or other third-party service providers will be **required to contractually comply with the principles and objectives of any Prenetics policies, including this Privacy Policy, as well as the requirements of the EU GDPR, the UK GDPR and DPA 2018 and other Applicable Law** and will be required to sign a data processing agreement to confirm that Information will not be collected, used, shared, stored or otherwise for any Purpose other than those instructed by Prenetics EMEA.”
Dyscalculia Cognitive Research	“We transfer information to our corporate affiliates, service providers, and other partners who process it for us, **based on our instructions, and in compliance with this policy and any other appropriate confidentiality and security measures**. They will have **access to your information as reasonably necessary to perform these tasks** on our behalf and are obligated not to disclose or use it for other purposes.”
Dyslexia Cognitive Research	“We transfer information to our corporate affiliates, service providers, and other partners who process it for us, **based on our instructions, and in compliance with this policy and any other appropriate confidentiality and security measures**. They will have **access to your information as reasonably necessary to perform these tasks** on our behalf and are obligated not to disclose or use it for other purposes.”
Fibromyalgia – Research	“We transfer information to our corporate affiliates, service providers, and other partners who process it for us, **based on our instructions, and in compliance with this policy and any other appropriate confidentiality and security measures**. They will have **access to your information as reasonably necessary to perform these tasks** on our behalf and are obligated not to disclose or use it for other purposes.”
Google Fit	“Health Research Applications and Web Services are also recommended to **follow best practices recommended by the U.S. Department of Health and Human Services, the U.S. Food and Drug Administration regulations, or ICH Good Clinical Practice Guidelines**, like applying for a Certificate of Confidentiality from the National Institutes of Health, which may protect data from compelled disclosure to third parties.”
Happiness Project- Play Games for Science	“Is it anonymous? Yes. **The data collection is anonymous and furthermore we won’t use your information at any point to try to identify you**. We don’t ask for your name or for any other personal details, and we don’t need your phone number to send notifications to your phone. We will never sell your data to any third party. We may make anonymous data available for further research by other parties such as academic researchers at other institutions. “
Healthy Minds Program	“We **limit the release of information** to that described in this Privacy Policy, and we **require privacy protections in our business relationships**.”
Insomnia – Cognitive Research	“We transfer information to our corporate affiliates, service providers, and other partners who process it for us, **based on our instructions, and in compliance with this policy and any other appropriate confidentiality and security measures**. They will have **access to your information as reasonably necessary to perform these tasks** on our behalf and are obligated not to disclose or use it for other purposes.”
Parkinson’s Cognitive Research	“We transfer information to our corporate affiliates, service providers, and other partners who process it for us, **based on our instructions, and in compliance with this policy and any other appropriate confidentiality and security measures**. They will have **access to your information as reasonably necessary to perform these tasks** on our behalf and are obligated not to disclose or use it for other purposes.”

Overall, the privacy and confidentiality sections of mHealth app privacy documents were vague and employed ill-defined terms such as “industry standard procedures” (e.g., StuffThatWorks, myToolbox Genomics). There was no uniform format or demarcated section for privacy and confidentiality, rather privacy and confidentiality protocols, if mentioned at all, were dispersed throughout the policy. Of the apps we analyzed, 24/46 app websites mention privacy and confidentiality in some capacity. These were typically framed as either broad assurances that the company takes privacy seriously, or within the FAQ section. 27/46 applications mention anonymization or de-identification as a mechanism to protect user information and 8/46 applications focus on encryption as a reason why users should be reassured about the protection of their personal data. As a general trend, applications that were used for specific research projects provided more detailed information about the privacy and confidentiality of users’ personal data (e.g., King’s College London’s Urban Mind, an application that examines the wellbeing of users who live in cities), when compared to commercial apps or mass research apps.

Interestingly, many privacy policies, such as those for DNA Fit, also contained instructions not to share login information with others: “You must keep your account credentials secure and not share them with anyone.” This functions as a type of burden shifting by putting privacy and confidentiality obligations back onto the user, rather than being an obligation incumbent to the app developers.

### Commercialization

36/46 applications refer to data commercialization or direct marketing to users in their privacy policy or website information. Where commercialization was addressed, it was often stated that only marketing-relevant data would be shared for advertising purposes (Tables 12 and Appendix 2). However, there were vastly different approaches to commercialization between applications. Commonly, there was a disclaimer that personal information would be used for analytics and advertising to “better serve” the user (e.g., Ancestry, Gene Doe). In a rare case, the opposite occurred, as there was a clause explicitly stating that the data would not be used for advertising purposes (e.g., Ada- Check your Health). Another approach taken by some applications allowed users to object to data processing for direct marketing purposes (e.g., Gene Planet). Others went even further with their data collection by compiling information from publicly available social media profiles for marketing purposes to “maintain and improve the accuracy of the information we store about you” (e.g., Pattern Health). Ultimately, there were few patterns within commercialization sections, with some actively seeking information for marketing and others explicitly prohibiting the practice.

**Table 12:** Applications that discuss data commercialization

1. Ada – Check your Health	
2. Ancestry: Family History and DNA	
3. Apple Research	
4. DNA Nudge	
5. Gene Doe	
6. Gene Planet	
7. My Toolbox Genomics	
8. MyGeneRank	
9. Pattern Health	
10. StuffThatWorks	
11. Withings Health Mate	
12. ActiveDay – Activity Study	
13. ADHD – Cognitive Research	
14. Atlas Health	
15. Behavidence Research App	
16. Chemo Brain Cognitive Research	
17. Depression Cognitive Research	
18. DNA Fit	
19. Dyscalculia Cognitive Research	
20. Dyslexia Cognitive Research	
21. Fibromyalgia -Research	
22. Google Fit	
23. Happiness Project- Play Games for Science	
24. Healthy Minds Program	
25. Hevy Gym Log Workout	
26. Huawei Health	
27. Inside Tracker	
28. Insomnia Cognitive Research	
29. Medisafe Pill and Med Reminder	
30. MyTherapy Pill Reminder	
31. NeuroPsy Research	
32. Parkinson’s Cognitive Researc	
33. Renpho Health	
34. Smart Omix by Sharecare	
35. Symptom and Mood Tracker	
36. Symptomate – Symptom Checker	

### Access to Information, Erasure, and Return of Findings

#### Right to Access Information

While 40/46 privacy policies mention the right to access personal information, return of findings was the most region-dependent section of privacy policies (Tables 13 and Appendix 5). User rights, such as the right to obtain and access information, were contingent on data protection legislation (e.g., GDPR, PIPEDA). Certain applications created a general “right of access” for all users within the privacy policy (e.g., DnaNudge), while others disclaimed that these clauses only applied to users in specific regions whose data protection legislation required this access (e.g., Gene Doe that explicitly states that right to access, rectification, and cancellation may be exercised “if within the EU”). Although nearly all applications contained a mechanism to access information, most of these required reaching out to a specified email account, which often was not easily found in the privacy documents and required a deep dive into the application’s website.

**Table 13:** Applications with right to access personal information (to varying degrees)

1. 23andMe – DNA Testing	
2. Ada – Check your Health	
3. Ancestry: Family History & DNA	
4. Apple Research	
5. CovidWatcher	
6. DNA ID, Inc.	
7. DnaNudge	
8. Gene Doe	
9. GenePlanet	
10. Mass Science	
11. My Toolbox Genomics	
12. MyGeneRank	
13. StuffThatWorks	
14. Urban Mind	
15. Withings Health Mate	
16. ADHD – Cognitive Research	
17. Andaman7 Private Health Record	
18. Atlas Health	
19. Behavidence Research App	
20. Chemo Brain Cognitive Research	
21. Depression Cognitive Research	
22. DNA Fit	
23. Dyscalculia Cognitive Research	
24. Dyslexia Cognitive Research	
25. Fibromyalgia – Research	
26. Google Fit	
27. Happiness Project- Play Games for Science	
28. Healthy Minds Program	
29. Hevy Gym Log Workout	
30. Huawei Health	
31. InsideTracker	
32. Insomnia - Cognitive Research	
33. Medisafe Pill & Med Reminder	
34. MyTherapy Pill Reminder	
35. NeuroPsy Research	
36. Parkinson's Cognitive Research	
37. Renpho Health	
38. Smart Omix by Sharecare	
39. Symptom & Mood Tracker	
40. Symptomate - Symptom Checker	

#### Right to Erasure

While the right to access personal information, even if made difficult, was often included, the right to erasure and the right to be forgotten were more contentious. Some applications stated that while users may have the right to revoke their consent, previously collected data could continue being used. This was seen with My Toolbox Genomics who very clearly state: “Toolbox may not be able to completely remove all of Personal Information about a particular user from its systems”. Others applications were seemingly more open to the right of erasure, but “under certain conditions” that were not specified (e.g., Mass Science). Notably, some apps, such as OH Data Port and Pattern Health, failed to mention anything concerning erasure of data.

#### Right to Return of Findings

Finally, some applications granted users the right to have their findings returned. This right, if found, was always expressed within the privacy policy. The results of research findings, when discussed, were to be published in scientific journals where users could see the results (e.g., NeuroPsy Research, Happiness Project, Google Fit, and Urban Mind).

### General Observations

Overall, many of privacy policies we reviewed were understandable to a layperson, but there was a great deal of variation in how this information was presented. There is also an open question as to the likelihood of a layperson reading a mobile application’s privacy documents. Some applications (e.g., Apple Research) contained a shortened and digestible version of their privacy policy, as well as an extended version for those wanting to know more, increasing the likelihood of the user informing themselves on the application. Many privacy policies were also framed as “FAQs” that were easier to navigate, yet these FAQs generally left out important information.

Conversely, there were 4 applications with non-existent privacy policies, links that led to broken websites, or privacy policies that were unavailable in English. In the case of Expanseeker, the policy was written entirely in Dutch with no option of translation. Moreover, there was a general lack of uniformity or consistency between policies. This makes it difficult to use buzzwords to search within extensive, wordy texts. Information would frequently be entirely missing, which made it challenging to fill the App Atlas with relevant information. For users, trying to navigate these privacy policies would be even more difficult. To properly inform themselves, users must learn how each application organizes their information or read the entirety of the policy. The App Atlas lays out trends and keywords that will help users and researchers more easily navigate these complex and varied documents.

## Discussion: Relevant Themes/Issues

### Privacy/Confidentiality

A central concern that has emerged with the increased use of mHealth applications is the safeguarding of individuals data (Baxter et al. 2020). The risks associated with a lack of data regulation may be especially pronounced for vulnerable groups. “Dataveillance,” a term coined by Degli Esposti (2014), describes the use of data systems to monitor certain people or groups in order to influence or govern their behaviours. As a result, even if information remains de-identified, patterns may be spotted within subgroups that can render them subjects of targeted promotion despite their “anonymity” (Degli Esposti 2014). Data breaches are also becoming increasingly common, causing consumers to be concerned about the sensitive information being collected in mHealth applications. Conversely, others argue that further mHealth regulation could inhibit innovation (Lynch and Fisk 2017). Nonetheless, the lack of clear regulation in this area has caused a wide range of approaches to privacy and confidentiality, leaving the users ill-informed as to how their data is used in these apps. Even where there are regulations in place, such as the GDPR in the European Union, these regulations may be difficult to enforce where data is merged between several sources and servers around the world, as is often the norm with mHealth applications (Gerl 2019). Finally, while consent is critical in this arena, fully informed consent is difficult to achieve, particularly given the complex language used in privacy policies and their long formats (Theis et al. 2023).

Anonymizing and aggregating personal information were one of the most prevalent approaches to data sharing, consent, and privacy. Given that regulations such as PHIPA, HIPPA, and the GDPR primarily apply to identifiable personal information (Phillips and Knoppers 2016), anonymization effectively allows applications to use the de-identified information as they choose, while the user can be assured that their identity will remain protected. Yet this approach is not entirely satisfactory. For example, to address algorithm-based biases, identified information is required. This presents a paradox, because to be able to check against bias, more user information, such as age, gender, or socioeconomic status, may have to be collected. However, the more information that is collected, the higher the risk of “dataveillance” and “re-identification” (Degli Esposti 2014). Developers must be aware of bias in their algorithms as algorithms embedded in applications, such as symptom checker apps, can impact the daily lives of the users. If the algorithm is primarily based on data that underrepresents certain groups, the application must be aware of if a user falls into an underrepresented groups and should aim to train the algorithm to better meet the needs of its users. However, collecting identity information places users at a higher risk of being re-identified and targeted. These issues are further complicated by the reality that there is no clear definition of what constitutes “de-identified” data (O'Keefe and Connolly 2010). One possible approach to remedy this ambiguity and increase transparency was proposed by the National Statement on Ethical Conduct in Human Research in Australia. This statement proposed that the term “de-identified data” be replaced with the terms individually identifiable, re-identifiable, and non-identifiable data. The use of these terms would provide a better picture of the risks of re-identification (O'Keefe and Connolly 2010).

In addition to the challenge of algorithm training and data re-identification, data de-identification also raises a consent issue. Current legislation such as the GDPR treats de-identified data differently from personal data. This distinction is often reflected in the privacy policies. While Canada’s PHIPA Decision 175^1^ has re-asserted that information custodians must ensure that de-identified information remains de-identified, even in the case of a sale, studies have shown that permanent de-identification has become increasingly difficult with current technological advances (Rocher et al. 2019). This is particularly pertinent for genetic applications. Of the applications examined, only one, OH Data Port, addresses the near impossibility of truly “de-identifying” personal information, particularly in the case of genetic information. In a clearly highlighted disclaimer, OH Data Port warns that even if a pseudonym is used, data is often easily identifiable. They further indicate that merely providing a birth date, sex, and ZIP code would be enough to identify a person in an anonymized dataset. Additionally, they warn that genetic data, which is effectively impossible to de-identify, and location data possess an increased risk of re-identification. While these disclaimers do not entirely protect users from having their information identified and used against their consent, they at least warn of the potential risks of participation, which remains a significant element of consent.

### Comprehensibility/Transparency

A key limitation of including critical information within privacy policies is that most people do not read lengthy privacy policies before using applications (Obar and Oeldorf-Hirsch 2020). Many terms are overly legalistic, technical, or scientific, which poses a challenge to their comprehensibility. Das et al. (2018) underwent a readability analysis of privacy policies, which revealed that the majority are incomprehensible to the general population and required college-level literacy to understand. This is particularly problematic for applications that are targeted towards people with learning or neurological disorders. Even when a “term definition” section was included in the privacy policy to increase comprehensibility, this glossary was typically difficult to use while reading the document.

Information presented to users either in the app description or website was often more comprehensible, albeit limited. Many of the websites simply redirected users to read the full privacy policy. An alternative approach was a Frequently Asked Questions (FAQ) section that summarized privacy information. FAQ sections appeared to be the easiest to navigate and understand. Nonetheless, these FAQs frequently overlooked information concerning the user’s privacy. Additionally, it is important to remember that the information displayed on an app’s website is often reflective of features the application’s maker found worthy of advertising. This was observed in the App Altas, where 8 applications that conducted research but did not mention it on their websites. For advertising purposes, it was more convenient to gloss over their research activities. Interestingly, while privacy policies mostly contained similar information, the full picture of informed consent and privacy considerations were rarely displayed on app websites, particularly if they did not portray a favourable image of the company. The websites and app descriptions often presented an oversimplified version of the application’s activities.

There are also clear issues with transparency when applications discuss local privacy legislation. For example, while rights under the GDPR are often reiterated for EU citizens in privacy policies, it was often unclear what rights apply to non-EU citizens. Nonetheless, these rights were clearly explained when they were included. This is a compelling demonstration of the impact that robust privacy laws can have on user rights and transparency.

In addition to information being difficult to locate across websites and privacy policies, almost all privacy documents analyzed for the App Atlas contained ambiguous or contradictory language. While privacy policies often distinguished between personal information and anonymized information, it was unclear which provisions applied to each type of data and whether consenting to research included consenting to the sharing of personal information. Consent is called into question when the provisions themselves are unclear or contradictory.

Certain claims contained in the privacy policies of these applications were also questionable given the nature of the application. For example, Urban Mind claims that the app does not collect any information that is identifiable, yet they collect age, gender, occupation, and lifestyle information, which is information that could identify a person. This heightens the risk of re-identification of information and may be especially concerning to users. One can imagine a situation where app users who consider large technology companies untrustworthy have their data unknowingly transferred to an unintended audience, the tech giants, in the course of an acquisition of the app they use (Robillard et al. 2019).

The situation is further complicated by the fact privacy policies are not explicit about the overall purpose of the application. A study by Sunyaev et al. (2015) found that two thirds of mHealth privacy policies do not mention the app itself, as they are often generalized privacy policies used across multiple apps or products. This limits app transparency, because users may not truly know the nature of the app they are downloading and how much of their information is collected in the process. This may have serious consequences for users, including unwanted targeted advertising or privacy breaches (Robillard et al. 2019). A potential solution to this is the creation a certification standard for mHealth apps, such as the one created by the National Health Service Health Apps Library (Robillard et al. 2019). A tool, such as the App Atlas, will also be useful in providing an overview of the pre-existing approaches to privacy policies and where there are gaps in need of regulation.

### AI/Algorithm Considerations

Many mHealth applications implement algorithmic/artificial intelligence systems to provide their users with tailored information, while simultaneously incorporating that user’s information into their program. There lacks a clear definition of artificial intelligence (AI) in the mHealth application world. Of the 46 applications examined in this paper with available privacy policies, 23 claim to have some level of artificial intelligence algorithm embedded into their use. Yet only 8 contain any mention of algorithms or artificial intelligence in their privacy policies (Appendix 6 and 7).

AI poses a unique challenge, as it may embed biases within its programming. Moreover, as this technology advances, higher accuracy in AI medical predictions may exacerbate medical discrimination. Furthermore, while legislation, such as the GDPR, has made advancements in highlighting the importance of user consent to information use, anonymized data is not regulated by the GDPR and this data can often be re-identified (El Emam 2019; Rocher et al. 2019). This raises concerns regarding privacy, confidentiality, data security, and possibilities of discrimination.

The risk of data re-identification varies based on the application’s purpose. Often, tracker apps require the least amount of information, as they are targeted to tracking activities such as taking medicine or exercise frequency. However, genetic apps pose the highest risk, as users provide the application with genetic information that cannot truly be anonymized. While genetic applications were also the most likely to outline extensive data protection policies, many of their safeguards and consent provisions are nonetheless centered around “anonymization,” “pseudo-anonymization,” “de-identification,” and “aggregation.”

Although, AI poses a certain set of challenges, including data privacy risks and bias, it is worth noting that these challenges are not unique to AI and may even be better managed by an AI. Yet AIs are also built by humans, and ultimately reflect the nature of human bias. As such, there are few unique risks an AI can present that would not have been presented by the humans that built it. Similarly, data privacy risks are present regardless of whether an AI is used. Therefore, there may not be a strong enough difference between human and AI-based research to legally require the demarcation of AI use in privacy policies, reflecting most applications’ decision to group algorithm use within broader research statements. Nonetheless, the strongest reason for including AI in privacy policies explicitly is for informed consent purposes, as users should be able to know and assess their own comfort levels before using these applications. The 8 apps that explicitly mention their algorithm/AI provided the most transparency in how and what type of user data is processed.

### Improving the Readership of Privacy Policies

Despite the privacy concerns raised by inconsistent and ambiguous privacy policies, another challenge persists: users are simply not reading privacy policies. In a study conducted by dos Santos Brito et al. (2013), only 4% of users consistently read privacy policies, with 55% having never done so. Even if privacy policies contained the necessary information to facilitate informed consent, their length and language would nonetheless make them fail in their objective in informing users (Tesfay et al. 2018).

Yet, length and language do not entirely address the lack of readership, as even when privacy policies are written at a user’s comprehension level (e.g., accessible FAQs), readers nonetheless demonstrate poor comprehension of the information presented (Vu et al. 2007). This is due to user attitudes that also play a significant role in their likelihood of reading and understanding privacy policies (Ibdah et al. 2021). In a study conducted by Ibdah et al. (2021), 75% participants reported feeling negatively about the way privacy policies were designed and the content they embodied. There was a general sense of resignation among users, claiming that they “live in an age where privacy is gone. We can pretend we have control with opt outs, but we really don’t” (Ibdah et al. 2021). The lack of readership is therefore both due to unclear content and attitudes of resignation and acquiescence.

Therefore, improving user trust is necessary for improving readership and is in the best interest of developers and researchers who wish to use crowdsourced data from these applications. This is supported by literature that highlights a correlation between the understandability of privacy policies and user trust (Ermakova et al. 2014). The stronger a user believes to have understood the privacy policy, the more they reported trusting the website. Moreover, participants in a study conducted by Meier et al. (2020) demonstrated better understanding of privacy policies when the policy was shorter. A short privacy policy also improved their subjective privacy perception, increasing their self-disclosure. The length of privacy policies is therefore highly relevant to understandability and building trust. For example, Google’s privacy policy, which was 600 words when it was merely a search engine, is now 4000 words to encompass the many practices the company engages in today (Ibdah et al. 2021). In the sample examined in the App Atlas, longer privacy policies were more likely to have contradictions, or unfavourable information “hidden” within the policy. A privacy policy being too long was also the most common reason cited by college students as to why they do not read privacy policies (Moallem 2017). These issues are even further exacerbated for non-Anglophone users of health apps, as the high reading levels required coupled with the unavailability of many privacy policies in non-English languages limits the cognitive accessibility of these privacy policies (Neal et al. 2023).

Additionally, increased control over privacy would further increase user trust and, correspondingly, their likelihood of reading the privacy policy (Aïmeur et al. 2016). Application design often presents little choice for the users. The privacy policies mandate disclosure of personal information in order to use the applications. If the user does not want to disclose personal information, their only choice is to not use the application. Instead, privacy policies should give users a choice of what information they wish to share and should enable the user with choices regarding their privacy (Bashir et al. 2015). With the current approach to privacy policies, any consent acquired from the user cannot be considered “informed,” because it was not truly voluntary. Rather, user consent is coerced, in a way, as the option is consent or do not use the application. In fact, in a recent study by Bashir et al. (2015), 81% of users reported that they had submitted information online when they did not wish to do so. This underscores the perception of coercion in the current privacy policy frameworks. Ensuring informed consent and enabling users with a choice would improve negative perceptions of privacy policies and better allow readers to understand specific clauses they are consenting to, rather than merely checking a box at the bottom of a lengthy text. Providing users with specific choices throughout the privacy policy would therefore be a better way of improving readership and achieving informed consent, both for the sake of the users and for the researchers who wish to use data compiled by mHealth applications.

Legislation promoting further uniformity and comprehensibility of privacy policies would also establish norms that would ensure that relevant information is provided within privacy policies. If the public is aware of legislation that governs privacy policies, users would be much more trusting of privacy policies. Furthermore, machine learning could also play a role in condensing long privacy policies into more understandable language (Tesfay et al. 2018). These factors should be taken into consideration by developers and policy makers, as improved readership will create a self-reinforcing cycle with trust, self-disclosure, and informed consent.

## Conclusion

mHealth applications are ever-popular and pose a significant challenge to user privacy, as many users remain unaware of how their sensitive health information may be used and shared. User data stored by these applications is also highly valuable. The information regarding users’ data is complex and difficult to understand. It is also provided by developers in various locations—privacy policies, app descriptions, and app websites. Taken together, these facts raise concerns regarding privacy, trust, transparency, comprehensibility, and readership. This was the primary challenge the App Atlas aimed to confront. As mHealth application privacy documents lack uniformity, this study classified information contained in these documents for 46 mHealth applications. In doing so, the Atlas allows app users and policy makers to more easily read mHealth app privacy information and observe what is missing, contradictory, or ambiguous. By condensing these policies into the App Atlas, this study provides an example of how relevant information can be presented to better facilitate informed consent.

While we have aimed to provide increased legibility and organization to these policies, the rapidly changing nature of the app store renders it difficult to maintain up-to-date databases on mHealth applications. This study organizes the privacy information found regarding mHealth apps but does not analyze the comprehensibility of privacy policies. While categorizing data -sharing information within the App Atlas will help users look for key words to find this information, it does not necessarily make this information more understandable to the lay reader. Additionally, while the App Atlas is organized under subheadings, these were often difficult to apply equally across all applications, as there were inconsistent definitions of research, consent, and AI. Moreover, much of the information had to be condensed, potentially altering the full picture of the app’s privacy policy. Another restriction of this study is that it only examined 46 applications. While this provided a preliminary sense of what types of inconsistencies and ambiguities were reoccurring, it cannot be representative of all mHealth applications. Future studies may improve the representativeness of this sample, as well as the overall comprehensibility of information. Overall mHealth applications would benefit from simplifying the language used in their privacy policies as it would help users and researchers have a clearer image of the privacy landscape in mHealth applications. This would prompt users and researchers to be more trusting of these applications, especially considering that scientific studies involving humans typically have requirements of ethical data collection and informed consent.

### Supplementary Information

ESM 1 (XLSX 101 kb)

ESM 2 (DOCX 36.3 kb)

ESM 3 (DOCX 23.0 kb)

ESM 4 (DOCX 37.0 kb)

ESM 5 (DOCX 29.2 kb)

ESM 6 (DOCX 27.3 kb)

ESM 7 (DOCX 17.0 kb)

ESM 8 (DOCX 17.8 kb)

## Footnotes

1 https://decisions.ipc.on.ca/ipc-cipvp/phipa/en/item/520967/index.do?q=phipa+175.
