# Supplementary material for: Mapping the Apps: Ethical and Legal Issues with Crowdsourced Smartphone Data using mHealth Applications
Source: Asian Bioeth Rev. 2024 Jun 18;16(3):437–70. doi: 10.1007/s41649-024-00296-3 (PMC11250705; doi:10.1007/s41649-024-00296-3)
Supplement: Supplementary file 2 — (DOCX 36.3 kb) [file 41649_2024_296_MOESM2_ESM.docx]

| Appendix 1: Mentions of Research | |
| --- | --- |
| App | Research |
| 23andMe - DNA Testing | 23andMe has an opt-in research program, meaning that for eligible customers, taking part in 23andMe Research is completely voluntary.  What is 23andMe Research? The purpose of 23andMe Research is to make new discoveries about genetics and other factors behind diseases and traits. “23andMe Research” means research activities performed by 23andMe, either independently or jointly with third parties, and overseen by an independent ethics review board (also called an Institutional Review Board or “IRB”). 23andMe Research may be sponsored by, conducted on behalf of, or in collaboration with third parties, including non-profit foundations, academic institutions or pharmaceutical companies.  What if I do not want to participate in Research? If you are eligible to participate in Research, you choose whether to participate or not, and you can change your mind any time. Customers never need to participate in Research to use 23andMe. Nothing changes about your core 23andMe experience if you do not participate in Research. We do not use your information for Research unless you explicitly choose to participate in Research.  How does 23andMe protect my information in Research? 23andMe Research analyses are conducted with information that has been stripped of your identifying Registration Information.   If you choose to consent to the Main Research Consent... - Your de-identified Genetic Information and/or Self-Reported Information may be used for Research. - We may use de-identified individual-level Genetic Information and Self-Reported Information internally at 23andMe for research purposes. -We may share summaries of research results, which do not identify any particular individual, with qualified research collaborators and in scientific publications. -We may inform you of research opportunities for which you may be eligible. We will not share individual-level Personal Information without your explicit consent.  Some participants choose to contribute in additional ways to Research. For example, you can choose to participate in Individual Level Data Sharing, or additional study-specific agreement(s). Those consents are separate and, like the Main Research Consent, you can withdraw from them anytime.  From Website:  As a customer, you will be asked if you would like to opt into research. More than eighty percent of our customers choose to participate. You can opt into or out of research at any time.  Our research activities are reviewed by an independent ethics committee, also known as an Institutional Review Board (IRB).  When you participate in our research program, your data is de-identified. That means your registration information is stripped from your genetic data so you cannot reasonably be identified. We do not include any of your registration information (like your name, email, etc.) in our research database. We store personal information that researchers do need for analysis (like date of birth) in a separate database from the genetic data, and they are only linked by a randomly assigned research ID.  Most of our research is performed using aggregate-level data. That means researchers link your de-identified data with millions of other data points and look at the data set as a whole. We do not share your individual-level information with any third party without asking you for (and receiving) your explicit consent.  All research activities conducted by 23andMe Research are governed by an Institutional Review Board (IRB). The IRB is an independent ethics panel that ensures all research is conducted in accordance with government and ethical guidelines. |
| Ada - Check your Health | We may collect and process personal data that you will be asked to provide when you: [...] complete any surveys or provide any feedback that we may use for research and improvement purposes (although it is optional, and you do not have to respond to these if you do not want to).  We use the above data to assess your suitability for clinical research and to reach out in order to invite you to partake in clinical research with one of our clinical research partners that may be of interest to you and/or to refer you to health services for follow-up diagnostics. For the avoidance of doubt, we do not pass on any personal data to our clinical research partners without your consent and any participation to such clinical researches and referrals to health services are optional and subject to your prior consent. Use justification: Consent (Article 6(1)(a) and Article 9(2)(a) GDPR when you consent to participate to such clinical researches and that your data needs to be processed for the aforementioned purposes. You may revoke/withdraw your consent at any time (more information about your data subject rights in section 8 below).  We process pseudonymized data to carry out aggregate statistics on the geographical prevalence of certain types of illness symptoms and conditions and may present such summarized statistics to our partners, always on an irreversibly anonymized basis. Use justification: The processing is necessary for statistical purposes and we only provide our partners with anonymized and summarized statistics from which the identification of a specific natural person is impossible (Article 9(2)(j) GDPR; Sec. 27 (1) BDSG). Our legitimate interest in processing data for these purposes is to support progress in medical research in line with our entrepreneurial goals which is also in the public interest to improve healthcare such as, but not limited to, analyzing the occurrence and characteristics of diseases.  We may disclose certain data to organizations involved in clinical trials and other types of research where you have explicitly authorized us to do so.  From Website (AI): How Ada’s AI works Originally developed to support clinical decision-making, our doctors created Ada to think like a doctor. Clinical precision and medical oversight from human doctors has been in our DNA since the very beginning. Meet some of our medical experts. |
| Ancestry: Family History & DNA | We use your Personal Information to provide, personalize, improve, update, and expand our Services. This includes: Issuing surveys and questionnaires for use in the Services, as well as facilitating product development and research initiatives; Conducting scientific, statistical, and historical research;  We share your Genetic Information with research partners only when you provide us with your express consent to do so through our Informed Consent to Research. Unless you agree to the Informed Consent to Research, your data will not be shared with these researchers. Research partners include commercial or non-profit organizations that conduct or support scientific research, the development of therapeutics, medical devices or related material to treat, diagnose or predict health conditions. In some circumstances, a research partner or Ancestry may have a financial interest in the research arrangement. A list of our research partners can be found here.  Ancestry may disclose user information in an aggregated form as part of the Services or our marketing, or in scientific publications published by us or our research partners. For example, we might note the percentage of immigrants in a State that are from a particular geographic region or country. Such disclosure will never include Personal Information. |
| Apple Research | Informed Consent To participate in any Studies, you must first review and sign an informed consent and authorization form (if applicable) ("Informed Consent") for the Study. By using an App, you consent to the collection, use, and sharing of your information as described in this Privacy Policy and as outlined in the Informed Consent.   From Website: Descriptions of studies  1. Join the Apple Women’s Health Study, conducted in partnership with the Harvard T.H. Chan School of Public Health and National Institue of Health Sciences to help advance the understanding of menstrual cycles and health conditions such as infertility, osteoporosis, and menopause.  2. Join the Apple Heart and Movement Study, conducted in partnership with the American Heart Association and Bringham and Women's Hospital to help advance discovery in heart science and to help us learn how activity and your habits can contribute to a healthier heart.  3. Join the Apple Hearing Study, conducted in partnership with the University of Michigan to help advance the understanding of how sound exposure levels over time can impact your hearing, stress levels, and cardiac health. |
| CovidWatcher | Study Purpose CovidWatcher is a research study at Columbia University to understand the development of the coronavirus COVID-19 pandemic, its symptoms, its impact on individuals’ attitudes, behaviors, and daily lives. The study is a partnership at Columbia University between the Department of Biomedical Informatics, the Division of Infectious Diseases in the Department of Medicine, and the Urban and Social Policy Program at the School of International and Public Affairs. If you consent to participate, information gathered through the app will help crowdsource valuable information about the pandemic, such as its potential next hotspots. This information can help prepare cities, public-health officials, and healthcare institutions in their response to COVID-19, as well as inform individuals about their communities.  Data Gathering CovidWatcher will gather profile information, answers to short daily and weekly questionnaires (e.g., Covid symptoms). If you allow it, it will also gather HealthKit information, as well as in and out geofence activity. CovidWatcher will request your permission to read from your phone’s HealthKit to get access to data such as steps, heart rate, and body temperature. You can revoke permission at any time from the Health app.  From Website: CovidWatcher is a research, advocacy, and policy platform developed by Columbia University in collaboration with NYC community partners. We need you to respond to the CovidWatcher surveys. Your responses will help government, community organizations, and hospitals identify needs, target resources, and develop more effective policies to ensure the health, safety, and economic vitality of our City as we rebuild from the COVID-19 pandemic. Surveys can be taken via our website, on desktop or mobile, as well as via the CovidWatcher app- Available on iOS or Android |
| DNA ID, Inc. | We only share the data that you want to share! Your DNA is the baseline for establishing a share, but remember you can always say no to sharing.  Once you’ve shared your data, you can view your past transactions and an overview of what you shared with the researchers. If you want to withdraw from a study, just send us an email at support@dnaid.co .  Healthcare data is typically owned by a few large institutions where you receive your health care. However, we believe that you should own your own data. Blockchain streamlines how data is managed and ensures security. In addition, blockchain has natural transparency and incentives built in to the technology. Our primary goal is to put healthcare data back in the contributors hands and give them the opportunity to determine how it’s used. |
| DnaNudge | From time to time, we may use automatic data collection technologies to collect anonymised product data for research, development and statistical purposes.  The Purpose For Which We Use Your Data: -To manage your account and from time to time to communicate with you including by sending you promotional offers or other marketing information or to invite you to participate in surveys, questionnaires or research projects. You can opt out of promotional communications through our Service; -For research and development purposes (including machine learning) in order to improve or personalise the Service and to help us understand our customers and how our Service is used; -To aggregate data to allow it to be used for statistical and research purposes  When disclosing personal data to third parties: We will not disclose the results of such analysis or research to third parties or publish it except in the form of Anonymised and Aggregated Data. |
| FLARe Research | N/A from Privacy Policy  From Website:  FLARe aims to model the processes underlying the development, maintenance and treatment of anxiety disorders, and post-treatment relapse.  Some people learn very quickly that certain things make them feel anxious, whereas others are less likely to experience these feelings. Similarly, there is variation in how quickly anxiety reduces following a stressful experience, and in how successfully people who are anxious respond to exposure based treatments.  In this study, we are interested in how people learn to be anxious of new things, and how they learn that some things that make them feel anxious might not always be unpleasant. We are also interested in the ways in which genes and the environment might contribute to this.  We have developed and validated a smartphone app that delivers a fear conditioning experiment remotely. We also assess a range of other anxiety-related features, such as particular patterns of thought. We ask volunteers to take part in this app-based experiment to help us understand the different role of genes and the environment on how anxiety develops, and how individuals respond to psychological treatment. |
| Gene Doe | N/A |
| GenePlanet | GenePlanet can process Personal Data for research purposes to gain new potential insights/findings in science (e.g., novel genetic variant role discovery).  By giving Consent, you agree that your Unused Biological Sample and Personal Data can be saved and further processed for the purpose of research. Your Unused Biological Sample and Personal Data will be stored in pseudonymised form until you request their destruction. In cases when they are used for research purposes, they will be anonymised.  Data processed: relevant personal data is obtained from GenePlanet's purchased Product analysis  Legal Grounds: Consent Retention Period: Until your request for deletion and destruction |
| Mass Science | Mass Science collects your data so that we can analyse it in order to answer important questions on:  The effect of the Covid-19 outbreak on mental health The symptomology of Covid-19 The prevalence and spread of Covid-19 If you agree, our group will share an anonymised copy of your data with other research groups at the conclusion of the study. |
| My Toolbox Genomics | “Toolbox Research” means analysis by Toolbox of its Services, the Toolbox website, Aggregated Genetic and Self-Reported Information, its platform, and efficacy with its users. Toolbox Research only uses individual Genetic Information and/or Self-Reported Information from users who have given informed consent according to an applicable consent document.  We use Aggregated Genetic and Self-Reported Information for the following purposes:  -For Toolbox Research; -For other research for which you have given consent.  Disclosure of information: If you have given consent for Toolbox Research, other research (including third party research partners), or research contractors to access your Genetic Information and Self-Reported Information online and at Toolbox’s offices for scientific research purposes, provided that any third party research partners and research contractors will be screened and supervised by Toolbox, and will be subject to the rules, policies, and guidelines established by Toolbox; |
| MyGeneRank | The MyGeneRank website and online services collect your Personal Information for the purposes of genetic research studies being conducted by the Scripps Research Translational Institute. If you choose participate in MyGeneRank studies and provide us with any Personal Information, you must first review and electronically sign the Informed Consent.   We may collect, store, and use the following kinds of Personal Information: Information that you provide when providing Informed Consent for MyGeneRank research studies, including your name and signature. Your informed consent document, containing your name and signature, as well as your email address, is stored electronically in a secure data repository separate from all other Personal Information.  Information collected during participation in MyGeneRank research studies. The Informed Consent associated with each research study will tell you what information will be collected. If you choose to share information during participation in MyGeneRank research studies, once that information is collected, it is covered by this privacy policy. This information includes:  Information that you manually enter during participation in MyGeneRank research studies (e.g. survey responses). This may include personal health and demographic information such as blood pressure measurements, cholesterol levels, family health history information, age, gender, weight, etc. You may choose not to provide us with certain information.  Information that is automatically collected during participation in MyGeneRank research studies, including data collected by HealthKit (e.g. step counts, nutrition logs, etc.), and data collected by wearable technology (e.g. wristwatches, blood pressure monitors, etc.).  We will combine the information collected from you during participation in MyGeneRank research studies with information collected from other study participants. The combined information will be used by researchers to determine the relationship between genetic information and health and behaviors  We may share your coded study data (data without your name, date of birth, or email address) with other researchers as permitted by the informed consent for MyGeneRank studies. Individual level genetic data collected by the MyGeneRank website and online services will not be shared. Aggregate genetic data and statistical results may be shared.  From Website: Contribute to science: by participating you contribute to the cutting edge of genetics and medical technology.  About the Study The goal of this study is to determine how your genetic risk influences health decisions and other things that can be controlled in life. Our first genetic risk score is calculated for coronary artery disease (CAD). etc |
| OH Data Port | Sharing data is critical to scientific progress, but has been hampered by traditional research practices. Our approach is to invite willing participants to share their personal data for the greater good. Open Humans strives to make this possible by helping connect researchers and participants who wish to share data with each other.  You may also choose to share data directly with studies and other projects run by third-party researchers, other Open Humans members, or the general public. Third parties are responsible for their own data practices. |
| Pattern Health | Through use of Pattern Health applications your User-Provided Information is shared:  within the Pattern application: -With your healthcare provider and/or research team. -With the Apple Health app (privacy policy), if enabled by you via More tab, Connected Devices, Apple Health App within the HeartStar application: -With whoever you choose, by generating a report of the data and emailing it -With the Apple Health app (privacy policy), if enabled by you via the User tab, Sync and Share Data, Sync with the Apple Health app  From Website: Pattern powers clinical trials and digital interventions, accelerating health innovation. Our technology supports academic and commercial-sponsored single-site, multi-site, decentralized, and hybrid clinical trials. Our platform can improve trial participation, engagement, and efficiency.  Data sharing:  Website provides a list of integrated programs |
| Project Serotonin | We reserve the right to use Anonymous Data and aggregated and other de-identified information for any purpose and disclose Anonymous Data to third parties at our sole discretion, including for research purposes.  For data deletion requests: When closing an Account, we remove or delete Personal Information associated with that Account, subject to certain limitations stated below:  -Information already used for Research and Study Participants; and -Limited information in de-identified, pseudonymized, or aggregate forms used in Research, data analysis and artificial intelligence. |
| StuffThatWorks | We may use information that we collect about you for the following purposes:  -To perform and provide scientific and medical research, and improve treatment options for patients, using deidentified and/or aggregated data. With your consent, we may share your information with our Partners in the industry, government and/or academia for research purposes, as more fully described below   We will not share your personal information without your explicit consent, with our valued partners, including but not limited to those in the medical, pharmaceutical and biotechnology industries, academic institutions, and government agencies and regulatory bodies, including regulatory bodies such as the American CDC and FDA, or other national and international bodies, as applicable and as necessary. We may share only de-identified and/or aggregated information with Partners, in order to conduct scientific, and/or medical research, as part of our Service. When disclosing information to our Partners or otherwise selling user information for scientific or market research purposes, we make sure to anonymize and/or remove all Personal Information or other personally-identifying indicators in the data (de-identification) to minimize the possibility of accidental member identification. |
| Urban Mind | The information you provide will be used for academic research to understand the impact of the urban and rural environment on mental health and wellbeing, to inform novel clinical interventions and for the planning and design of healthier cities.  The data may also be used for other research purposes in the future. The results will be published in scientific journals and presented on our web site dedicated to the project (www.urbanmind.info) and social media. If you post publicly about Urban Mind using social media, or communicate directly with us, using the Urban Mind website or on a social media website, we may collect and process the data contained in such posts for the purpose of addressing any requests you may have and for public engagement connected with the research project.  Your participation in the Urban Mind research project is anonymous, private and confidential. The information you provide will remain with King’s College London, and may be shared securely with the project partners (J&L Gibbons and Nomad Projects) and with other organisations collaborating on the project for research purposes. The information you provide may also be shared securely with organisations who provide services to us in connection with the research purposes, for example data storage, as stated in this Privacy Policy.  From Website:  If I agree to participate, what information do I need to provide? When you first use the Urban Mind app, you will be asked to complete an initial questionnaire about your age, gender, occupation, lifestyle and wellbeing. You will then be asked to answer questions about your current environment, feelings, thoughts and behaviour, 3 times per day over a period of 2 weeks. Participation in the study is private and confidential and your entries will be anonymous. You will not be asked to provide your name, phone, e-mail address or any other information which could identify you.  Throughout the 2-week trial, we will acquire information about your geographical location using the GPS sensor on your mobile phone. You can opt out of this feature if you prefer. We will also acquire information about your physical activity from your smartphone – your step count, distance travelled and whether you are walking, running or cycling. You can also opt-out of this feature if you prefer. We will ask for your consent to provide your geographical location and physical activity data when you first use the Urban Mind app. Your consent to provide this data can be changed at any time using the ‘Settings’ menu in the app.  At the end of each assessment you will have the option of taking a photograph of the ground or floor where you are standing and/or an 8-second audio recording of your current environment. These photographs and audio files will be analysed for research purposes and, furthermore, may be posted on our social media and used for digital art exhibitions to promote the project.  What are the benefits of participating in this study? Your participation will help us understand better the factors in the environment that impact mental health and wellbeing. The results will be used to inform novel clinical interventions and the planning and design of healthier cities.  What are the risks of participating in this study? We do not anticipate any risks to your mental health or wellbeing from participating in the study. Collecting geographic location and information about your physical activity can reduce the battery life on your phone slightly, so that you may have to charge it more often. |
| Withings Health mate | We collect your consent to process personal data for: participating in our research programs.   At any time, you can withdraw your consent, to do so, uncheck research centre in your notification centre.   RESEARCH & DEVELOPMENT  1. Sending out "Research Questionnaires" and analyzing the responses received  PROCESSED DATA: User ID. The content of the questionnaire varies according to the issues addressed BASIS FOR THE PROCESSING: Consent of the person filling in the questionnaire RETENTION PERIOD: Pseudonymized Data is retained until the account removal 2. Anonymization of data for research purposes  PROCESSED DATA: Health data needed to conduct the study BASIS FOR THE PROCESSING: Consent 3. Product and Service Improvement (including algorithm performance improvement and statistics))  PROCESSED DATA: The relevant data related to the realization of these treatments. It is exclusively pseudonymized data BASIS FOR THE PROCESSING: Legitimate interest RETENTION PERIOD: Personal data is kept until the user account is deleted |
| ActiveDay – Activity Study | N/A |
| ADHD – Cognitive Research | We use the information we collect to improve and personalize the Services and to develop new ones. For example, we use the information to troubleshoot and protect against errors; perform data analysis and testing; conduct research and surveys; and develop new features and Services.  A parent, educator, health professional, or researcher may grant you access to the Services and access your personal data, such as your cognitive activity and results. We ask for your explicit consent before sharing your data with such person and we provide you with privacy preferences in account settings and other tools to enable or disable this sharing feature.  Information you have shared with others (e.g. with a parent, educator, health professional, researcher, or some of our partners) will remain visible after you closed your account or deleted the information from your own profile or mailbox, and we do not control data that other users copied out of our Services. Community features content (e.g. challenges) associated with closed accounts will show an anonymous user as the source. |
| Andaman7 Private Health Record | From Website: We offer Andaman7 to doctors, hospitals and researchers who want to improve the effectiveness of treatment and general health care. To do this, we facilitate interactions between them and volunteer patients, and we facilitate access to health data of patients who give their explicit consent. These companies then compensate Andaman7 in the form of a fee to use the platform. |
| Atlas Health | We will use this information: to administer our site and for internal operations, including troubleshooting, data analysis, testing, research, statistical and survey purposes; |
| Behavidence Research App | Whenever we conduct a public-wide research, you may join the research through the designated form. If you register to a research, we collect your contact information, age, existence of diagnosis of mental health condition (in a yes/no question), your mobile device type, and whether you live in the United States. We refer to this as “Research Registration Information”.   You do not have a legal obligation to provide us with your Contact Information or your Research Registration Information. However, we may not be able to respond to your inquiry, to subscribe you to our mailing list, or to include you in our research, if you choose to not share this information with us.  You may withdraw your participation in the research at any time by contacting us at health@behavidence.com. If you ask to withdraw your participation, we will delete all your personal information relating to the research.   From Website:  AI Based Utilizing digital phenotyping and machine learning, we aim to detect a decline in mood and focus and an increase in stress through passive digital biomarkers. |
| Better- Rewards for Health | The nature, purpose, and duration of the research Bowhead conducts research to better understand the triggers, reliefs, symptoms related to migraines as well as how they correlate to other lifestyle habits.  The purpose of this research is to eventually display aggregate community insights to help other migraine sufferers with aggregate community insights.  Rest assured, Bowhead never sells your Personal Information or Personal Health data to third parties. Bowhead plans to conduct this research and use the de-identified data for up to 20 years. You may at any time withdraw your Informed Consent.  Procedures, risks, and benefits to the participants In order to participate in the research you can install the Better by Bowhead app, and you do not have to pay for the App or provide any Personal Information.  You may backup your account using the 12 word recovery backup encryption code. There is a risk if you lose this encryption code we cannot recover your account. You are responsible for the safekeeping and storage of your backup code.  The potential risks are that the app may be taken offline and you may lose your data, we make no guarantees that the app will exist forever. Another risk, is that you see a community member suggest a migraine relief or any treatment which has not been medically validated. Please consult a doctor before making any changes regarding your health.  The potential benefits are that you may learn from the community and better understand how others, in an aggregate manner, are living with a condition. Bowhead may potentially be a useful secure tool to enable you to track your migraine events in order to build a complete picture of your migraines’ triggers, symptoms and reliefs. In no way does Bowhead make the claim of curing, diagnosing or treating migraines and you should speak to your medical doctor before making any adjustments to your health.  From Website Incentivized Research ‍By contributing your health data to research studies, you not only help advance medical science but also earn Anonymised Health Tokens (AHT) that can be redeemed for health-related products and services. At Bowhead, we partner with top researchers worldwide to develop cutting-edge medical breakthroughs using the power of blockchain technology. |
| Chemo Brain Cognitive Research | We use the information we collect to improve and personalize the Services and to develop new ones. For example, we use the information to troubleshoot and protect against errors; perform data analysis and testing; conduct research and surveys; and develop new features and Services.  A parent, educator, health professional, or researcher may grant you access to the Services and access your personal data, such as your cognitive activity and results. We ask for your explicit consent before sharing your data with such person and we provide you with privacy preferences in account settings and other tools to enable or disable this sharing feature.  Information you have shared with others (e.g. with a parent, educator, health professional, researcher, or some of our partners) will remain visible after you closed your account or deleted the information from your own profile or mailbox, and we do not control data that other users copied out of our Services. Community features content (e.g. challenges) associated with closed accounts will show an anonymous user as the source.  From Website: Cancer research is the foundation for improving the lives of people with cancer. CogniFit provides the possibility to study the effect of cognitive stimulation on chemo brain through the researcher's platform. In addition, the CogniFit Platform for Researchers has a control group that offers different tasks and always at the lowest level of difficulty in comparison to the other group. |
| Depression Cognitive Research | We use the information we collect to improve and personalize the Services and to develop new ones. For example, we use the information to troubleshoot and protect against errors; perform data analysis and testing; conduct research and surveys; and develop new features and Services.  A parent, educator, health professional, or researcher may grant you access to the Services and access your personal data, such as your cognitive activity and results. We ask for your explicit consent before sharing your data with such person and we provide you with privacy preferences in account settings and other tools to enable or disable this sharing feature.  Information you have shared with others (e.g. with a parent, educator, health professional, researcher, or some of our partners) will remain visible after you closed your account or deleted the information from your own profile or mailbox, and we do not control data that other users copied out of our Services. Community features content (e.g. challenges) associated with closed accounts will show an anonymous user as the source.  From Website: Who is it for? CogniFit's platform for researchers helps the process of collecting,  managing, and comparing cognitive data within the scientific study. In addition, researchers will have a "control" cognitive training group. Unlike other groups, control group participants will perform different tasks at the lowest level of difficulty, which allows control of certain variables and more consistent research |
| DNA Fit | Self-Reported Information may be converted into Anonymised Information and used in approved Prenetics Research which is subject to separate consent.   RESEARCH AND DEVELOPMENT:  We may Process your Information if you have provided prior, express and voluntary consent for your Biomarker Information and Self-Reported Information to be used in any Prenetics product developent and R&D. This Processing may include sharing your Information with contracted suppliers for Purposes of Prenetics Research and R&D only. We assure you that your information is not sold to any third parties for any other purposes.   For any Prenetics Research you will be contacted beforehand to opt in as a participant for any studies we hope to perform. We will obtain your consent before authorising any scientific publications that includes your Information, even if only Anonymised Information, and for any such scientific publication, this will be subject to full IRB (Institutional Review Board) approval. |
| Dyscalculia Cognitive Research | We use the information we collect to improve and personalize the Services and to develop new ones. For example, we use the information to troubleshoot and protect against errors; perform data analysis and testing; conduct research and surveys; and develop new features and Services.  A parent, educator, health professional, or researcher may grant you access to the Services and access your personal data, such as your cognitive activity and results. We ask for your explicit consent before sharing your data with such person and we provide you with privacy preferences in account settings and other tools to enable or disable this sharing feature.  Information you have shared with others (e.g. with a parent, educator, health professional, researcher, or some of our partners) will remain visible after you closed your account or deleted the information from your own profile or mailbox, and we do not control data that other users copied out of our Services. Community features content (e.g. challenges) associated with closed accounts will show an anonymous user as the source.  From Website: Who is it for?  CogniFit's Cognitive Assessment for Dyscalculia Patients (CAB-DC) helps to measure cognitive abilities related to this disorder in participants in our scientific research. The test methodology allows us to obtain a large amount of data in a comfortable and rigorous way. |
| Dyslexia Cognitive Research | We use the information we collect to improve and personalize the Services and to develop new ones. For example, we use the information to troubleshoot and protect against errors; perform data analysis and testing; conduct research and surveys; and develop new features and Services.  A parent, educator, health professional, or researcher may grant you access to the Services and access your personal data, such as your cognitive activity and results. We ask for your explicit consent before sharing your data with such person and we provide you with privacy preferences in account settings and other tools to enable or disable this sharing feature.  Information you have shared with others (e.g. with a parent, educator, health professional, researcher, or some of our partners) will remain visible after you closed your account or deleted the information from your own profile or mailbox, and we do not control data that other users copied out of our Services. Community features content (e.g. challenges) associated with closed accounts will show an anonymous user as the source.  From Website Who is it for? CogniFit's Cognitive Assessment for Dyslexia Patients (CAB-DX) allows us to evaluate in a simple and accurate way the cognitive abilities related to this reading learning disorder. The way the test is applied makes it convenient for use in scientific research. |
| Fibromyalgia - Research | We use the information we collect to improve and personalize the Services and to develop new ones. For example, we use the information to troubleshoot and protect against errors; perform data analysis and testing; conduct research and surveys; and develop new features and Services.  A parent, educator, health professional, or researcher may grant you access to the Services and access your personal data, such as your cognitive activity and results. We ask for your explicit consent before sharing your data with such person and we provide you with privacy preferences in account settings and other tools to enable or disable this sharing feature.  Information you have shared with others (e.g. with a parent, educator, health professional, researcher, or some of our partners) will remain visible after you closed your account or deleted the information from your own profile or mailbox, and we do not control data that other users copied out of our Services. Community features content (e.g. challenges) associated with closed accounts will show an anonymous user as the source. From Website CogniFit's Cognitive Assessment for Fibromyalgia Patients (CAB-FB) allows for accurate and comfortable testing of a large number of cognitive abilities and to account for certain symptoms. CogniFit's technology takes into account thousands of variables during assessment activities to obtain reliable data about a participant's cognitive state. |
| Google Fit | Health Research Applications and Web Services must confirm the eligibility of a participant before obtaining informed consent and requesting permission to access participant’s data.  Transparent and Accurate Notice and Control for Health Research After confirmation of eligibility, you must comply with the Google Fit Developer and User Data Policy’s Transparent and Accurate Notice and Control section.  In addition, Health Research Applications or Web Services must provide a disclosure via a dialog box or incremental dialogue boxes that include/s:  The nature, purpose, and duration of the research; The risks and benefits to the participant; The privacy, security, and data handling measures in place to protect the data; The point of contact for any questions; The retention period(s) for data collected for the study; How to withdraw from the study; How to delete one’s data from the study throughout the lifecycle of the study, including whether the study permits deletion after the data becomes accessible to the public; and, Any other relevant documents or information required by your IRB/EC.  You must also provide an option for the participant to save, store, or email the above information, the Informed Consent documents, and any other documents required by the IRB/EC. Each participant must sign and submit the required disclosures and consents prior to participation in the study. A copy of all signed documents must be sent to the participant.  Research website (instructions on how to conduct research): https://developers.google.com/fit/conduct-health-research |
| Happiness Project- Play Games for Science | What's this research for? We want to know how simple cognitive traits vary across large groups of people. We also want to find people with interesting groups of traits who might participate in further research. Above all, we want you to enjoy the games, and to find out a bit more about what psychologists do to investigate the workings of the human brain.  What will I do? You will provide some basic demographic information and confirm some settings in order to sign up. After that, you’re free to play the games, and we’ll get useful data each time you play. We would also like to gather some additional information, for example how often you use your phone and how active you are during the day. This information is collected passively by your phone’s sensors.  Do I have to take part in the research to use the app? If you don’t want to take part in the research, go to the Options screen and change ‘Participate in the experiment’ to ‘Off’. Your use of the app will be unaffected. If you change your mind, you change the setting back to ‘On’ at any time.   Is it easy to get out of? Taking part is completely voluntary. You can change your preference in the Options menu at any time and without giving a reason. You can decide not to send us data from the games or passive data from your device’s sensors. You can withdraw completely from the experiment at any time in the Options menu and this will delete all of your data from our data store. Or you can just delete this app from your device at any time (although once you’ve done this, you won’t be able to delete your data from the server). We won’t be able to remove your data from analyses that have already been completed or from data that has already been made available for further research.   From Website Our goal is to develop mathematical equations that explain how humans make decisions, to describe the factors that determine feelings like happiness, and to understand the relationship between happiness and the decisions we make.  By playing the games and completing the surveys in the app, you help us study the causes of happiness. This allows us to better understand how brain processes are linked to mental health problems like anxiety and depression, which may help to find new treatments. |
| Healthy Minds Program | If the App is provided and used in connection with any educational program in which you are participating, we may provide your educational institution with personally identifiable information regarding your usage of the App.  If the App is used in connection with a research study in which you are participating, our privacy practices with respect to that research study will be provided to you separately and at that time.  We may also share de-identified and aggregated data with third parties for research, analytics, or other purposes allowed by applicable law.  From Website  Healthy Minds Program App Research  Educator Well-Being During the Covid-19 Pandemic:  In this randomized controlled trial, Wisconsin educators will either receive four weeks of the Healthy Minds Program app or be assigned to a control condition where access to the program will be provided after data collection. Read more here.  Healthy Minds Program Promoting Resilience During the Covid-19 Pandemic:  This study expands on a previous randomized controlled trial using the Healthy Minds Program as an intervention to improve well-being. Read more here.  The Student Flourishing Initiative:  Working in partnership with other academic institutions, the Center for Healthy Minds is creating and studying the impact of a well-being curriculum for college freshman, which includes use of the Healthy Minds Program App. Read more here.  App-Based Intervention to Decrease Firefighter Psychological Distress and Burnout:  This is a pilot study with a sample of firefighters using the Healthy Minds Program app as the intervention. Read more here. |
| Hevy Gym Log Workout | N/A |
| Huawei Health | N/A |
| InsideTracker | For scientific research: We may include your anonymized blood test information and self-reported information in disclosures to third parties for the purpose of research or other applications, but no identifying information will be shared without your prior knowledge and consent. InsideTracker research is intended to advance wellness and nutrition knowledge and to create, commercialize, or undertake activities toward the practical applications of this learning to the improvement of health care. Our research partners may include commercial or non-profit organizations that conduct or support medical research or conduct or support the development of drugs or devices to diagnose, predict, or treat health conditions. We may ask permission to use your de-identified Genetic Information for research that we hope to publish in scientific publications. If you choose to give this permission, you will agree to a Research Consent document. The Research Consent document is optional and voluntary. You may use the website or services without agreeing to the Research Consent document.  Blood biomarker data is data related to your blood test results from tests done by InsideTracker laboratory partners. You may also upload existing blood test results from tests ordered by your doctor or insurance company. We may use your blood biomarker data in a de-identified, aggregated way for InsideTracker research.  Genetic (DNA) information is data related to your genotype for a specific set of genes related to healthy aging, nutrition, weight, sleep and physical activity. InsideTracker will receive your genetic information from our partner Helix when you buy the GoalGetter product. [..] InsideTracker analyzes your DNA data using an algorithm that determines your genetic potential for certain traits. InsideTracker may use your aggregated, de-identified genetic data for research and development to improve future products. For research that we hope to publish in scientific publications, we will request separate permission through a Research Consent document to use your de-identified Genetic Information. Any Research Consent is optional and voluntary. You will not be required to agree to a Research Consent document in order to use the Platform or Services.  Self-Reported Information includes information you provide in the InsideTracker questionnaire or in any other website surveys or forms, such as sex, body weight, height, diet, etc. We may use your Self-Reported Information in a de-identified way for InsideTracker research.  if you close your account after you have separately agreed to the Research Consent document, any genetic information that you have previously given consent to use in de-identified form will not be removed from ongoing or completed studies.  From Website (consent form): https://www.insidetracker.com/research-consent/ https://www.insidetracker.com/product-consent/ |
| Insomnia – Cognitive Research | We use the information we collect to improve and personalize the Services and to develop new ones. For example, we use the information to troubleshoot and protect against errors; perform data analysis and testing; conduct research and surveys; and develop new features and Services.  A parent, educator, health professional, or researcher may grant you access to the Services and access your personal data, such as your cognitive activity and results. We ask for your explicit consent before sharing your data with such person and we provide you with privacy preferences in account settings and other tools to enable or disable this sharing feature.  Information you have shared with others (e.g. with a parent, educator, health professional, researcher, or some of our partners) will remain visible after you closed your account or deleted the information from your own profile or mailbox, and we do not control data that other users copied out of our Services. Community features content (e.g. challenges) associated with closed accounts will show an anonymous user as the source.  From Website:  If we want to study the effect of cognitive stimulation in adults with insomnia, CogniFit's platform for researchers gives us the possibility to conveniently manage and compare the data of the participants in our study. In addition, the CogniFit Platform for Researchers has a control group that offers different tasks and always at the lowest level of difficulty in comparison to the other group. |
| Medisafe Pill & Med Reminder | WITH WHOM DO WE SHARE YOUR PERSONAL INFORMATION Research Partners- we may share your Personal Information with third parties, such as research institutes, healthcare systems and healthcare providers. They may associate it with other information that they have about you, for improved healthcare, research purposes and the improvement of our Service.  Third Party Health Apps – we participate in Apple HealthKit, Samsung S-Health and Google Fit frameworks. We will use information that you consent that we receive from those third party health apps for medical research purposes, internally in order to improve our products and services and in the form of aggregated and/or analytical data as described in the Use of Aggregated Data section of the Privacy Policy, and may transfer this data to third parties for such purposes. We may provide users with the ability to share their Medisafe data with other authorized services on their device after receiving their consent for the same.   USE OF AGGREGATED DATA We may de-identify and aggregate Personal Information; aggregated data will not contain any information that could be used to contact or identify you. We may analyze and/or combine all information we receive, including Health Information and information regarding your use of the Service, with information from other users to create aggregated data that may be disclosed to and utilized by us, our partners and by third parties without restriction, on commercial terms that we can determine in our sole discretion, for purposes such as: content marketing, research purposes, in order to understand behavior patterns, in order to increase adherence to medication regimens, marketing strategies and for entering into commercial contracts in order to provide our users with the Service. As an exception to the above, we will not include data received via Apple HealthKit in the aggregated data we share with and/or sell to third parties or for marketing purposes. |
| MyTherapy Pill Reminder | From Website: We believe that MyTherapy can improve healthcare overall. We work together with reputable research partners such as Charité Berlin, who support us with scientific analyses of aggregated user data. Your data privacy is not compromised in this process and data cannot be traced back to any specific user. |
| NeuroPsy Research | Research Data For each study, that a user participates in, the NeuroPsyResearch app collects research data that is relevant to the study’s research question. These data may include, but is not limited to, for example, the time of day the study was conducted, and measures such as reaction times and scores.  For each user account created in the app, a randomly generated character string is created as part of the research data so that the data can be attributed to an anonymously participating person. The name chosen for the user account is not part of the research data and will not be transmitted when uploading the data. The collected research data will not reveal the identity of the participating user.  It is the responsibility of the participants to protect their user account and the device against unauthorized access.  Usage of Anonymized Data The results and collected research data from any study in the NeuroPsyResearch app will be published as a scientific publication. This is done in anonymized form, i.e. without the research data on its own being able to identify a specific person. The voluntary information about age group and gender of participants are used to assess the representativeness of the research data. The fully anonymized data from these studies are made available on the Internet as open data under the CC-BY-SA license. This means that the research data can also be used for any purposes other than the studies they were originally collected for, including commercial purposes.  The collected research data will be processed to perform statistical analysis for scientific inquiries in a semi-automatic fashion. A link to the web-interface for analyzing the research data is provided within the NeuroPsyResearch app.  After completing a study, the data will possibly be stored and published in a national or international data archive. These studies will thus follow the recommendations of the German Research Foundation (DFG) and the German Society for Psychology (DGPs) for quality assurance in research. |
| Parkinson’s Cognitive Research | We use the information we collect to improve and personalize the Services and to develop new ones. For example, we use the information to troubleshoot and protect against errors; perform data analysis and testing; conduct research and surveys; and develop new features and Services.  A parent, educator, health professional, or researcher may grant you access to the Services and access your personal data, such as your cognitive activity and results. We ask for your explicit consent before sharing your data with such person and we provide you with privacy preferences in account settings and other tools to enable or disable this sharing feature.  Information you have shared with others (e.g. with a parent, educator, health professional, researcher, or some of our partners) will remain visible after you closed your account or deleted the information from your own profile or mailbox, and we do not control data that other users copied out of our Services. Community features content (e.g. challenges) associated with closed accounts will show an anonymous user as the source.  From Website: This brain training program allows researchers and scientists from around the world to carry out cognitive interventions and study in depth the neuropsychological characteristics of Parkinson's disease. CogniFit saves time by helping in the collection, management, and data study analysis. In addition, the CogniFit Researcher tool has a control group that offers different tasks than the "intervention group", and keeps difficulty low. This makes it easy to create experimental designs. |
| Renpho Health | We may use aggregated, deidentified data from the above sources for statistical analysis, research, commercial, and other purposes. Aggregate, deidentified data is not “personal data,” “personal information,” or “your” data or information under this Privacy Policy or under law. We may share aggregate, deidentified data about Services users with third parties to help us understand our user demographic, including user demographic interests, habits, and usage patterns for certain of our Services so that we may market our products more effectively. |
| Smart Omix by Sharecare | N/A  From Website (for researchers):  Research info page (for researchers): https://www.sharecare.com/research/smartomix |
| Symptom & Mood Tracker | From Website: Start A Health Experiment Today Discover how different habits & treatments impact your health. Share your progress and find out about upcoming experiments on the Bearable Discord Server (accessible via your profile page in the app) |
| Symptomate - Symptom checker | AUTOMATED DECISION MAKING You use the Application thanks to our intelligent algorithm, which carefully analyzes your answers given in the interview, so you can learn the possible causes of your symptoms. This analysis is done automatically, based on the information you provide and, as a result, the tool can suggest your potential health condition. This process is referred to as "profiling" and its use if it concerns personal data is regulated by law (Article 22 of GDPR). This analysis is the essence of the service and must be carried out for you to receive an interview result. In addition, we also analyze the way in which you use the Application - thanks to this we can improve the quality of our solution and make the medical device not only safer but also more friendly and effective. The data and results of the analyses performed are not used for marketing purposes.  The analysis performed, although it may be considered as profiling in nature, does not produce any legal effects on you or similarly significantly affect you. |
