# Supplementary material for: Mapping the Apps: Ethical and Legal Issues with Crowdsourced Smartphone Data using mHealth Applications
Source: Asian Bioeth Rev. 2024 Jun 18;16(3):437–70. doi: 10.1007/s41649-024-00296-3 (PMC11250705; doi:10.1007/s41649-024-00296-3)
Supplement: Supplementary file 3 — (DOCX 23.0 kb) [file 41649_2024_296_MOESM3_ESM.docx]

| Appendix 2: Mentions of Commercialization | |
| --- | --- |
| App | Mentions of Commercialization |
| 23andMe - DNA Testing | N/A |
| Ada - Check your Health | We will never share your personal health information with advertisers or third parties.  We restrict access to your personal data to the persons who need to use it for the relevant purpose(s), always in compliance with the integrity and confidentiality principle.  From Website: No personal or health data has been sold to third parties or shared for any commercial purpose. Ada does not share your data with anyone unless you explicitly tell us to do so. We are funded through private investment, as well as commercial relationships with several health systems, insurers, and life science companies to provide services for their users. Ada also receives funding from charitable foundations (including The Rockefeller Foundation; The Bill and Melinda Gates Foundation; Fondation Botnar) to improve access to care, practitioner quality and universal coverage in low and middle-income countries. Ada has also received grants from government programs (inc. World Health Organization; World Economic Forum, European Commission). |
| Ancestry: Family History & DNA | See data sharing section Refer to the Informed Consent to Research for information on what is disclosed with research partners, which may include: -Identifiers; -Categories of personal information described in Section 1798.80(e) of the California Civil Code; -Characteristics of protected classes under applicable state or federal law; -Commercial information; -Machine-readable DNA data; -Internet usage information; -Sensory data; and, Inferences.  We work with third-party partners for analytics and advertising purposes. By collecting and sharing certain Personal Information, these vendors help us to better personalize ads to match your interests. They also help us to measure the effectiveness of ad campaigns and are used to serve you with advertising that is more relevant to you. |
| Apple Research | Apple does not track its customers over time and across third party websites to provide targeted advertising and therefore does not respond to “Do Not Track” (DNT) signals. |
| CovidWatcher | N/A |
| DNA ID, Inc. | N/A |
| DnaNudge | The processing of your personal data is lawful based on the following:   Our legitimate interests in (among other things) operating and administering the Service, conducting commercial research, improving and maintaining our Service, personalising and tailoring content made available to you through the Service, protecting the security or integrity of our databases or the Service, protecting our business or reputation, taking precautions against legal liability, protecting and defending our rights or property, or for resolving disputes, investigating and attending to inquiries or complaints with respect to your use of the Service. |
| FLARe Research | N/A |
| Gene Doe* | We may share Your information with Our business partners to offer You certain products, services or promotions. |
| GenePlanet | If your Personal Data is processed for direct marketing purposes, including profiling, you can object to such processing at any time, and your Personal Data will no longer be processed for this purpose. |
| Mass Science | N/A |
| My Toolbox Genomics | We use registration information for market research, and for advertising purposes in compliance with applicable laws.   Disclosure of Information: To help us connect with new audiences who have similar interests and demographics as our current customers, we engage marketing affiliates, partners, and service providers. Some of these third parties may process Personal Information (but no Genetic Information) on our behalf for such marketing purposes, although we take steps to avoid or minimize the use of Personal Information for marketing—through processes such as aggregation (using information to create statistics that do not identify any particular individual) and hashing (converting information on our end into a cryptographic hash, then providing the hash to a service provider who is then only able to correlate our data with data on individuals it already has).  In some cases, these third parties process Personal Information on our behalf. From time to time, we provide a limited set of information to service providers who help us develop and aggregate information on our customers’ demographics, which is then used to display advertisements to other individuals likely to have similar interests. We may also receive marketing leads from third parties based on other data they have collected. We and our service providers take steps to minimize the use of any Personal Information (if Personal Information is needed at all), including, where possible, and transferring a hashed set of data that can only be used to correlate to other data that the service provider already has |
| MyGeneRank | N/A |
| OH Data Port | N/A |
| Pattern Health | To engage in marketing activities, we also collect information from your publicly available social media profiles, interests and preferences including LinkedIn. We process such information to better understand you, to maintain and improve the accuracy of the information we store about you, and to better promote or optimize our Services. In addition, we may also use this information to deliver targeted advertising and marketing to you, where permitted by applicable law and in accordance with your marketing preferences. |
| Project Serotonin | N/A |
| StuffThatWorks | We will not share your personal information without your explicit consent, with our valued partners, including but not limited to those in the medical, pharmaceutical and biotechnology industries, academic institutions, and government agencies and regulatory bodies, including regulatory bodies such as the American CDC and FDA, or other national and international bodies, as applicable and as necessary. We may share only de-identified and/or aggregated information with Partners, in order to conduct scientific, and/or medical research, as part of our Service. When disclosing information to our Partners or otherwise selling user information for scientific or market research purposes, we make sure to anonymize and/or remove all Personal Information or other personally-identifying indicators in the data (de-identification) to minimize the possibility of accidental member identification.  From Website: Q. Will you sell my personal information?  Absolutely not. We will never sell your personal information to any third parties. |
| Urban Mind | N/A |
| Withings Health mate | We collect your consent to process personal data for: marketing communications.   Marketing Communication  PROCESSED DATA: E-mail address LEGAL BASIS: Consent to this processing when creating your account RETENTION PERIOD: Data is retained as long as your account is active or when you no longer wish to be notified |
| ActiveDay - Activity Study | Some content or applications, including advertisements, on the Sites are served by third-parties, including advertisers, ad networks and servers, content providers, and application providers. These third parties may use cookies alone or in conjunction with web beacons or other tracking technologies to collect information about you when you use our Sites. The information they collect may be associated with your personal information or they may collect information, including personal information, about your online activities over time and across different Sites and other online services. They may use this information to provide you with interest-based (behavioral) advertising or other targeted content.  We do not control these third parties’ tracking technologies or how they may be used. If you have any questions about an advertisement or other targeted content, you should contact the responsible provider directly. For information about how you can opt out of receiving targeted advertising from many providers, see Choices About How We Use and Disclose Your Information.  We may also use your information to contact you about our own and third-parties’ goods and services that may be of interest to you. If you do not want us to use your information in this way, please adjust your user preferences in your account profile.  We may use the information we have collected from you to enable us to display advertisements to our advertisers’ target audiences. Even though we do not disclose your personal information for these purposes without your consent, if you click on or otherwise interact with an advertisement, the advertiser may assume that you meet its target criteria. |
| ADHD - Cognitive Research | If you click on an external link or ad on our Services, that advertiser or website operator might figure out that you came from CogniFit, along with other information associated with the ad you clicked such as characteristics of the audience it was intended to reach. They may also collect other personal data from you, such as cookie identifiers or your IP address.  We work with partners who provide us with analytics and advertising services. This includes helping us understand how users interact with the Services, serving ads on our behalf across the internet, and measuring the performance of those ads. These companies may use cookies and similar technologies to collect information about your interactions with the Services and other websites and applications. To learn more and about your privacy choices, please read our Cookie Use statement.  We never sell the personal information of our users. We do work with partners who provide us with advertising services as described in the Analytics and Advertising Services Provided by Others section. To learn more about how these partners collect data and your options for controlling the use of your information for interest-based advertising, please read our Cookie Use statement. |
| Andaman7 Private Health Record | N/A |
| Atlas Health | Behavioral Remarketing Atlas Health Europe Limited uses remarketing services to advertise on third party websites to you after you visited our Service. We and our third-party vendors use cookies to inform, optimize and serve ads based on your past visits to our Service. [List of marketers] |
| Behavidence Research App | We use cookies for a number of purposes, as briefly explained below:  Marketing. Cookies that are used to enable better tailoring of online ads and marketing campaigns to you based on the websites you visited and content you viewed.  We will process your Website Analytics Information with the assistance of our service providers who assist us with the internal operations of the Website. These companies are authorized to use your statistical information, which does not contain any identifying details about you, in this context only as necessary to provide these services to us and not for their own promotional purposes. |
| Better- Rewards for Health | N/A but applicable with data sharing |
| Chemo Brain Cognitive Research | If you click on an external link or ad on our Services, that advertiser or website operator might figure out that you came from CogniFit, along with other information associated with the ad you clicked such as characteristics of the audience it was intended to reach. They may also collect other personal data from you, such as cookie identifiers or your IP address.  We work with partners who provide us with analytics and advertising services. This includes helping us understand how users interact with the Services, serving ads on our behalf across the internet, and measuring the performance of those ads. These companies may use cookies and similar technologies to collect information about your interactions with the Services and other websites and applications. To learn more and about your privacy choices, please read our Cookie Use statement.  We never sell the personal information of our users. We do work with partners who provide us with advertising services as described in the Analytics and Advertising Services Provided by Others section. To learn more about how these partners collect data and your options for controlling the use of your information for interest-based advertising, please read our Cookie Use statement. |
| Depression Cognitive Research | If you click on an external link or ad on our Services, that advertiser or website operator might figure out that you came from CogniFit, along with other information associated with the ad you clicked such as characteristics of the audience it was intended to reach. They may also collect other personal data from you, such as cookie identifiers or your IP address.  We work with partners who provide us with analytics and advertising services. This includes helping us understand how users interact with the Services, serving ads on our behalf across the internet, and measuring the performance of those ads. These companies may use cookies and similar technologies to collect information about your interactions with the Services and other websites and applications. To learn more and about your privacy choices, please read our Cookie Use statement.  We never sell the personal information of our users. We do work with partners who provide us with advertising services as described in the Analytics and Advertising Services Provided by Others section. To learn more about how these partners collect data and your options for controlling the use of your information for interest-based advertising, please read our Cookie Use statement. |
| DNA Fit | MARKETING AND ADVERTISING:   From time to time we may send you communications, across our brands, within the Prenetics group, about new services available to you, discounts, events, invite you to participate in relevant Prenetics Research or obtain testimonials for promotional purposes.  We may also direct advertising to you via third party sites including social media. We will only send marketing material to you in accordance with this Privacy Policy, where we have a legitimate interest to do so, where you have opted-in to such communications or as determined by your web browser/cookie settings. You may change your marketing preferences at any time via your account settings. |
| Dyscalculia Cognitive Research | If you click on an external link or ad on our Services, that advertiser or website operator might figure out that you came from CogniFit, along with other information associated with the ad you clicked such as characteristics of the audience it was intended to reach. They may also collect other personal data from you, such as cookie identifiers or your IP address.  We work with partners who provide us with analytics and advertising services. This includes helping us understand how users interact with the Services, serving ads on our behalf across the internet, and measuring the performance of those ads. These companies may use cookies and similar technologies to collect information about your interactions with the Services and other websites and applications. To learn more and about your privacy choices, please read our Cookie Use statement.  We never sell the personal information of our users. We do work with partners who provide us with advertising services as described in the Analytics and Advertising Services Provided by Others section. To learn more about how these partners collect data and your options for controlling the use of your information for interest-based advertising, please read our Cookie Use statement. |
| Dyslexia Cognitive Research | If you click on an external link or ad on our Services, that advertiser or website operator might figure out that you came from CogniFit, along with other information associated with the ad you clicked such as characteristics of the audience it was intended to reach. They may also collect other personal data from you, such as cookie identifiers or your IP address.  We work with partners who provide us with analytics and advertising services. This includes helping us understand how users interact with the Services, serving ads on our behalf across the internet, and measuring the performance of those ads. These companies may use cookies and similar technologies to collect information about your interactions with the Services and other websites and applications. To learn more and about your privacy choices, please read our Cookie Use statement.  We never sell the personal information of our users. We do work with partners who provide us with advertising services as described in the Analytics and Advertising Services Provided by Others section. To learn more about how these partners collect data and your options for controlling the use of your information for interest-based advertising, please read our Cookie Use statement. |
| Fibromyalgia - Research | If you click on an external link or ad on our Services, that advertiser or website operator might figure out that you came from CogniFit, along with other information associated with the ad you clicked such as characteristics of the audience it was intended to reach. They may also collect other personal data from you, such as cookie identifiers or your IP address.  We work with partners who provide us with analytics and advertising services. This includes helping us understand how users interact with the Services, serving ads on our behalf across the internet, and measuring the performance of those ads. These companies may use cookies and similar technologies to collect information about your interactions with the Services and other websites and applications. To learn more and about your privacy choices, please read our Cookie Use statement.  We never sell the personal information of our users. We do work with partners who provide us with advertising services as described in the Analytics and Advertising Services Provided by Others section. To learn more about how these partners collect data and your options for controlling the use of your information for interest-based advertising, please read our Cookie Use statement. |
| Google Fit | All other transfers, uses, or sale of participant data is expressly prohibited, including:  -Transferring, selling, or using participant data for serving ads, including contextual, retargeting, personalized, or interest-based advertising. -Transferring or selling participant data to third parties like advertising platforms, data brokers, or any other information resellers. |
| Happiness Project- Play Games for Science | Not clear but: We will never sell your data to any third party. We may make anonymous data available for further research by other parties such as academic researchers at other institutions. |
| Healthy Minds Program | We may use your personal information to contact you about our products and services, to send you information about new offerings, and/or to improve the quality of our Website or App or our product and service offerings and to help us better serve you (whether or not through our support services).  We may also use that information to deliver you or contact you about requested products/services or information. In addition, we may use your personal information to provide important information about products or services that you have or are using, including security risks and updates. We may send you information about new products and services that may be of relevant interest to you. If you do not want to receive such marketing communications from us in the future, we will provide an Opt-out link or you can let us know by e-mailing or mailing us at the below respective addresses and telling us the name and addresses that you would like removed for those communications. |
| Hevy Gym Log Workout | Google may use the Data collected to contextualize and personalize the ads of its own advertising network. Users must know that, however, should their Personal Data be processed for direct marketing purposes, they can object to that processing at any time, free of charge and without providing any justification. Where the User objects to processing for direct marketing purposes, the Personal Data will no longer be processed for such purposes. To learn whether the Owner is processing Personal Data for direct marketing purposes, Users may refer to the relevant sections of this document. |
| Huawei Health | We shall use your device information (such as device country and language) and app usage data (such as which banners you view and which buttons you click in the app) for sales promotion and marketing purposes, including creating aggregated target groups for marketing. Knowing customers' preferences enables us to target our offers and offer products and services that better meet the needs and expectations of our customers. You can object to such data processing as described in Section 5.6 below. |
| InsideTracker | You may find advertising or other content on the Platform that link to the websites and services of our partners, suppliers, advertisers, sponsors, licensors, and other third parties. We do not control the content or links that appear on these websites. We are not responsible for the practices employed by websites linked to or from the Platform.  We do not use your blood test results or DNA data for marketing. Of course, you are free to opt out from hearing from us by any of these channels at any time.  We may disclose Personal Information about you in the ways described below and/or to the third-parties mentioned below: Affiliates. To our parents, affiliates, joint ventures, or promotion partners, for their use in a manner consistent with the purposes described in this Privacy Policy. |
| Insomnia - Cognitive Research | If you click on an external link or ad on our Services, that advertiser or website operator might figure out that you came from CogniFit, along with other information associated with the ad you clicked such as characteristics of the audience it was intended to reach. They may also collect other personal data from you, such as cookie identifiers or your IP address.  We work with partners who provide us with analytics and advertising services. This includes helping us understand how users interact with the Services, serving ads on our behalf across the internet, and measuring the performance of those ads. These companies may use cookies and similar technologies to collect information about your interactions with the Services and other websites and applications. To learn more and about your privacy choices, please read our Cookie Use statement.  We never sell the personal information of our users. We do work with partners who provide us with advertising services as described in the Analytics and Advertising Services Provided by Others section. To learn more about how these partners collect data and your options for controlling the use of your information for interest-based advertising, please read our Cookie Use statement. |
| Medisafe Pill & Med Reminder | We will not sell any Personal Information nor will we use this data for advertising purposes. Please note that when you share information with third party apps, such information will be subject to their privacy policy, over which we have no control.   We may analyze and/or combine all information we receive, including Health Information and information regarding your use of the Service, with information from other users to create aggregated data that may be disclosed to and utilized by us, our partners and by third parties without restriction, on commercial terms that we can determine in our sole discretion, for purposes such as: content marketing, research purposes, in order to understand behavior patterns, in order to increase adherence to medication regimens, marketing strategies and for entering into commercial contracts in order to provide our users with the Service  HOW DO WE USE THE INFORMATION WE COLLECT Marketing purposes – subject to your marketing preferences, we may use your Personal Information (such as your email address or phone number) to communicate with you. We may also send you promotional material concerning our services or our partners’ services (which we believe may interest you), including but not limited to, by building an automated profile based on your Personal Information, for marketing purposes. Opt-out of receiving marketing materials – If you do not want us to use or share your Personal Information for marketing purposes, you may opt-out by contacting us in accordance with this Privacy Policy, by clicking the “unsubscribe” button included in every marketing communication you receive from us, or with respect to tracking technologies, by using the cookie preferences menu displayed on our Site. Please note that even if you opt-out, we may still use and share your Personal Information with third parties for non-marketing purposes (for example to fulfill your requests, communicate with you and respond to your inquiries, etc.). |
| MyTherapy Pill Reminder | You can import health data from other applications, such as step count from a fitness tracker via Apple HealthKit or Google Fit and add them to your treatment documentation, if you technically allow Apple and Google to do so. We use Apple HealthKit or Google Fit data only for this purpose.  Apple HealthKit: We never use your Apple HealthKit data for advertising and similar services or for use-based data mining. We do not disclose information gained through Apple HealthKit to any third party or sell it to advertising platforms, data brokers, or information resellers.  Google Fit: We do not use the content accessed from Google Fit in connection with any advertising, sponsorship or promotions, we do not share or sell that content to any data broker or information reseller, and we do not permit our users to do any of the above.  We use AppsFlyer to receive analytics data about your MyTherapy usage linked with MyTherapy marketing campaigns. We collect data about the technical device (device type, operating system and version, screen resolution, etc.), the anonymized IP address and internet service provider, pages visited in the app and items clicked by the user, mobile advertising ID. Place of processing are United States of America & European Union. For more information, see https://www.appsflyer.com/services-privacy-policy/. |
| NeuroPsy Research | The fully anonymized data from these studies are made available on the Internet as open data under the CC-BY-SA license. This means that the research data can also be used for any purposes other than the studies they were originally collected for, including commercial purposes. |
| Parkinson's Cognitive Research | If you click on an external link or ad on our Services, that advertiser or website operator might figure out that you came from CogniFit, along with other information associated with the ad you clicked such as characteristics of the audience it was intended to reach. They may also collect other personal data from you, such as cookie identifiers or your IP address.  We work with partners who provide us with analytics and advertising services. This includes helping us understand how users interact with the Services, serving ads on our behalf across the internet, and measuring the performance of those ads. These companies may use cookies and similar technologies to collect information about your interactions with the Services and other websites and applications. To learn more and about your privacy choices, please read our Cookie Use statement.  We never sell the personal information of our users. We do work with partners who provide us with advertising services as described in the Analytics and Advertising Services Provided by Others section. To learn more about how these partners collect data and your options for controlling the use of your information for interest-based advertising, please read our Cookie Use statement. |
| Renpho Health | We may use aggregated, deidentified data from the above sources for statistical analysis, research, commercial, and other purposes. Aggregate, deidentified data is not “personal data,” “personal information,” or “your” data or information under this Privacy Policy or under law. We may share aggregate, deidentified data about Services users with third parties to help us understand our user demographic, including user demographic interests, habits, and usage patterns for certain of our Services so that we may market our products more effectively.  INTEREST-BASED ADVERTISING: We may use third-party advertising companies that use tracking technologies to serve our advertisements across the Internet. These companies may collect data about your visits to the Services and other websites and your interaction with our advertising and other communications. These advertising companies serve ads on behalf of us and others on non-affiliated sites, and some of those ads may be personalized, meaning that they are intended to be relevant to you based on data collected about your visits to the Services and elsewhere over time. Other companies may also use such technology to advertise on our Services.  In particular, we may share certain information with Facebook that allows us to create Custom or Lookalike Audiences. You may learn more about Facebook Lookalike Audiences here and how to opt out of having your off-Facebook activity sent to Facebook here. We encourage you to review Facebook's Privacy Policy here.  You have the choice to tell us not to collect and use this data, and in some jurisdictions, we will only engage in interest-based advertising if you opt-in. |
| Smart Omix by Sharecare | Advertising. Unless you are receiving Services as part of an Enterprise Program, we may also personalize your experience on our sites and mobile Services by showing you advertisements from Sharecare or our advertising partners that are tailored to your interests. Use our privacy webform to opt out of advertising to the extent it is applicable to you.  Sharecare does not advertise, market, or promote products or services to patients, participants, or practitioners who receive Services as part of an Enterprise Program. In the event your Enterprise Organization, through which you receive Services, instructs us to provide advertising content on this site, Sharecare would first obtain your valid authorization in compliance with applicable data privacy laws. |
| Symptom & Mood Tracker | Bearable uses your personal data, such as your email address, to contact you with messages, emails, and newsletters. These may include push notifications, in-app messages and emails to deliver health content and occasional promotional materials that may be of interest to you.  From Website: We'll never sell your data Companies in the past have resorted to this out of desperation and greed, while our main motivation will always be to help people over making large profits. |
| Symptomate - Symptom checker | Marketing:  Additionally, we take advantage of marketing services in order to analyze your behavior for the purposes of optimizing the functioning of the Website and the App, as well as for the purposes of optimizing our advertising activities. In particular, we take advantage of technologies such as Facebook Pixel, Google Analytics, FullStory, and Amplitude.  In such a case the data is collected and processed for marketing purposes, and the legal grounds for processing is legitimate interest (legal ground under Article 6(1)(f) of GDPR) understood as the desire to reach as many users and customers as possible, to promote our Application and Services and thereby develop our business.  Data will be processed for as long as the services are provided, until an objection is submitted or until a change is made to the settings of your browser or in your Facebook or Google profile.  (They also provide a list of their online marketing services) |
