# Supplementary material for: Mapping the Apps: Ethical and Legal Issues with Crowdsourced Smartphone Data using mHealth Applications
Source: Asian Bioeth Rev. 2024 Jun 18;16(3):437–70. doi: 10.1007/s41649-024-00296-3 (PMC11250705; doi:10.1007/s41649-024-00296-3)
Supplement: Supplementary file 4 — (DOCX 37.0 kb) [file 41649_2024_296_MOESM4_ESM.docx]

| Appendix 3: Mentions of Data Sharing | |
| --- | --- |
| App | Data Sharing Mentions |
| 23andMe - DNA Testing | Who we share with: Service providers: Our service providers and contractors help us provide our Services and act on our behalf to get things done. We implement procedures and maintain contractual terms with each service provider and contractor to protect the confidentiality and security of your Personal Information.  Your sharing choices: You may direct us to share your Personal Information with friends, family members, doctors or other healthcare professionals, and/or any other individuals or entities who may or may not be using our Services, including through third party services such as social networks and third-party apps that connect to our Services. If you share your Personal Information with a third party, they may use your Personal Information differently than we do under this Privacy Statement.   Commonly owned entities, affiliates and change of ownership: If we are involved in a bankruptcy, merger, acquisition, reorganization, or sale of assets, your Personal Information may be accessed, sold or transferred as part of that transaction and this Privacy Statement will apply to your Personal Information as transferred to the new entity.   Third parties related to law, harm, and the public interest: We can’t say it enough – 23andMe will not​ provide information to ​law enforcement​​ unless required by law to comply with a valid court order, subpoena, or search warrant.   Who we DO NOT share with: You can rest assured, we will not voluntarily share your Personal Information with: -Public databases​ -Insurance companies or ​employer​s -Law enforcement, absent a valid court order, subpoena, or search warrant |
| Ada - Check your Health | No data will be shared with any third party in this step without your specific consent.  Your data is only processed internally at Ada and will not be shared with our partners.  Please be aware that information on your use of the branded care options might be shared with our partners in aggregated and anonymous form even when you revoked your consent.  We may share and present the results as summarized statistics to our partners, e.g. in the public health and scientific community, always on an irreversibly anonymized basis.  The processing is necessary for reasons of public interest in the area of public health (Article 9(2)(i) GDPR, Article 22 (1)(1)(c) BDSG)). Our legitimate interest in processing data for these purposes is to support public health progress by protecting against serious cross- border threats to health. You may, for reasons arising from your particular situation, object to such a processing at any time by writing us  From Website:  Ada does not share your personal data with third parties for their own purposes (what is often referred to as selling the data) without your explicit consent. We will ask you whether you would like to share your data with our partners when we believe this is of benefit to you, e.g. to show you available care options based on your report. You do not have to provide consent, and you can revoke your consent at any time.  We may share your personal data with third-party subcontractors that we rely on to assist us in providing Ada services. Transferring data to our service providers is very different to transferring data to third parties for their own purposes. When data is transferred (shared), service providers only have access to your data when deemed necessary by Ada. All service providers are bound by a data protection agreement and will only process data according to our instructions. Read the Privacy Policy (section 3) for complete details about data handling. |
| Ancestry: Family History & DNA | We work with other companies when providing and marketing the Services. As a result, these companies will have access to or otherwise process your data, including some of your Personal Information, in their systems. These companies are subject to contractual obligations governing privacy, data security, and confidentiality consistent with applicable laws.  We may use some Personal Information (for example, demographic information available from third-party sources or from parts of Ancestry (profile, trees, etc.)) to market our products and offers from us or our business partners. This marketing includes advertising based on your interests. Please note: Ancestry does not share your Genetic Information with third-party marketers, insurance companies, or employers, and we will not use your Genetic Information for marketing or personalized advertising without getting your explicit consent. |
| Apple Research | When Apple acts as the study sponsor, it is obligated to put in place a process that could include the review of non-Coded Study Data as needed to comply with law in certain jurisdictions or to fulfill Apple’s obligations as sponsor. Apple hires a third party to fulfill such obligations on its behalf and only Coded Study Data is received on Apple systems. For certain studies, if you experience a technical issue and consent to be transferred to Apple for additional technical support, Apple may have access to certain information that directly identifies you. Other entities involved in the Study, such as the Study principal investigator or other Study Team members, may maintain Study Data in an identifiable form.  Your personal information and Coded Study Data may also be disclosed to the following third parties:  -Government and Regulatory authorities, such as the U.S. Department of Health and Human Services, the Food and Drug Administration -Law enforcement or other third parties pursuant to valid legal process such as a subpoena, litigation or court order.  -Other Approved Study Researchers, if permitted by the Informed Consent certain third-party researchers who are approved may access limited Study Data. The categories of approved study researchers, the type of Study Data they may have access to, and the purposes that they may use the data for will be described in more detail in the Informed Consent. -Others, if we determine that disclosure is reasonably necessary to enforce our terms and conditions or protect our operations or users. We disclose information as described in the Informed Consent. Additionally, in the event of a reorganization, merger, or sale we may transfer any and all personal information we collect to the relevant third party. |
| CovidWatcher | The following individuals and/or agencies will be able to look at and copy your research records - The investigator, Columbia University Medical Center study staff and other medical professionals who may be evaluating the study - Authorities from Columbia University, including the Institutional Review Board ('IRB') - The Office of Human Research Protections ('OHRP')  Your data may be shared as part of research collaboration with other research teams. However, only the ‘de-identified’ part of your data will be shared. We will not share any of your data with any non-research third party. |
| DNA ID, Inc. | Your data is share only on a double-opt-in with who you choose to share it with. Instead of aggregating your data and selling it in mass amounts, we enable a more transparent data sharing model on the blockchain. Each the researcher, and the contributor will have an opportunity to confirm or deny they want to facilitate a data share.  From Website: Manage what data to share Ensure that you have full control over who's using your health data. With permissions at the project, you can share your gender, ethnicity, height, weight, & other phenotypes in an anonymous, de-identified way. Get paid $$$ for doing good. The more you share, the more your data is worth (don't forget that you're helping accelerate disease research too).  How is my data shared? Your data is share only on a double-opt-in with who you choose to share it with. Instead of aggregating your data and selling it in mass amounts, we enable a more transparent data sharing model on the blockchain. Each the researcher, and the contributor will have an opportunity to confirm or deny they want to facilitate a data share.  What information does DNA ID share with a Researcher? We only share the data that you want to share! Your DNA is the baseline for establishing a share, but remember you can always say no to sharing. |
| DnaNudge | -Data is transferred to our database which is held ‘in the cloud’ on servers operated by a third party service provider (however, the encrypted DNA data – which on its own is meaningless - is held in a form that cannot be linked to the user except through the user’s account); -Related Parties that we use to assist us in delivering the Service (including administration services, technical services relating to the maintenance, servicing and upgrading of the Service hosting and cloud computing services, data migration and analytical services, marketing and customer service, payment processing services, and other outsourced services); -Related Parties that help us to test, monitor, improve and develop the Service; -Related Parties that help us compile, aggregate and analyse personal data in order to produce Anonymised and Aggregated Data that we use, sell and publish; -Related Parties that help us perform analytical studies and research. We will not disclose the results of such analysis or research to third parties or publish it except in the form of Anonymised and Aggregated Data.  If we believe that such disclosure is reasonably necessary to enforce or apply our subscription terms and conditions or to protect our rights, property, the safety or integrity of our services, software or products; To protect the Service against abuse or unauthorised access and to protect the personal data of our Users in general; Where necessary to satisfy a legitimate request or order of a government body, public authority, regulator or enforcement agency, in response to a third-party subpoena etc. |
| FLARe Research | N/A |
| Gene Doe | Disclosure of Your Personal Data Business Transactions If the Company is involved in a merger, acquisition or asset sale, Your Personal Data may be transferred. We will provide notice before Your Personal Data is transferred and becomes subject to a different Privacy Policy.  Law enforcement Under certain circumstances, the Company may be required to disclose Your Personal Data if required to do so by law or in response to valid requests by public authorities (e.g. a court or a government agency). Other legal requirements (etc)  We may share your personal information in the following situations:  -With Service Providers: We may share Your personal information with Service Providers to monitor and analyze the use of our Service, to contact You. -For Business transfers: We may share or transfer Your personal information in connection with, or during negotiations of, any merger, sale of Company assets, financing, or acquisition of all or a portion of our business to another company. -With Affiliates: We may share Your information with Our affiliates, in which case we will require those affiliates to honor this Privacy Policy. Affiliates include Our parent company and any other subsidiaries, joint venture partners or other companies that We control or that are under common control with Us. -With Business partners: We may share Your information with Our business partners to offer You certain products, services or promotions. -With other users: when You share personal information or otherwise interact in the public areas with other users, such information may be viewed by all users and may be publicly distributed outside. |
| GenePlanet | Employees of GenePlanet  Employees having access to your Personal Data are limited to those who have a “need to know basis” to ensure that you receive the purchased Products and are bound by the confidentiality agreement.  Contractual processors of Personal Data  We involve certain third parties which enable your Product purchase and delivery. Your Personal Data is only processed for purposes defined in this Privacy Policy, subject to GenePlanet’s documented instructions. Contractual processors are involved for:  -Biological sample analysis We engage trusted and certified laboratories where your biological samples are tested. The laboratory depends on the Product you have purchased. -Order fulfilment and shipping We engage third-party providers who ensure delivery of the Testing Kit and Test Result delivery following analysis. -Cloud storage, IT, and security GenePlanet outsources Personal Data storage and engages third-party providers for security purposes. In addition, GenePlanet engages third-party providers for IT services and maintenance that enable the tools we use for our operations related to our Products. -Marketing, advertising, and analytics -E-mail, SMS, and written notice sending providers -Transfer of Personal Data based on legal requirements  **From Website:** GenePlanet uses the latest software and information technology systems to ensure that your data will never be released to a non-authorised person without your expressed consent.  Authorised persons and/or service providers are contractually bound to process your data in accordance with our instructions and under safety standards of the national and European legislation.  Personal information is not disclosed to anyone outside of genetic performance nor within genetic performance except as needed by those assembling the report. The laboratory where the DNA is analysed knows only the sample code, so it is not possible to make a connection to the specific client. |
| Mass Science | N/A |
| My Toolbox Genomics | Except as otherwise stated herein, Toolbox will not share Personal Information about you with any third party, except under these circumstances:  -Carefully-screened partners or other service providers, such as contracted laboratories and laboratory processors, credit card processors, or data storage service providers, who receive information about you in order for you to use Toolbox Services; -Registration Information, Self-Reported Information, and Preference Information (but NOT Genetic Information) may be shared with strategic partners; I-f you have given consent for Toolbox Research, etc. -To help us connect with new audiences who have similar interests and demographics as our current customers, etc -In some cases, these third parties process Personal Information on our behalf etc.; -Toolbox may sell, transfer, or otherwise share some or all of its business or assets, including your Personal Information, in connection with a merger, financing, acquisition, dissolution transaction, bankruptcy proceeding, or reorganization or sale of Toolbox’s business or assets; -If Toolbox is required by law and by the appropriate authorities to do so (see the Information Disclosure Required By Law section below); or -If you have provided Toolbox with explicit consent to do so. |
| MyGeneRank | Except as described in this privacy policy, we will not sell, rent, lease, give away, disclose, or share your Personal Information to third parties without your consent. We may disclose your Personal Information in the following ways:  We reserve the right to disclose information collected through the MyGeneRank website and online services as required by law, when we believe disclosure is necessary to comply with a regulatory requirement, judicial proceeding, court order, or legal process served on us, or to protect the safety, rights, or property of those involved in MyGeneRank studies.  We may share your coded study data (data without your name, date of birth, or email address) with other researchers as permitted by the informed consent for MyGeneRank studies. Individual level genetic data collected by the MyGeneRank website and online services will not be shared. Aggregate genetic data and statistical results may be shared. |
| OH Data Port | Once you’ve created an account, you may import data from a variety of sources, such as research studies you’ve participated in. You can choose to share this data with third-party researchers, other Open Humans members, or the general public. Only the recipients you’ve selected will be granted access to your personal account data. Please understand that while we expect researchers to follow our Project Guidelines, Open Humans can’t control and isn’t responsible for the data handling practices of others.  Open Humans will share your non-public personal data with others only under these circumstances:  -with your consent, and after letting you know what information will be shared and with whom, unless otherwise permitted in this policy. -if we believe it is reasonably necessary to comply with a law, regulation, or valid legal process -to third parties helping us offer and improve our service, such as those providing Open Humans technical tools and analytics services that help us understand how people use our service. We require those companies to observe the limitations in this data use policy. -in rare cases, if you’re a participant in a study operated by Open Humans, with the Institutional Review Board (IRB) that oversees that study.  We may also share aggregate information with others – for instance, with our funders, to help them understand our community and how it uses our service.  If Open Humans or the Open Humans Foundation is involved in a reorganization, merger, acquisition or sale of our assets, your personal information may be transferred to another entity as part of that deal. If that happens, we will notify you and let you know what your options are. |
| Pattern Health | We may disclose Anonymized User-Provided and Automatically Collected Information:  -As required by law, such as to comply with a subpoena, or similar legal process; -When we believe in good faith that disclosure is necessary to protect our rights, protect your safety or the safety of others, investigate fraud, or respond to a government request; -With our trusted services providers who work on our behalf, do not have an independent use of the information we disclose to them, and have agreed to adhere to the rules set forth in this privacy statement. -If Pattern Health is involved in a merger, acquisition, or sale of all or a portion of its assets, you will be notified via email and/or a prominent notice on our website of any change in ownership or uses of this information, as well as any choices you may have regarding this information; -To analytics and customer support companies as described by this privacy policy. |
| Project Serotonin | We disclose your personal data as described below and as described elsewhere in this Privacy Policy.  -Third Party Service Providers. We may share your Personal Data with third party service providers to: provide you with the Services that we offer you through our Services; to conduct quality assurance testing; to facilitate creation of accounts; to provide technical support; and/or to provide other services to the Company. While we implement procedures and contractual obligations on our service providers designed to protect the confidentiality and security of your information, we cannot guarantee the confidentiality and security of your information due to the inherent risks associated with storing and transmitting data electronically.  -Affiliates. We may share some or all of your Personal Data with our parent company, subsidiaries, joint ventures, or other companies under a common control (“Affiliates”), in which case we will require our Affiliates to honor this Privacy Policy.  -Corporate Restructuring. We may share some or all of your Personal Data in connection with or during negotiation of any merger, financing, acquisition or dissolution transaction or proceeding involving sale, transfer, divestiture, or disclosure of all or a portion of our business or assets. In the event of an insolvency, bankruptcy, or receivership, Personal Data may also be transferred as a business asset. [...]  -Other Disclosures. Regardless of any choices you make regarding your Personal Data (as described below), Company may disclose Personal Data if it believes in good faith that such disclosure is necessary (a) in connection with any legal investigation; (b) to comply with relevant laws or to respond to subpoenas or warrants served on Company; (c) to protect or defend the rights or property of Company or users of the Site, Application, or Services; and/or (d) to investigate or assist in preventing any violation or potential violation of the law, this Privacy Policy, or our Terms of Use. |
| StuffThatWorks | SHARING INFORMATION WITH THIRD PARTIES The STW Community: Through your use of the Service, when you register as a member you may choose to share various data and information, including your name, STW profile photo or avatar, or possibly other Personal Information, with other registered members with the same condition(s), or with the broader STW community at large, as provided in the TOU for instance, when you choose to share your Reports with the relevant STW community, such Reports will be reflected in the chronic condition map and insights derived from such map shall be visible to other Users from your community who also elected to share their Reports. Sharing your information and experiences with other registred members is a way to add value to the community experience and makes the Service beneficial for everyone involved, and it will always be done with your explicit consent. Only those registered members in your community with reciprocal share settings will have access to your Report, all at your discretion. Third Party Services: We are partnering with a number of selected service providers, whose services and solutions complement, facilitate and enhance our own. [...] Such Third-Party Service Providers may receive or otherwise have access to your Personal Information, depending on each of their particular roles and purposes in facilitating and enhancing the Service, and may only use your Personal Information for such purposes. Such disclosure or access is strictly subject to the recipient's or user's undertaking of confidentiality obligations, and the prevention of any independent right to use this data except as required to help us provide the Service -Our Partners. We will not share your personal information without your explicit consent, with our valued partners, including but not limited to those in the medical, pharmaceutical and biotechnology industries, academic institutions, and government agencies and regulatory bodies, including regulatory bodies such as the American CDC and FDA, or other national and international bodies, as applicable and as necessary. We may share only de-identified and/or aggregated information with Partners, in order to conduct scientific, and/or medical research, as part of our Service. When disclosing information to our ---Partners or otherwise selling user information for scientific or market research purposes, we make sure to anonymize and/or remove all Personal Information or other personally-identifying indicators in the data (de-identification) to minimize the possibility of accidental member identification. -Law enforcement, legal proceedings, and as authorized by law: [...] Protecting Rights and Safety: We may share your Personal Information with others, with or without notice to you, in cases of emergency or if we believe in good faith that this will help protect the rights, property or personal safety of our Company, any of our Users, or any members of the general public. -Our Staff and Affiliated Companies: We may share Personal Information internally at STW and within our family of companies, for the purposes described in this Privacy Policy. Should we undergo any change in control, including by means of merger, acquisition or purchase of substantially all of its assets, your Personal Information may be shared with the parties involved in such an event. [...] For the removal of doubt, we may share your Personal Information in additional manners, pursuant to your explicit approval, or if we are legally obligated to do so. Additionally, we may transfer, share or otherwise use non-personal information in our sole discretion and without the need for further approval. |
| Urban Mind | Your participation in the Urban Mind research project is anonymous, private and confidential. The information you provide will remain with King’s College London, and may be shared securely with the project partners (J&L Gibbons and Nomad Projects) and with other organisations collaborating on the project for research purposes. The information you provide may also be shared securely with organisations who provide services to us in connection with the research purposes, for example data storage, as stated in this Privacy Policy. We will not disclose your information to unrelated organisations or third parties under any circumstances. |
| Withings Health mate | DATA SHARING. We only share such data in circumstances described below:  a. Your control over the Data. You may ask us to disclose information to others, such as when you use our community features like forums or programs that require sharing with third parties. You can change your choices at any time by changing your account settings or by visiting our Help Center.  b. Internal and Legitimate Sharing. Personal Data may be processed by the employees of WITHINGS SAS and its subsidiaries, within the limits of their respective responsibilities and exclusively for the purposes described in this Policy.  c. Use of our subcontractors. We share certain Data with subcontractors, who are experts in their field, in order to supply the Products and Services. Our subcontractors are required to comply with both the GDPR. They process the shared Data only for the intended purpose. Our subcontractors help us to provide you with high quality products and services, please find the list of subcontractors here.  d. Use of ScanWatch in the United States. WITHINGS may share certain personal information (name, date of birth, email, address, phone number) with Heartbeat Health, a U.S. company, which provides you with services such as the prescription necessary for the ECG functionality of the device, the organization of teleconsultations with our health professional partners, the provision of advice on your health. Your consent to receive text messages from Heartbeat Health is required to activate the ECG functionality on your device. Please see Heartbeat Health's privacy policy for more information.  e. Legal reasons. We may share Personal Data relating to you when required by law, upon request of a court, in connection with a legal proceeding [...] |
| ActiveDay - Activity Study | We do not share your information except as listed here and in our Privacy Notice (available at https://becklar.com/workforce-safety/privacy-policy/). Our Products may share your information to emergency contacts you list or to other users to whom you are connected, such as, for example, members of an organization to which you have connected your account. Your location information may be shared with others and personal identifiers, device data, and contact information will be provided to emergency contacts. We will also provide your information to third parties who help Us provide Our Sites and Products to you.  If Becklar, or substantially all its assets are acquired, the maintenance of all collected personal information may be transferred to the acquiring party, provided that the acquiring party implement a privacy and security policy at least as restrictive and secure as Becklar’s present policy in relation to personal information. |
| ADHD - Cognitive Research | We never sell the personal information of our users. We do not share your personal information except in the limited circumstances described below.  Profile: By default, only your name (and photo if you have provided one) is visible to other users of the Services. You may direct us to disclose more information to others, such as when your account is linked to a professional or to your parents (see next paragraph) or when you use our community features like the challenges and other social tools. For certain information, we provide you with privacy preferences in account settings and other tools to control how your information is visible to other users of the Services.  Professional and family accounts: A parent, educator, health professional, or researcher may grant you access to the Services and access your personal data, such as your cognitive activity and results. We ask for your explicit consent before sharing your data with such person and we provide you with privacy preferences in account settings and other tools to enable or disable this sharing feature.  Third-Party Services: Subject to your account settings, other services may look-up your profile. When you opt to link your account with other services, personal data will become available to them. The sharing and use of that personal data will be described in, or linked to, a consent screen when you opt to link the accounts. [...]  Service Providers: We transfer information to our corporate affiliates, service providers, and other partners who process it for us, based on our instructions, and in compliance with this policy and any other appropriate confidentiality and security measures. These partners provide us with services globally, including for development, maintenance, customer support, information technology, payments, sales, marketing, data analysis, research, and surveys. They will have access to your information as reasonably necessary to perform these tasks on our behalf and are obligated not to disclose or use it for other purposes.  Law, Harm, and the Public Interest [...]  Affiliates and Change of Ownership: If we are involved in a merger, acquisition, or sale of assets, we will continue to take measures to protect the confidentiality of personal information and give affected users notice before transferring any personal information to a new entity.  Non-Personal Information: We may share non-personal information that is aggregated or de-identified so that it cannot reasonably be used to identify an individual. We may disclose such information publicly and to third parties, for example, in public reports about exercise and activity, to partners under agreement with us, or as part of the community benchmarking information we provide to users of our subscription services. |
| Andaman7 Private Health Record | Are your non-medical personal data processed by subcontractors? Yes, A7S can ask a subcontractor to process personal data exclusively on behalf of A7S and on the latter's instructions. A7S contractually ensures that the subcontractor cannot process this data for own purposes independent of the purposes for which A7S uses the subcontractor. |
| Atlas Health | Disclosure for Law Enforcement  Service Providers We may employ third party companies and individuals to facilitate our Service (‘Service Providers’), to provide the Service on our behalf, to perform Service-related services or to assist us in analyzing how our Service is used.  These third parties have access to your Personal Data only to perform these tasks on our behalf and are obligated not to disclose or use it for any other purpose.  Analytics We may use third-party Service Providers to monitor and analyze the use of our Service. [Followed by list of service providers]   Behavioral Remarketing Atlas Health Europe Limited uses remarketing services to advertise on third party websites to you after you visited our Service. We and our third-party vendors use cookies to inform, optimize and serve ads based on your past visits to our Service. [list of marketers] |
| Behavidence Research App | WHO PROCESSES YOUR DATA We will not share your information with third parties, except in the events listed below or when you provide us your explicit and informed consent.   We will process information with our service providers helping us to operate the Website.   We will process your Website Analytics Information with the assistance of our service providers who assist us with the internal operations of the Website. These companies are authorized to use your statistical information, which does not contain any identifying details about you, in this context only as necessary to provide these services to us and not for their own promotional purposes.  We will share information with competent authorities, if you abuse your right to use the Website, or violate any applicable law.   If you have abused your rights to use the Website, or violated any applicable law, we will share information with competent authorities and with third parties (such as legal counsels and advisors), for the purpose of handling of the violation or breach.   We will share your information if we are legally required.   We will share your Information with third-parties in any event of change in our structure.  [...]we will share information only as required to enable the structural change in the operation of the business. |
| Better - Rewards for Health | We do not sell any of your Personal Information or Personal Health Data to any third parties. Your information is encrypted and not shared unless you specify otherwise.  From Website:  Data Ownership Store and manage your personal health data securely on the blockchain, giving you ownership and control over your information |
| Chemo Brain Cognitive Research | N/A |
| Depression Cognitive Research | N/A |
| DNA Fit | Except as otherwise stated in this Privacy Policy, Terms of Service or Consent Document we will never share your Information with a third party without getting your consent to do so, unless we are required by law. If we are legally required to disclose any Information, we will make reasonable efforts to notify you unless we are legally prohibited from doing so.   We will only share your Personal Information with those categories of third parties listed below and under these circumstances or as detailed in the Consent Document:  -Nutrition information will be shared with our third-party application known as “Meal Planner”; -Current or future Prenetics global entities. As Prenetics grows, restructuring may take place and it may be appropriate for more than one entity to control and process Information. This Privacy Policy will apply to all Prenetics entities unless otherwise stated; -Contracted consultants, suppliers and partners used to undertake fundamental activities to enable us to provide our services, enhance the User experience; and to effectively operate and manage our organisation;  -Card processing service providers;  -Companies that do services to get your purchases to you, such as payment service providers, warehouses, order packers and delivery companies. -Research contractors where you have given consent to participate in Prenetics Research and R&D. Research contractors will only be granted access to your Genetic Information and Self-Reported Information through online channels and at Prenetics’s offices for approved scientific research purposes. Research contractors will be screened and will be subject to the rules established by Prenetics, any Information sharing agreements that we may implement, this Privacy Policy and the Consent Document; -Where we are required by Applicable Law and by the appropriate authorities to do so; or -With anyone else as provided for in terms of your explicit prior consent to do so. Any Processors or other third-party service providers will be required to contractually comply with the principles and objectives of any Prenetics policies, including this Privacy Policy, as well as the requirements of the EU GDPR, the UK GDPR and DPA 2018 and other Applicable Law and will be required to sign a data processing agreement to confirm that Information will not be collected, used, shared, stored or otherwise for any Purpose other than those instructed by Prenetics EMEA. |
| Dyscalculia Cognitive Research | N/A |
| Dyslexia Cognitive Research | N/A |
| Fibromyalgia – Research | N/A |
| Google Fit | Limited Uses of User Data for Health Research Health Research Applications or Web Services must comply with the below requirements. These requirements apply to the participant data, including the raw data obtained from Google Fit APIs, and data aggregated, anonymized, or derived from the participant data or the raw data.  Limit your use of participant data to its intended purpose for collection. -Immediately de-identify participant data to the greatest extent possible for your study. -Do not use or share participant data with third parties for new research studies, or for any purpose different from the original study purposes, without obtaining a separate informed consent from participants, unless the IRB explicitly waives the requirement for separate informed consent. -Do not use or share data with members of your team who do not have a genuine need to know. -Only transfer participant data to third parties: -If necessary to pursue the original research purpose and the third parties are bound to -limit its access and use of the participant data to fulfilling that purpose; -If the participant granted explicit consent to share specific data; for example, in the signed Informed Consent document presented to the participant; -if necessary for security purposes (for example, investigating abuse); or, to comply with applicable laws or regulations. All other transfers, uses, or sale of participant data is expressly prohibited, including:  1. Transferring, selling, or using participant data for serving ads, including contextual, retargeting, personalized, or interest-based advertising. 2. Transferring or selling participant data to third parties like advertising platforms, data brokers, or any other information resellers. 3. Transferring, selling, or using participant data to determine credit-worthiness or for lending purposes. 4. Transferring, selling, or using the participant data with any product or service that may qualify as a medical device pursuant to Section 201(h) of the Federal Food Drug & Cosmetic (FD&C) Act if the participant data will be used by the medical device to perform its certified function. 5. Transferring, selling, or using participant data for any purpose or in any manner involving Protected Health Information (as defined by HIPAA) unless received by you under a valid HIPAA Authorization that was reviewed by an IRB or EC, as applicable. |
| Happiness Project - Play Games for Science | What data will I be sharing? Your test scores and the basic details you give us in the app will be sent to us. Some anonymous passive data from your phone’s sensors and your responses to occasional questionnaires will also be sent to us.  Is it anonymous? Yes. The data collection is anonymous and furthermore we won't use your information at any point to try to identify you. We don't ask for your name or for any other personal details, and we don't need your phone number to send notifications to your phone. We will never sell your data to any third party. We may make anonymous data available for further research by other parties such as academic researchers at other institutions. |
| Healthy Minds Program | Who We Share Your Information With We limit the release of information to that described in this Privacy Policy, and we require privacy protections in our business relationships. We do not sell customer information for monetary consideration. In addition, we may share your personal information when required by law, or with third parties that perform functions on our behalf. We may share personal information with third party organizations or individuals if we have a good-faith believe that it is reasonably necessary to meet any applicable law, regulation, subpoena, legal process or enforceable government request; to detect, prevent, or otherwise address fraud, misuse, or any security or technical issues; and/or to protect against harm to the rights, property, or safety of HMI, our users, or the public.  If the App is provided and used in connection with any educational program in which you are participating, we may provide your educational institution with personally identifiable information regarding your usage of the App.  If the App is used in connection with a research study in which you are participating, our privacy practices with respect to that research study will be provided to you separately and at that time.  We may also share de-identified and aggregated data with third parties for research, analytics, or other purposes allowed by applicable law. |
| Hevy Gym Log Workout | These services have the purpose of hosting and running key components of this Application, therefore allowing the provision of this Application from within a unified platform. Such platforms provide a wide range of tools to the Owner – e.g. analytics, user registration, commenting, database management, e-commerce, payment processing – that imply the collection and handling of Personal Data. Some of these services work through geographically distributed servers, making it difficult to determine the actual location where the Personal Data are stored. [...]  This type of service allows this Application to access Data from your account on a third-party service and perform actions with it. These services are not activated automatically, but require explicit authorization by the User.  By registering or authenticating, Users allow this Application to identify them and give them access to dedicated services. Depending on what is described below, third parties may provide registration and authentication services. In this case, this Application will be able to access some Data, stored by these third-party services, for registration or identification purposes.  The User's Personal Data may be used for legal purposes by the Owner in Court or in the stages leading to possible legal action arising from improper use of this Application or the related Services. |
| Huawei Health | Huawei Health supports sharing of fitness and health data from your device to other Huawei (e.g. Vascular Health service and Huawei ECG service) and third-party (e.g. Google Fit) health and fitness apps. Such data sharing is conducted only based on your prior authorization.  Huawei Health enables you to make your Huawei sports watch available for other health and fitness apps. Such third-party apps can integrate and access your watch (e.g. to get heart rate or other sensor data, to send notifications to your watch, etc. depending on the scope of the authorization) only after your explicit authorization.   We may also share your data in the following circumstances:  •Our third-party vendors, who provide us with IT (including cloud-based) services and business support, may need to process your HUAWEI ID information. All such third parties are operating under contract and acting on behalf of us and are located in the EU/EEA.  •For financial management purposes, we will transfer your order information to a Huawei affiliate in China operating under contract and acting on behalf of us.  •If you are using Huawei Health in Türkiye, our business operations, operating under contract and acting on behalf of us, may require us to access your data from Türkiye. Our business operations in New Zealand, operating under contract and acting on behalf of us, may be required to access your data, if you are using the service in New Zealand or Australia.  •We may share your data in response to a legal process or request from a competent authority according to applicable laws or in connection with a legal proceeding or process.  •Your data may also be disclosed as part of a merger, acquisition, sale of assets (such as service agreements), or transfer of services to a Huawei group entity or another company. |
| InsideTracker | We may disclose aggregated, or other non-Personal Information or information about our users without restriction. We may disclose Personal Information about you in the ways described below and/or to the third-parties mentioned below.  -Affiliates. To our parents, affiliates, joint ventures, or promotion partners, for their use in a manner consistent with the purposes described in this Privacy Policy. -Agents and Service Providers. To contractors, service providers, and other third parties we use to support our business and provide the Services. These providers may complete transactions or perform services on our behalf or for your benefit. -Marketing. With your consent, we may share your personal data to third parties for their own direct marketing purposes, to provide you with information about products that may be of interest to you, and for other purposes as specifically set forth in this Privacy Policy. We do not share any DNA data, DNA analysis or blood test data with third parties for marketing purposes. -Legal Process. As required by law, such as to comply with a subpoena or other legal process, or when we believe in good faith that disclosure is necessary to protect our rights, protect your safety or the safety of others, investigate fraud, or respond to a government request. If we are required to disclose your information, we will do our best to provide you with notice in advance, unless we are prohibited by law from doing so. -Certain Business Transfers. As part of a corporate business transaction, such as a merger, acquisition, joint venture, or financing or sale of company assets. It may also be disclosed in the event of insolvency, bankruptcy, or receivership. We will use reasonable efforts to direct any such transferee to use your Personal Information in a manner that is consistent with our Privacy Policy. -Consent. In additional ways upon your consent. |
| Insomnia - Cognitive Research | N/A |
| Medisafe Pill & Med Reminder | WITH WHOM DO WE SHARE YOUR PERSONAL INFORMATION -Research partners – we may share your Personal Information with third parties, such as research institutes, healthcare systems and healthcare providers. They may associate it with other information that they have about you, for improved healthcare, research purposes and the improvement of our Service. Providers of personalized third party content – from time to time, we may also ask whether you would like us to share your Personal Information with another company that may want to send you information about their products or services. If you consent to such transfer by us of your Personal Information to another company, please note that the information provided will be subject to such company’s privacy practices and shall not be within our control. -Content providers – we may also use your Personal Information in order to provide you with personalized third party content or links to third party sites that might interest you. We provide this third party content and/or links to third party sites for information purposes only and are not liable for such content or sites. [...] -Third Party Health Apps – we participate in Apple HealthKit, Samsung S-Health and Google Fit frameworks. We will use information that you consent that we receive from those third party health apps for medical research purposes, internally in order to improve our products and services and in the form of aggregated and/or analytical data as described in the Use of Aggregated Data section of the Privacy Policy, and may transfer this data to third parties for such purposes. We may provide users with the ability to share their Medisafe data with other authorized services on their device after receiving their consent for the same. We will not sell any Personal Information nor will we use this data for advertising purposes. Please note that when you share information with third party apps, such information will be subject to their privacy policy, over which we have no control. In order to learn more, please visit each of Apple, Samsung, or Google’s relevant pages and their respective privacy policies. -Service providers – we may share your Personal Information, as is reasonably necessary, with our service providers, [...] provided that we will only share Personal Information to the extent necessary with such service providers. - Law authorities [...] -Merger, acquisition or sale |
| MyTherapy Pill Reminder | Temporarily sharing progress with health care professional  You can choose to share your progress with a health care professional using a one time code that can be entered in a web dashboard. The health care professional can view your progress for 15 min. The code expires automatically after use. Please only share the one time code with trusted persons.  Cooperation with pharmacy partner  We cooperate with pharmacy partners to give you access to pharmacy services such as filling your digital prescriptions (“eRx”), participating in pharmacy loyalty programs or receiving pharmaceutical counseling as set out in more detail below (“Pharmacy Functionality”). We will ask you for your specific consent before you can use the Pharmacy Functionality. The consent text will provide you with more information on the specific pharmacy partner in your country. When you consent to the Pharmacy Functionality, we will share your user ID and personal health data with the pharmacy partner and the pharmacy partner will share such data with us, to the extent this is required to provide the Pharmacy Functionality. To use the Pharmacy Functionality to the full extent, it might be necessary to pair your User Account with the account you have created with the pharmacy partner.  The legal basis for processing your personal data for this purpose is your consent (Article 6 para. 1a) and Article 9 para. 2a) GDPR). Exporting MyTherapy medication reminders and inventory  We share your medication and inventory information with our pharmacy partner. This will allow you to trigger refill reminders in MyTherapy and purchase medication with low inventory from our pharmacy partner. Also, this may allow you to monitor your medication inventory on our pharmacy partner’s website and app.  g) Joint data controllership  We together with the pharmacy partner may qualify as “joint data controller” in terms of Art. 26 GDPR with regard to single Pharmacy Functionality. We have entered into a respective arrangement with the pharmacy partner. We will make the essence of the arrangement available to you upon your request.  If you withdraw your consent with regard to the Pharmacy Functionalities, we do not longer share personal data with the pharmacy partner and delete any related data that we do not need for providing other functionality in MyTherapy. Please note that the pharmacy partner may still retain and process the shared data for other purposes in the pharmacy partner’s sole responsibility. Please refer to the pharmacy partner’s privacy policy.  Requesting a prescription from your physician  When running low on your prescription medication, you can request a refill prescription from your consulting physician. We share your name, required prescription including medication name and package size, as well as the name of your physician with our pharmacy partner, who will forward this request to your physician |
| NeuroPsy Research | Sharing of Research Data When a user decides to share the collected research data with us, the data will be transferred to a database on servers within the European Union which are provided by a third party. You can find out more about the data protection measures of the third-party provider at the following Internet address: https://www.heroku.com/policy/security#data-security  After transfer of the research data to the database, complete deletion of the research data collected can no longer be guaranteed, since we can not attribute the anonymous data to a specific person. Furthermore, backup copies of the data are created within the infrastructure of the third-party provider. Moreover, immediate public access to the research data and its results from automated processing is possible, see Usage of Anonymized Data. |
| Parkinson’s Cognitive Research | N/A |
| Renpho Health | Protection of Renpho and Others – If we believe the release of data about you is necessary to protect the rights, property, and safety of others, to respond to legal process, or to investigate or remedy potential violations of our policies, we may share your data as permitted or required by any applicable law, rule, or regulation. This includes contacting emergency responders or law enforcement, as well as exchanging data with other entities for fraud protection and credit risk reduction.  Acquisition – In the event that our company or substantially all of its assets are acquired, your personal data may be one of the transferred assets.  With Your Consent – We may disclose your personal data with your express consent.  We do not sell any personal data to third parties. Sometimes we send offers to selected groups of Renpho customers on behalf of other businesses. When we do this, we do not give that business your personal data.  Please note that if you voluntarily submit any personal data for posting on the Services, including social media, such as a review, comment, or “like,” the information becomes publicly available and can be collected and used by others, so you should use care before posting information about yourself online.  In some jurisdictions, individuals may have the right to opt-in or withdraw consent for certain uses. If you reside in such jurisdictions, you may have additional rights which are detailed below, in “User Rights and Choices Regarding Personal Data,” “California Privacy Rights,” or “European Data Protection Rights.” |
| Smart Omix by Sharecare | -With Your Consent. We may share your Personal Information with companies, organizations or individuals outside of Sharecare when we have your consent to do so. -As Part of an Enterprise Program. We may share your information according to the direction of your Enterprise Organization and any privacy policy and/or contractual requirements that they may ask us to comply with. Your Enterprise Organization may require Sharecare to share your Personal Information with the following: - Enterprise Organization. Under U.S. laws, we may share PHI with your health plan for the administration of your plan. If you receive Services through your relationship with a non-US-based Enterprise Organization, we will adhere to the applicable laws in your country. - Healthcare Providers. We may share information with your healthcare providers and any clinics or organized healthcare organizations with whom they are associated, provided such sharing is allowable under HIPAA. - Your Employer. We will not share your PHI with your employer for employment-related purposes. We may only share the information needed for your employer to deliver Enterprise Programs. For example, we may share completion status of a wellness plan requirement but not the actual results of the required action or activity. - Third-Party Service Providers. We may disclose your PHI to our business associates who perform various functions on our behalf, but Sharecare requires these third parties to agree in writing to safeguard your PHI appropriately and in accordance with the law. Sharecare does not sell or rent your PHI to third parties. Sharecare does not use your PHI to market, sell, or otherwise promote goods or services that are not health-related benefits provided by your health plan, employer, or provider. -For External Services. We may, from time to time, outsource some or all of the operations of our business to third-party service providers. In such cases, it may be necessary for us to disclose your information to those service providers. In some cases, the service providers may collect information directly from you on our behalf. We restrict how such service providers may access, use and disclose your information. We employ other companies and individuals to perform functions on our behalf. Examples include processing compensation, providing employee benefits, and performing legal and other professional services. These agents may have access to your information as needed to perform their functions, but they are not permitted to use it for other purposes. -As Part of a Community Program. If you participate in a Community Program, we may also disclose your Personally Identifiable Information to Blue Zones, LLC, an independent entity that has entered into a partnership with Sharecare that allows the partners to work together to support Blue Zones Projects. Blue Zones, LLC is not bound by this Privacy Policy. Your information also will be shared with your Blue Zones Project Community Sponsor, and may also be shared with your employer if your employer is sponsoring a Worksite project under a contract with Sharecare. - For Legal Reasons. [...] -In a Transaction. As we continue to develop our business, we might sell, buy or merge with companies, subsidiaries, or business units. In such transactions, data generally is one of the transferred business assets but remains subject to the promises made in any pre-existing privacy statement (unless, of course, the person consents otherwise). Also, in the unlikely event that Sharecare or all of its assets is acquired, your information may be one of the transferred assets. - After Being Fully Deidentified, Incapable of Reidentification. We may share aggregated, de-identified information publicly and with our partners to evaluate the effectiveness, value, and analytic trends of the Services.Table3[@[Privacy/Confidentiality]] |
| Symptom & Mood Tracker | We will share some of your personal data with third parties outside Bearable only where it is strictly necessary to provide our services to you, where required by law, where we have another legitimate interest to do so or where you specifically consent to it. Personal data may be shared in the following circumstances:  -Third Party Support Service Providers: For example: technology service providers, payment providers, data storage providers, and marketing platforms (e.g. for sending marketing emails), -Third Party Apps: some of your personal data may be shared with other Apps but only if you agree to that. Please see the section below for further information. -When required to do so by law, regulation or court order; in response to a legitimate request for assistance by the police or other law enforcement agency.  -Please note that as a UK company, we’re not required to have to comply with US criminal subpoenas requesting disclosure of data, in light of Roe v Wade. to seek legal advice from Bearable’s external lawyers or in connection with litigation with a third party; or in connection with the sale, purchase or merger of our business. |
| Symptomate - Symptom Checker | The recipients of your personal data may include:  entities authorized by law on the basis of a proper request (courts, state authorities); entities providing accounting, IT, marketing, communication, analytical and legal services, including HubSpot, Google, Facebook, FullStory, Amplitude; subcontractors with whom we cooperate. Your information, including personal data, may also be transferred to — and maintained on — computers located outside of the European Economic Area, where the data protection laws may differ from the GDPR.  If we provide the personal data beyond the European Economic Area, and in particular to any third countries, such provision will take place on the basis of appropriate legal mechanisms, such as Executive Decisions of the Commission (EU), standard contractual clauses applicable, or other similar legal instruments specified in the content of GDPR. In addition, we follow the Post Schrems II recommendations adopted by the European Data Protection Board.  To ensure that you have adequate control over your personal data transferred outside the European Economic Area, you will have the right to obtain a copy of your personal data transferred to third countries at any time. |
