# Supplementary material for: Mapping the Apps: Ethical and Legal Issues with Crowdsourced Smartphone Data using mHealth Applications
Source: Asian Bioeth Rev. 2024 Jun 18;16(3):437–70. doi: 10.1007/s41649-024-00296-3 (PMC11250705; doi:10.1007/s41649-024-00296-3)
Supplement: Supplementary file 5 — (DOCX 29.2 kb) [file 41649_2024_296_MOESM5_ESM.docx]

| Appendix 4: Mentions of Privacy and Confidentiality | |
| --- | --- |
| App | Mentions of Privacy/Confidentiality |
| 23andMe - DNA Testing | Can opt out of the following services: storing your sample, viewing your health reports, sharing features, personalized recommendations, communications preferences, research participation.  Third Party Content and Integrations Our Services may contain third party content, integrations or links to third party websites operated by organizations not affiliated with 23andMe. Through these integrations, you may be providing information to the third party as well as to 23andMe. Since we can only control our own Services, we are not responsible for how those third parties collect or use your information so please review the privacy policies of every third-party service that you visit or use.  Federal, State, and Region-Specific Information Federal and state laws (including the federal Genetic Information Non-discrimination Act or “GINA”) provide some protection from employer and health insurance discrimination based on your genetics.   Retention of Personal Information  Information is retained for as long as necessary to provide the services. 23andMe and/or our contracted genotyping laboratory will retain your Genetic Information, date of birth, and sex as required for compliance with applicable legal obligations  From Website:  We exceed industry data protection standards and have achieved 3 different ISO certifications to demonstrate the strength of our security program. There's also a privacy video. https://www.23andme.com/en-ca/privacy/ |
| Ada - Check your Health | we do not transfer your personal data to third parties - with the exception, when applicable, of the purposes listed below.  If we sell or buy any business or assets, we may disclose your personal data to the prospective seller or buyer of such business or assets.  If we are required on the basis of EU law or the law of a Member State to disclose or share your personal data.  We may disclose certain data to organizations involved in clinical trials and other types of research where you have explicitly authorized us to do so.  Ada does not link any usage profile with your personal data.Transfer of personal data to processor Hound Technology Inc.   We restrict access to your personal data to the persons who need to use it for the relevant purpose(s), always in compliance with the integrity and confidentiality principle.   From Website Describes security processes and defines highest regulatory standards |
| Ancestry: Family History & DNA | What Information Do We Share, When Do We Share It and Who Are the Recipients?  Ancestry does not share your individual Personal Information (including your Genetic Information) with third parties except as described in this Privacy Statement or with your additional consent. We do not voluntarily share your information with law enforcement. Also, we will not share your Genetic Information with insurance companies, employers, or third-party marketers without your express consent. |
| Apple Research | Despite our security measures, total confidentiality cannot be guaranteed. When you download one of the Apps and enroll in one of the Studies, below are examples of the categories of information (which may include some personal information) that may be collected from or about you through the Apps ("Study Data"). The specific categories of information that will be collected about you as part of a Study will be described in the Informed Consent.  -Contact information  -Demographic information, such as your age, gender, state of residence, and race. -Medical history and information. This information may be collected through in-app surveys or other health surveys that you may be asked to complete. -Sensor information, such as heart rate and beat to beat calculations. -Technical data, which in many cases is non-personal information. and technical data about your device, such as operating system and model. Apple does not track its customers over time and across third party websites to provide targeted advertising and therefore does not respond to “Do Not Track” (DNT) signals. -Adverse event information, such as a concern, adverse event, or other reportable matter arising in a Study. -Other information, if any, described in the Informed Consent. Your Contact information will be separated from the rest of the data collected from you through the Apps and as part of the Studies and replaced with a random code ("Coded Study Data") before it is received on Apple systems.   From Website: Nothing is more personal than your health information. Helping you keep it confidential and secure is as important to us as it is to you. What you choose to share for research, and with which study, is controlled entirely by you. Any data collected through the Research app will be encrypted if you have a passcode set on your device. Once shared, it is stored securely in a system within Apple that is designed to meet the technical safeguard requirements of the Health Insurance Portability and Accountability Act. Apple will not have access to any contact information or other identifying data that you provide through the Research app. And you can withdraw from any study at any time, ending any future data collection. |
| CovidWatcher | The data you track through CovidWatcher will be handled securely to maintain confidentiality. All data collected through the app is stored on secure servers. To further ensure confidentiality of data gathered throughout the duration of the study, identifying information will be kept separate from other research data collected by the app. Your data may be shared as part of research collaboration with other research teams. However, only the ‘de-identified’part of your data will be shared. We will not share any of your data with any non-research third party. To opt out of the study, please use the 'Withdraw from Study' button in the profile page of the CovidWatcher app. Once you withdraw from the study, we will not collect any further information from you. We may continue to use the data collected prior to withdrawing  From Website: How safe is my information?  Your privacy is our priority. You can choose to answer the surveys in an anonymous fashion on the Web, or download the CovidWatcher app where you will sign an informed consent to collect some additional information like your name and email. Whether you decide to use the website or the app, we keep all identifiable information separate from the collected data. We also carry out our analysis of the different needs in an aggregated way across all participants; thus we never disclose any data from any individual person.  Finally, all collected data are kept on highly secure, HIPAA-certified servers, and our team has specific training to keep your information safe. All data collection and analysis has been reviewed and approved by the Columbia University Institutional Review Board (Protocol #AAAS9690). How will my information be used?  The information gathered through your surveys will be analyzed by data-science researchers at Columbia University. They will be able to generate maps that reveal which neighborhoods and communities are in most need of various resources and reports that highlight different trends in time of needs. We are making the data available in an anonymous, de-identified fashion to our community partners. You can also access these maps and reports to track the impact of the pandemic on your neighborhood and your community |
| DNA ID, Inc. | Data privacy and protection is of the utmost importance. We utilize industry best practices to ensure that there is zero-knowledge when it comes to the transaction of sharing your genomic and health data.  From Website: HIPAA-Grade Security Our security is compliant with the Health Insurance Portability and Accountability Act (HIPAA) requirements regarding protected health information.  Double-opt-in sharing Our goal is to get you to share your data with researchers, but we abide by the double-opt-in rule. Meaning we won’t share your data with anyone you don’t want.  No secondary data usage We will never use or sell your DNA data, even anonymously, for secondary usage. It’s your DNA and we want you do determine what happens with it.  Completely deletable It’s your data, do what you want with it. You can delete your data on our server whenever you want. |
| DnaNudge | DnaNudge will destroy your DNA sample. We only collect and maintain personal data insofar as is necessary for the proper functioning of the Service; The results of Users’ genetic data processed on our servers are not linked to the individual User (except to the User’s anonymous account details, to allow us to provide the Service); We limit and control access to records of personal data to members of staff and Related Parties that require such access to perform their duties and services, through passwords, variable log-in rights and other technical and organisational access controls; The DnaNudge test results are available on your Mobile App and Capsule and are protected by a password which you are required to set up in order to access this information; We apply security measures (including as part of the cloud services we use and when using the services of Related Parties to process your data) including encryption, firewalls and physical security for our servers and information centres. We ensure confidentiality obligations are put in place when dealing with our Related Parties and other third parties; We avoid the collection or storage of personal data when it is unnecessary or for longer than reasonably needed or legally permitted or required and erase it (or anonymise it) once we no longer need it or are no longer required to keep it as personal data; User’s DnaNudge test results and the User’s account details are held in our records for as long as the User maintains his or her account. The data is erased when the User’s account is closed down. Data collected from monitoring Users’ use of the Service is aggregated and anonymised before we share it with third parties. |
| FLARe Research | N/A |
| Gene Doe | The security of Your Personal Data is important to Us, but remember that no method of transmission over the Internet, or method of electronic storage is 100% secure. While We strive to use commercially acceptable means to protect Your Personal Data, We cannot guarantee its absolute security. |
| GenePlanet | GenePlanet maintains a comprehensive data protection program using administrative, physical, and technical protection measures to safeguard our users' Personal Data.  We use measures to protect our users from inappropriate access, loss, misuse, or alteration of Personal Data (including genetic data).  The security team at GenePlanet regularly reviews the implementation of our security and privacy practices and upgrades them as necessary to ensure the integrity of our system and your Personal Data.  We use the latest security mechanism standards to process and store Personal Data (including genetic data). We only work with companies that meet and commit to our safety standards. While we cannot guarantee that there will be no loss, misuse, or alteration of Personal data, we strive to prevent this from happening.  From Website: Supreme data security All of your data is stored and handled according to European and local legislation and the highest security standards.  Privacy website:  When you (or your doctor, depending on the type of test) take a sample, it gets marked with an ID code and not your name. This is called pseudonymisation and means that people who come in contact with your sample (e.g. in the laboratory) do not know who you are.  This way, we ensure that the information provided by your sample cannot be used to identify you. |
| Mass Science | Mass Science securely stores your data on the Google Cloud platform and on encrypted servers at King’s College London. Only pseudo-anonymised and processed data will be available to analysts; actual location coordinates will be stored encrypted and only accessed to process into pseudo-anonymised metrics. We will keep your personal data for 2 years. Once this time period has expired, we will delete your personal data. |
| My Toolbox Genomics | Personal Information about users that is maintained on Toolbox’s systems and servers is protected using industry standard security measures. In order to secure your Personal Information, access to your data is password-protected, and sensitive data is protected by SSL encryption when it is exchanged between your web browser and the Toolbox website. However, no security measures are perfect or impenetrable, and Toolbox cannot guarantee that the information submitted to, maintained on, or transmitted from its systems will be completely secure. Toolbox is not responsible for the circumvention of any privacy settings or security measures contained on the Toolbox website by any users or third parties. |
| MyGeneRank | We will take reasonable technical and organizational precautions to prevent the loss, misuse, or alteration of your Personal Information. These precautions include:  All Personal Information that is collected by the MyGeneRank website and online services will be encrypted and electronically sent to a secure data server run by the Scripps Research Translational Institute (“STSI”), a non-profit research organization.  STSI will replace the direct identifiers associated with account (your email address) with a code to help protect your identity.  The coded, de-identified study data collected during participation in MyGeneRank research studies will be transferred electronically to a secure data server.   STSI will store the consent form and direct identifiers associated with informed consent (your name, signature, and email address) separately from all other information collected by the MyGeneRank website and online services.  Access to your MyGeneRank account will be protected by a password you select after enrolling in the study.   You acknowledge that the transmission of information over the internet is inherently insecure, and we cannot guarantee the security of data sent over the internet.  You acknowledge that we cannot guarantee complete security of the Personal Information we collect. Unauthorized entry or use, hardware or software failure, and other factors, may compromise the security of your Personal Information at any time. |
| OH Data Port | A note about inherent identifiability of your data Even if you use a pseudonym, you should be aware that data could be connected to your identity. Your basic profile information on Open Humans is public information. Public information is exactly what it sounds like: anyone, including search engines and people who are not users of Open Humans, will be able to see it. Once you’ve created an account, you may import data from a variety of sources, such as research studies you’ve participated in. You can choose to share this data with researchers, other Open Humans members, or the public. Only the recipients you’ve selected will be granted access to your personal data. Even if you use a pseudonym for Open Humans, your data could be connected to your identity. The risk of this varies depending on the type of data, but it is often surprisingly easy to identify people.  Some examples of identifiable data you may choose to share:  Demographic data. Just three pieces of information – your birth date, sex/gender, and ZIP code – are enough to uniquely identify most individuals. If you share these, someone may use them to figure out who you are. Genetic data. Your genome data can be used to learn about your ancestors, and this information can be used to identify you. For example, a man’s Y chromosome can be used to predict his last name (or “family name”) – this method has been used to identify individuals from “anonymous” genomes. Identifying people from genetic data is likely to get easier as genealogy tools become more powerful. Location data. Even a tiny amount of location data is enough to give a strong clue to someone's identity. Most people spend their time in two locations: work and home. That combination is often unique, and could be used to identify you. |
| Pattern Health | We value your privacy and will always do our best to protect your private data. Pattern Health products are designed for use by healthcare organizations subject to the privacy and security standards defined by the U.S. Health Insurance Portability and Accountability Act (HIPAA) and the E.U. General Data Protection Regulation(GDPR) 2016/679. We provide physical, electronic, and procedural safeguards to protect information we process and maintain. For example, we limit access to this information to authorized employees and contractors who need to know that information in order to operate, develop or improve our products. Please be aware that, although we endeavor to provide excellent security for information we process and maintain, no security system can prevent all potential security breaches. Should a breach occur, you will be notified within a reasonable time via email and/or a prominent notice on our website.  From Website Pattern Health’s platform is built for clinical research. We are HITRUST-certified and compliant with HIPAA, GDPR, and FISMA regulations to ensure the privacy and security of research studies. Additionally, Pattern Health is 21 CFR Part 11 compliant, meeting the FDA’s requirements for traceability, validation, assurance, and auditability. |
| Project Serotonin | N/A |
| StuffThatWorks | We take great care in implementing and maintaining the security of the Service and our Users’ Personal Information. We employ industry standard procedures and policies to ensure the safety of our Users’ Personal Information, reduce the risks stemming from loss of information and prevent unauthorized use of any such information. However, we do not and cannot guarantee that unauthorized access will never occur and reiterate that no measure can provide absolute information security.  From Website Q. What about privacy:  Transparency and giving users control over the data they share are at the core of what we do. We apply the highest level of security standards to all of our data, and are of course compliant with all applicable privacy laws. We encourage you to read our Privacy page and Terms of Use to learn more about the steps we take to protect our users and their data.  We conduct regular tests and reviews of our privacy and security measures, both internally and with a trusted third party. We also have an in-house Data Protection Officer (DPO) who is responsible for ensuring that we are completely compliant with the most up-to-date privacy standards and practices. He can be reached at dpo@stuffthatworks.health.  Please note, however, that while privacy is a main priority at StuffThatWorks, no security procedure can provide absolute information security. We do not and can not guarantee that unauthorized access will never occur.  What measures are taken to keep personal information secure?   We employ industry best practices to keep your information secure and private including the separation, encryption, de-identification and anonymization of your personal information. All processing and analysis is performed on a separate database made up of anonymized, normalized and aggregated data that does not include any personally identifying information. |
| Urban Mind | We take your privacy, confidentiality and the security of your information very seriously and we will take all reasonable precautions to protect your data from unauthorised access. This includes appropriately securing our electronic networks, servers, computers and physical facilities. We store information on King’s College London servers and in the cloud. Our external cloud service provider is currently Microsoft Azure. Microsoft Azure secures the data using technology called ‘256-bit advanced encryption’, which provides a very high level of security, and does not itself have access to the data. We reserve the right to use a different storage provider in future, and if we change provider we will update this Privacy Policy accordingly.  We will endeavour to ensure that our partners or any third parties to which we may transfer data according to the terms of this Privacy Policy have acceptable privacy and security policies. We will govern the sharing of information for research purposes or in relation to services provided to us in connection with the research purposes, by data sharing agreements which will ensure that third parties keep the data safe and secure. However, the collection and use of an individual’s information by such partners or third parties may be subject to separate privacy and security policies for which King’s College London is not responsible.  We collect the following data from you:  Information about you as an individual. When you use the Urban Mind app, you provide information about your age, gender, occupation, lifestyle and wellbeing. You also provide information about your current environment, feelings, thoughts and behaviour. You will not be asked to provide your name, contact details or any other information which could identify you. [...]  From Website: Is my participation private and confidential? Yes, your participation is completely private and confidential. The information you provide will remain with King’s College London, and may be shared securely with the project partners (J&L Gibbons and Nomad Projects) and other organisations collaborating on the project for research purposes. For further information, please see our Privacy Policy. Is my participation anonymous? Yes. We won’t know who you are. We do not ask for your name, address or any other identifying information, and we don’t require your phone number or email address. The data you provide cannot be linked to you as an individual. For further information, please see our Privacy Policy. |
| Withings Health mate | We pay maximum attention to the security of the hosting of your health data. We apply demanding regulations and standards. Thus, in addition to our compliance with the GDPR, we are certified ISO 27001 and HDS (Health Data Hosting) which allows us to provide you with the same level of security as health professionals. We host health data on our certified health data processing platform, whose servers are located in France, at a European operator (BSO).  This Policy applies uniformly to all Users of the Withings App, regardless of where you live. We take into consideration the regulations on the protection of personal data applicable to the markets in which WITHINGS sells its Products and Services. |
| ActiveDay - Activity Study | We have implemented measures designed to secure your personal information from accidental loss and from unauthorized access, use, alteration, and disclosure. All information you provide to us is stored on our secure servers behind firewalls.  The safety and security of your information also depends on you. Where we have given you (or where you have chosen) a password for access to certain parts of our Sites, you are responsible for keeping this password confidential.  Unfortunately, the transmission of information via the internet is not completely secure. Although we do our best to protect your personal information, we cannot guarantee the security of your personal information transmitted to our Sites. Any transmission of personal information is at your own risk. We are not responsible for circumvention of any privacy settings or security measures contained on the Sites.  Your California Privacy Rights  If you are a California resident, California law may provide you with additional rights regarding our use of your personal information. To learn more about your California privacy rights, visit https://fallsafetyapp.com/privacy-notice.  California’s “shine the Light” law (Civil Code Section § 1798.83) permits users of our App that are California residents to request certain information regarding our disclosure of personal information to third parties for their direct marketing purposes. |
| ADHD - Cognitive Research | We implement security safeguards designed to protect your data, such as HTTPS. We regularly monitor our systems for possible vulnerabilities and attacks. However, we cannot warrant the security of any information that you send us. There is no guarantee that data may not be accessed, disclosed, altered, or destroyed by breach of any of our physical, technical, or managerial safeguards. |
| Andaman7 Private Health Record | As part of the use of the Andaman7 application (hereafter referred to as A7), A7 Software (the company, hereafter referred to as A7S) processes various data and is therefore responsible for this processing. Thecontact details of the data controller can be found at the end of the document.  When you enter medical data into A7, the data resides only on your smartphone (and in any backups you create). Your medical data is not stored on A7S servers and A7S does not have access to your data. In accordance with the Regulation on data protection with regard to personal processing (specifically Article 9 on personal data relating to health), your medical data are only processed by A7S servers within the framework of 'an explicit relationship of trust created by you and with third parties within your' Circle of Trust 'such as parents, doctors, other healthcare professionals or organizations (hereafter “users”). The addition of third parties in the circle of trust is always initiated or explicitly accepted by the user. [...]  From Website Privacy is not negotiable We are patients, and as patients, we want to be in full control of our data and privacy. With Andaman7, your health data is only stored on your mobile devices. Andaman7, the company, does not have any access to it. You stay in control of who has access, and if you decide to share your data, you can withdraw your consent at anytime and stop sharing it. We protect your health data during transfer by encrypting it. Andaman7 is fully GDPR and HIPAA compliant. |
| Atlas Health | Your information, including Personal Data, may be transferred to — and maintained on — computers located outside of your state, province, country or other governmental jurisdiction where the data protection laws may differ than those from your jurisdiction.  The security of your data is important to us, but remember that no method of transmission over the Internet, or method of electronic storage is 100% secure. While we strive to use commercially acceptable means to protect your Personal Data, we cannot guarantee its absolute security.  From Website Privacy protection Your medical and personal data are safe at every stage, from ordering your kit to receiving results online. Read our Information Governance Policy to learn more about how we protect your privacy.  User Data are securely stored on certified servers located within the European Union SHA-256 with RSA Encryption Full UK GDPR and EU GDPR compliance Registered with Information Commissioner’s Office  How do you keep my private information safe? Atlas operates to the highest standards of information governance and customer privacy, as per our Information Governance Policy, including European Data Protection Regulation. The transmission of data is encrypted at the point of dispatch and receipt. Your data is stored in anonymised form on the Atlas servers in the United Kingdom, meaning that only Atlas software can read this information. All users have the right to access personal information that relates to them. All users have the right to request that all personal data is removed from the Atlas databases as per the ‘right-to-be-forgotten’ clause in the General Data Protection Regulation (both UK GDPR and EU GDPR) |
| Behavidence Research App | We implement measures to reduce the risks of damage, loss of information and unauthorized access or use of information. However, these measures do not provide absolute information security. Therefore, although efforts are made to secure personal information, it is not guaranteed, and you cannot expect that the Website will be immune from information security risks.  From Website 100% Private & Secure We don’t collect any identifiable or personal data - users are fully anonymized. We use top systems to protect data and encrypt by AES 256. No Content Tracked We don’t track any content in other apps - so no messages, typing or sites visited. This is purely a tracking behavior and engagement system. How does Behavidence keep my data secure?  We do not collect any identifiable information - Behavidence was designed with total privacy in mind. We take safety and security very seriously and operate under the AAED principle.  Abstraction Data is never stored as a complete set of behavioral data. Anonymization The data is completely anonymized and never stored together with any personal information. Even Behavidence employees can not access any of your personal information (e.g: Name, Address, etc). Encryption All the data collected is fully encrypted from the onset. Distribution We distribute the data to top-of-the-line servers in different locations to make sure that even in the worst-case scenario, no one will be able to use the data or identify any personal information. |
| Better- Rewards for Health | Information about confidentiality and handling of data (including any sharing with third parties) We do not sell any of your Personal Information or Personal Health Data to any third parties. Your information is encrypted and not shared unless you specify otherwise.  Your information is stored on MedStack’s HIPAA compliant server instances and you are responsible for managing your encryption and decryption keys.  If there are any specific clinical studies or future research opportunities a separate informed consent will be collected after you have been provided with more details.  Bowhead may improve its Services by analyzing non identifiable aggregated data.  From Website: Privacy Protection Blockchain technology ensures the confidentiality of your health data by encrypting it and distributing it across a decentralised network. |
| Chemo Brain Cognitive Research | We implement security safeguards designed to protect your data, such as HTTPS. We regularly monitor our systems for possible vulnerabilities and attacks. However, we cannot warrant the security of any information that you send us. There is no guarantee that data may not be accessed, disclosed, altered, or destroyed by breach of any of our physical, technical, or managerial safeguards. |
| Depression Cognitive Research | We implement security safeguards designed to protect your data, such as HTTPS. We regularly monitor our systems for possible vulnerabilities and attacks. However, we cannot warrant the security of any information that you send us. There is no guarantee that data may not be accessed, disclosed, altered, or destroyed by breach of any of our physical, technical, or managerial safeguards. |
| DNA Fit | While we cannot guarantee that unauthorised access, disclosure, misuse or loss of Information will never occur, Prenetics is certified to ISO/IEC 27001:2013 Information Security Management System Standard and frequently reviews and implements physical, technical, and administrative measures to prevent information security incidents and to maintain the confidentiality, integrity and availability of information.  All connections to Our Site and our mobile applications are encrypted using Secure Socket Layer (SSL) technology and internal systems protected with anti-virus software.  Only authorised personnel of Prenetics and contracted third parties have access to Information that is necessary for them to perform their jobs or services.   You must keep your account credentials secure and not share them with anyone. Your password for your account will be used only for online login. We will not ask for your password under any other circumstances. Inform Prenetics immediately of any unauthorised use of your account. Should you wish to reset or change your password, you can do so by clicking on the relevant links on Our Site.  Sharing Self-Reported Information through surveys, or other features on Our Site, is voluntary and done at your sole risk. Prenetics cannot take responsibility for Information that you release or that you request us to release publicly.  In the event of a security incident, Prenetics EMEA's internal procedures and those prescribed by the GDPR and DPA 2018 will be followed. You will be notified of any material impacts or direct consequences to you as a User without undue delay  From Website: What do you do with my data?  Our DNA is what makes us who we are, so we put in place a strict data protection policy, so you know your data is secure. We only test for the genes we need, and destroy your sample once the lab has completed the analysis. Your results are stored under an anonymised ID to create your reports.  We adhere to the UK Data Protection Act, we’re GDPR compliant and are proud to be the first consumer genetics company to be awarded ISO27001 certification – the highest international standard for data security and management. Customer consent should be mandatory before testing...  Customers should be required to provide their formal consent to genetic testing and sign a declaration confirming their understanding of how their data will be used. Demonstrate strict laboratory & data protection standards...  DTC genetics companies should disclose the location of the laboratory used to analyse samples to enable a customer to make an informed decision about the security of their DNA sample and the quality of the laboratory analysis. Commercialization Be commercially transparent...  If DTC genetics companies recommend or promote any additional products or services to their customers, such as nutritional supplements, they should clearly state if they have commercial relationships with third party suppliers. |
| Dyscalculia Cognitive Research | We implement security safeguards designed to protect your data, such as HTTPS. We regularly monitor our systems for possible vulnerabilities and attacks. However, we cannot warrant the security of any information that you send us. There is no guarantee that data may not be accessed, disclosed, altered, or destroyed by breach of any of our physical, technical, or managerial safeguards. |
| Dyslexia Cognitive Research | We implement security safeguards designed to protect your data, such as HTTPS. We regularly monitor our systems for possible vulnerabilities and attacks. However, we cannot warrant the security of any information that you send us. There is no guarantee that data may not be accessed, disclosed, altered, or destroyed by breach of any of our physical, technical, or managerial safeguards. |
| Fibromyalgia - Research | We implement security safeguards designed to protect your data, such as HTTPS. We regularly monitor our systems for possible vulnerabilities and attacks. However, we cannot warrant the security of any information that you send us. There is no guarantee that data may not be accessed, disclosed, altered, or destroyed by breach of any of our physical, technical, or managerial safeguards. |
| Google Fit | Health Research Applications and Web Services must comply with the Google Fit User Data and Developer Policy’s Secure Data Handling section.  Health Research Applications and Web Services are also recommended to follow best practices recommended by the U.S. Department of Health and Human Services, the U.S. Food and Drug Administration regulations, or ICH Good Clinical Practice Guidelines, like applying for a Certificate of Confidentiality from the National Institutes of Health, which may protect data from compelled disclosure to third parties. |
| Happiness Project- Play Games for Science | Is it anonymous? Yes. The data collection is anonymous and furthermore we won't use your information at any point to try to identify you. We don't ask for your name or for any other personal details, and we don't need your phone number to send notifications to your phone. We will never sell your data to any third party. We may make anonymous data available for further research by other parties such as academic researchers at other institutions.   Is it secure? The data is not encrypted before it is submitted over the internet. However, no personal information is used by the app or sent to us.   From Website Is my information safe? Your privacy is very important to us. Your data is completely anonymized, which means we do not know who you are and we cannot find out. The app won’t ask for your name, email, phone number, or any other information that could be used to identify you.  We will never sell your data to any third party. We may make your anonymous data available for further research by other parties such as academic researchers.  Taking part is completely voluntary. You can withdraw completely from the experiment at any time in the Settings menu..   For details on privacy, please see more information in our Terms & Conditions. |
| Healthy Minds Program | We limit the release of information to that described in this Privacy Policy, and we require privacy protections in our business relationships. |
| Hevy Gym Log Workout | N/A |
| Huawei Health | Your fitness and health data, which includes sensitive information about you, shall be uploaded to Huawei Health cloud only after you give your explicit consent in the app. You can go to Me > Privacy management in the app at any time to erase the data from the cloud and/or cease further collection and processing. We will not process your fitness and health data for any other purposes than storing it in the cloud and making it available for viewing and analysis from your mobile device, and for providing additional Huawei Health features you have explicitly consented to. To help you analyze your data over time, we will calculate e.g. different trends from the data you have synced to cloud and visualize those trends in the app. |
