# Supplementary material for: Mapping the Apps: Ethical and Legal Issues with Crowdsourced Smartphone Data using mHealth Applications
Source: Asian Bioeth Rev. 2024 Jun 18;16(3):437–70. doi: 10.1007/s41649-024-00296-3 (PMC11250705; doi:10.1007/s41649-024-00296-3)
Supplement: Supplementary file 6 — (DOCX 27.3 kb) [file 41649_2024_296_MOESM6_ESM.docx]

| Appendix 5: Return of Findings | |
| --- | --- |
| App | Return of Findings |
| 23andMe - DNA Testing | On an opt-in/opt-out basis |
| Ada - Check your Health | Right to be informed: As a data subject, you have a right to obtain access and information under the conditions provided in Article 15 GDPR. This means in particular that you have the right to obtain confirmation from us as to whether we are processing your personal data or not. If so, you also have the right to obtain access to the personal data and the information listed in Article 15(1) GDPR. This includes information regarding the purposes of the processing, the categories of personal data that are being processed, and the recipients or categories of recipients to whom the personal data have been or will be disclosed. |
| Ancestry: Family History & DNA | Right of Access/To Know – You may have a right to request access to your Personal Information and to be provided with a copy of certain information including the categories of your Personal Information we collect and disclose. To request a copy, follow these step-by-step instructions. If you want a copy of your DNA Data, follow these step-by-step instructions. If you want a copy of your family trees, follow these step-by-step instructions. |
| Apple Research | You may decide not to participate or you may leave any Study at any time. If you decide to withdraw, we may not delete the information that we have already collected and we may continue to use it, but we will stop receiving any new Study Data about you and will stop contacting you about the Study except in the case of serious, actionable medical need. |
| CovidWatcher | You may or may not personally benefit from this study. Your participation may help us gain valuable data to learn more about the coronavirus pandemic. |
| DNA ID, Inc. | Building a marketplace, our main problem is 2-sided. For the contributor, we are hoping to put transparency, and ownership of healthcare data back in their hands. For the researcher, we are tackling the time, cost, and access problems it takes for drug development of life saving treatments to come to market. |
| DnaNudge | You can contact our customer care team to request access to, edit or delete any personal information you have provided to us. We cannot guarantee we will be able to grant a request to change information, for example, if we believe granting such a request would violate the law or cause the information to be incorrect. It may not be possible to retrieve, remove or correct data from any database where the data had been de-identified and/or aggregated. |
| FLARe Research | N/A |
| Gene Doe | If within the EU: You may exercise Your rights of access, rectification, cancellation and opposition by contacting Us. Please note that we may ask You to verify Your identity before responding to such requests. If You make a request, We will try our best to respond to You as soon as possible. |
| GenePlanet | Your right to data portability  If you wish to receive your Personal Data processed by GenePlanet, we will provide it to you (or another individual or legal entity of your choosing) in a structured, machine-readable format if:  the processing is based on Consent or contract;  the processing is carried out by automated means.  Your right to object to processing  You have the right to object to the processing of your Personal Data, at any time, by sending a written request.  Your right to access your Personal Data  You may submit a request to GenePlanet to access your Personal Data at any time (if your Personal Data is processed) or your Personal Information and information about Personal Data processing (i.e., purposes of processing, types of Personal Data, retention periods, or the defining criteria of those periods, your rights, the source of the Personal Data, and other information required that is provided by Articles 14 and 15 of the GDPR). |
| Mass Science | Mass Science would like to make sure you are fully aware of all of your data protection rights. Every user is entitled to the following:  The right to access – You have the right to request Mass Science for copies of your personal data.  The right to rectification – You have the right to request that Mass Science correct any information you believe is inaccurate. You also have the right to request Mass Science to complete the information you believe is incomplete.  The right to erasure – You have the right to request that Mass Science erase your personal data, under certain conditions.  The right to restrict processing – You have the right to request that Mass Science restrict the processing of your personal data, under certain conditions.  The right to object to processing – You have the right to object to Mass Science’s processing of your personal data, under certain conditions.  The right to data portability – You have the right to request that Mass Science transfer the data that we have collected to another organization, or directly to you, under certain conditions. |
| My Toolbox Genomics | Upon your written request, Toolbox will remove any Genetic Information and Self-Reported Information provided by you to Toolbox and will cease using such Personal Information, subject to the terms of this Privacy Policy and the Toolbox Terms of Service. You may request access to and removal of such Personal Information and other Personal Information as collected by Toolbox by sending a written request to Toolbox.  Toolbox may not be able to completely remove all of Personal Information about a particular user from its systems. For example, Toolbox may retain Personal Information about a user for legitimate business purposes, if it may be necessary to prevent fraud or future abuse, for Account recovery purposes, if required by law, or as retained in Toolbox’s data backup systems or cached or archived pages. All retained Personal Information will continue to be subject to the terms of the Privacy Policy to which the User has previously agreed. Notwithstanding the foregoing, you always have the right to access Personal Information about you and to obtain from Toolbox confirmation as to what, how, and why Personal Information is being processed by Toolbox. |
| MyGeneRank | You have the right to view and verify Personal Information we collect about you. If you wish to access, rectify, delete or block any of the Personal Information, you may contact us at Generank@scripps.edu  Please note that while any changes you make will be reflected in active user databases within a reasonable period of time, we may maintain a copy of all information you submit for backups, archiving, prevention of fraud and abuse, analytics, satisfaction of legal obligations, or where we otherwise reasonably believe that we have a legitimate reason to do so, as permitted by applicable law.  You can withdraw from MyGeneRank studies at any time. Please note that if you withdraw from a study, we will stop collecting new data from you, but the coded study data that you have already provided will not be destroyed or deleted. |
| OH Data Port | N/A |
| Pattern Health | N/A |
| Project Serotonin | N/A |
| StuffThatWorks | If the law applicable to you grants you such rights, you may ask to access, correct, or delete your Personal Information that is stored in our systems. You may also ask for our confirmation as to whether or not we process your Personal Information. Under certain circumstances, you may have the right to restrict processing and/or object to the processing of your Personal Information. Subject to the limitations in law, you may request that we update, correct, or delete inaccurate or outdated information. You may also request that we suspend the use of any Personal Information whose accuracy you contest while we verify the status of that data. Subject to the limitations in law, you may also be entitled to obtain the Personal Information you directly provided us (excluding Information we obtained from other sources) in a structured, commonly used, and machine-readable format and may have the right to transmit such Information to another party. |
| Urban Mind | The results will be published in scientific journals and presented on our web site dedicated to the project (www.urbanmind.info) and social media. |
| Withings Health mate | You may exercise the following rights independently or with our assistance.  a. Right of Access. ou can access the Personal Data about you processed, collected or stored by WITHINGS. [...]  b. Right of rectification. If you find that the data about you is inaccurate, you have the right to request its correction. Some personal data can be changed directly from your Withings App account.  c. Right of Limitation and Right to Object. If you find that any data about you is inaccurate, you may ask us to stop processing that data until the situation is corrected. You may also ask Us to stop processing Data relating to you.  d. Right to Erasure. You may request the deletion of Personal Data relating to you. We will assist you in deleting Personal Data your account or Customer Support.  e. Right to Portability. You may request that we send you the Personal Data relating to you so that you can share it with another company. |
| ActiveDay - Activity Study | N/A |
| ADHD - Cognitive Research | We give you account settings and tools to access and control your personal data, as described below. If you live in certain jurisdictions, you may have legal rights with respect to your information, which your account settings and tools allow you to exercise, as outlined below.  Accessing and Exporting Data  By logging into your account, you can access much of your personal information, including your cognitive activity statistics. Using your account settings, you can also download information in a commonly used file format, including data about your activities and results.  Editing and Deleting Data  By logging into your account and using your account settings, you can change and delete your personal information.  If you choose to delete your account, please note that all data, scores, and information will be permanently deleted, except as noted below.  We retain your personal data even after you have closed your account if reasonably necessary to comply with our legal obligations (including law enforcement requests), meet regulatory requirements, resolve disputes, maintain security, prevent fraud and abuse, enforce our User Agreement, or fulfill your request to “unsubscribe” from further messages from us. We will retain de-personalized information after your account has been closed.  Information you have shared with others (e.g. with a parent, educator, health professional, researcher, or some of our partners) will remain visible after you closed your account or deleted the information from your own profile or mailbox, and we do not control data that other users copied out of our Services. Community features content (e.g. challenges) associated with closed accounts will show an anonymous user as the source.  Note that while most of your information will be deleted within 30 days, it may take up to 90 days to delete all of your information, like data stored in our backup systems.  Objecting to Data Use  We give you account settings and tools to control our data use. For example, through your privacy settings, you can limit how your information is visible to other users of the Services; using your notification settings, you can limit the notifications you receive from us; and under your account settings, you can revoke the access of third-party applications that you previously connected to your CogniFit account. |
| Andaman7 Private Health Record | You have a right of access, a right to be forgotten, a right of rectification, a right to limit processing, a right to portability, a right of opposition.  From Website:  Patients need their health data to get control over their own health. They need access to information and easy sharing of it for a better experience and quality of care. We made Andaman7 highly accessible by making it free for patients. We made it focused on easy, transparent and secure data collection and sharing. You can enrich your health record with data coming from your hospital, laboratory, doctors, ... and then share your health data with whoever you want. We made it the “all in one place” platform: one place to store all your health data, one place to collaborate with health professionals and organizations for better care. |
| Atlas Health | You have the right at any time:  To access and receive a copy of the Personal Data we hold about you. You can request to obtain a copy of your Personal Data in a commonly used electronic format so that you can manage and move it. Please note that we may ask you to verify your identity before responding to such requests.  To rectify any Personal Data held about you that is inaccurate  You have the right at any time to request that:  we remove your sample at any time by sending an e-mail to hello@atlasbiomed.co.uk with the word ‘Withdraw’ in the email title  we delete your Personal Data, health information and individual level genetic data by sending an email to hello@atlasbiomed.co.uk with the word ‘Forget me’ in the email title.  Please note that:  - if you request the removal or deletion of any data before the testing services have been completed, this may affect our ability to provide your results to you; and  - there may be certain information that we are required by law to retain for a definite period, in which case we will only be able to delete the information once that period has expired.  From Website:  Where can I get my results?  Your microbiome test results and raw data will be available in your online personal account at https://atlasbiomed.co.uk/account.  Your DNA test results and raw DNA data will be available in your online personal account.  Can I get the raw data for my DNA test results?  Yes, you can download the raw data that we use to interpret your results from your personal account. To do so, just click on your profile at the top right-hand corner of the page and select ‘Raw Data’. |
| Behavidence Research App | You have certain rights to access, update or delete information, obtain a copy of your information, and object or restrict certain data processing activities.  If you are in the EU EU/EEA you have the following rights under the GDPR:  Right to Access your personal data that we process and receive a copy of it.  Right to Rectify inaccurate personal data we have concerning you and to have incomplete personal data completed.  Right to Data Portability, that is, to receive the personal data that you provided to us, in a structured, commonly used and machine-readable format. You have the right to transmit this data to another service provider. Where technically feasible, you have the right that your personal data be transmitted directly from us to the service provider you designate.  Right to Object, based on your particular situation, to using your personal data on the basis of our legitimate interest. However, we may override the objection if we demonstrate compelling legitimate grounds, or for the establishment, exercise of defense of legal claims. You may also object at any time to the use of your personal data for direct marketing purposes.  Right to Restrict the processing your personal data (except for storing it) if you contest the accuracy of your personal data, for a period enabling us to verify its accuracy; if you believe that the processing is unlawful and you oppose the erasure of the personal data and request instead to restrict its use; if we no longer need the personal data for the purposes outlined in this Privacy Policy, but you require them to establish, exercise or defense relating to legal claims, or if you object to processing, pending the verification whether our legitimate grounds for processing override yours.  Right to be Forgotten. Under certain circumstances, such as when you object to us processing your data and we have no compelling legitimate grounds to override your objection, you have the right to ask us to erase your personal data. However, we may still process your personal data if it is necessary to comply with a legal obligation we are subject to under laws in EU Member States or for the establishment, exercise or defense of legal claims.  .  We reserve the right to ask for reasonable evidence to verify your identity before we provide you with information. Where we are not able to provide you the information that you have asked for, we will explain the reason for this. |
| Better - Rewards for Health | N/A |
| Chemo Brain Cognitive Research | We give you account settings and tools to access and control your personal data, as described below. If you live in certain jurisdictions, you may have legal rights with respect to your information, which your account settings and tools allow you to exercise, as outlined below.  Accessing and Exporting Data  By logging into your account, you can access much of your personal information, including your cognitive activity statistics. Using your account settings, you can also download information in a commonly used file format, including data about your activities and results.  Editing and Deleting Data  By logging into your account and using your account settings, you can change and delete your personal information.  If you choose to delete your account, please note that all data, scores, and information will be permanently deleted, except as noted below.  We retain your personal data even after you have closed your account if reasonably necessary to comply with our legal obligations (including law enforcement requests), meet regulatory requirements, resolve disputes, maintain security, prevent fraud and abuse, enforce our User Agreement, or fulfill your request to “unsubscribe” from further messages from us. We will retain de-personalized information after your account has been closed.  Information you have shared with others (e.g. with a parent, educator, health professional, researcher, or some of our partners) will remain visible after you closed your account or deleted the information from your own profile or mailbox, and we do not control data that other users copied out of our Services. Community features content (e.g. challenges) associated with closed accounts will show an anonymous user as the source.  Note that while most of your information will be deleted within 30 days, it may take up to 90 days to delete all of your information, like data stored in our backup systems.  Objecting to Data Use  We give you account settings and tools to control our data use. For example, through your privacy settings, you can limit how your information is visible to other users of the Services; using your notification settings, you can limit the notifications you receive from us; and under your account settings, you can revoke the access of third-party applications that you previously connected to your CogniFit account. |
| Depression Cognitive Research | We give you account settings and tools to access and control your personal data, as described below. If you live in certain jurisdictions, you may have legal rights with respect to your information, which your account settings and tools allow you to exercise, as outlined below.  Accessing and Exporting Data  By logging into your account, you can access much of your personal information, including your cognitive activity statistics. Using your account settings, you can also download information in a commonly used file format, including data about your activities and results.  Editing and Deleting Data  By logging into your account and using your account settings, you can change and delete your personal information.  If you choose to delete your account, please note that all data, scores, and information will be permanently deleted, except as noted below.  We retain your personal data even after you have closed your account if reasonably necessary to comply with our legal obligations (including law enforcement requests), meet regulatory requirements, resolve disputes, maintain security, prevent fraud and abuse, enforce our User Agreement, or fulfill your request to “unsubscribe” from further messages from us. We will retain de-personalized information after your account has been closed.  Information you have shared with others (e.g. with a parent, educator, health professional, researcher, or some of our partners) will remain visible after you closed your account or deleted the information from your own profile or mailbox, and we do not control data that other users copied out of our Services. Community features content (e.g. challenges) associated with closed accounts will show an anonymous user as the source.  Note that while most of your information will be deleted within 30 days, it may take up to 90 days to delete all of your information, like data stored in our backup systems.  Objecting to Data Use  We give you account settings and tools to control our data use. For example, through your privacy settings, you can limit how your information is visible to other users of the Services; using your notification settings, you can limit the notifications you receive from us; and under your account settings, you can revoke the access of third-party applications that you previously connected to your CogniFit account. |
| DNA Fit | If you have questions regarding how Prenetics handles your Information, or to request access or deletion of your Information held by Prenetics, please email our Data Protection Officer at compliance@dnafit.com. |
| Dyscalculia Cognitive Research | We give you account settings and tools to access and control your personal data, as described below. If you live in certain jurisdictions, you may have legal rights with respect to your information, which your account settings and tools allow you to exercise, as outlined below.  Accessing and Exporting Data  By logging into your account, you can access much of your personal information, including your cognitive activity statistics. Using your account settings, you can also download information in a commonly used file format, including data about your activities and results.  Editing and Deleting Data  By logging into your account and using your account settings, you can change and delete your personal information.  If you choose to delete your account, please note that all data, scores, and information will be permanently deleted, except as noted below.  We retain your personal data even after you have closed your account if reasonably necessary to comply with our legal obligations (including law enforcement requests), meet regulatory requirements, resolve disputes, maintain security, prevent fraud and abuse, enforce our User Agreement, or fulfill your request to “unsubscribe” from further messages from us. We will retain de-personalized information after your account has been closed.  Information you have shared with others (e.g. with a parent, educator, health professional, researcher, or some of our partners) will remain visible after you closed your account or deleted the information from your own profile or mailbox, and we do not control data that other users copied out of our Services. Community features content (e.g. challenges) associated with closed accounts will show an anonymous user as the source.  Note that while most of your information will be deleted within 30 days, it may take up to 90 days to delete all of your information, like data stored in our backup systems.  Objecting to Data Use  We give you account settings and tools to control our data use. For example, through your privacy settings, you can limit how your information is visible to other users of the Services; using your notification settings, you can limit the notifications you receive from us; and under your account settings, you can revoke the access of third-party applications that you previously connected to your CogniFit account.  From Website:  After completing the dyscalculia assessment, you will automatically receive a detailed report, where you will see the risk index of having dyscalculia (low-medium-high), warning signs, cognitive profile, results analysis, recommendations, and guidelines. The results offer valuable information, and makes it possible to identify support strategies or recommendations to see a specialist for more in-depth testing. |
| Dyslexia Cognitive Research | We give you account settings and tools to access and control your personal data, as described below. If you live in certain jurisdictions, you may have legal rights with respect to your information, which your account settings and tools allow you to exercise, as outlined below.  Accessing and Exporting Data  By logging into your account, you can access much of your personal information, including your cognitive activity statistics. Using your account settings, you can also download information in a commonly used file format, including data about your activities and results.  Editing and Deleting Data  By logging into your account and using your account settings, you can change and delete your personal information.  If you choose to delete your account, please note that all data, scores, and information will be permanently deleted, except as noted below.  We retain your personal data even after you have closed your account if reasonably necessary to comply with our legal obligations (including law enforcement requests), meet regulatory requirements, resolve disputes, maintain security, prevent fraud and abuse, enforce our User Agreement, or fulfill your request to “unsubscribe” from further messages from us. We will retain de-personalized information after your account has been closed.  Information you have shared with others (e.g. with a parent, educator, health professional, researcher, or some of our partners) will remain visible after you closed your account or deleted the information from your own profile or mailbox, and we do not control data that other users copied out of our Services. Community features content (e.g. challenges) associated with closed accounts will show an anonymous user as the source.  Note that while most of your information will be deleted within 30 days, it may take up to 90 days to delete all of your information, like data stored in our backup systems.  Objecting to Data Use  We give you account settings and tools to control our data use. For example, through your privacy settings, you can limit how your information is visible to other users of the Services; using your notification settings, you can limit the notifications you receive from us; and under your account settings, you can revoke the access of third-party applications that you previously connected to your CogniFit account.  From Website:  After completing the dyslexia test, you will receive a detailed report, where you will see the user's risk index for dyslexia (low-medium-high), the warning signs and symptoms, cognitive profile, analysis of results, and recommendations. These results offer valuable information to identify support strategies or to bring to a specialist who can make a more precise diagnosis. |
| Fibromyalgia - Research | We give you account settings and tools to access and control your personal data, as described below. If you live in certain jurisdictions, you may have legal rights with respect to your information, which your account settings and tools allow you to exercise, as outlined below.  Accessing and Exporting Data  By logging into your account, you can access much of your personal information, including your cognitive activity statistics. Using your account settings, you can also download information in a commonly used file format, including data about your activities and results.  Editing and Deleting Data  By logging into your account and using your account settings, you can change and delete your personal information.  If you choose to delete your account, please note that all data, scores, and information will be permanently deleted, except as noted below.  We retain your personal data even after you have closed your account if reasonably necessary to comply with our legal obligations (including law enforcement requests), meet regulatory requirements, resolve disputes, maintain security, prevent fraud and abuse, enforce our User Agreement, or fulfill your request to “unsubscribe” from further messages from us. We will retain de-personalized information after your account has been closed.  Information you have shared with others (e.g. with a parent, educator, health professional, researcher, or some of our partners) will remain visible after you closed your account or deleted the information from your own profile or mailbox, and we do not control data that other users copied out of our Services. Community features content (e.g. challenges) associated with closed accounts will show an anonymous user as the source.  Note that while most of your information will be deleted within 30 days, it may take up to 90 days to delete all of your information, like data stored in our backup systems.  Objecting to Data Use  We give you account settings and tools to control our data use. For example, through your privacy settings, you can limit how your information is visible to other users of the Services; using your notification settings, you can limit the notifications you receive from us; and under your account settings, you can revoke the access of third-party applications that you previously connected to your CogniFit account.  From Website  At the end of the fibromyalgia test, CogniFit generates a fully detailed results report, which shows the risk index for this disease (low- medium-high), warning signs and symptoms, cognitive profile, analysis of results, and recommendations. The results provide valuable information to identify support strategies. |
| Google Fit | What will you do with this data? We'll use it for our academic research, and to create summary data that all app users can see. We'll apply statistical methods to the combined responses from everyone taking part. We’ll publish the results of our research in scientific journals. The papers will be available to read online – go to the news page on our website to see details of recent studies. Based on the data we receive from you, you may also be invited to take part in further research by an anonymous invitation sent through the app. |
| Happiness Project - Play Games for Science | What will you do with this data? We'll use it for our academic research, and to create summary data that all app users can see. We'll apply statistical methods to the combined responses from everyone taking part. We’ll publish the results of our research in scientific journals. The papers will be available to read online – go to the news page on our website to see details of recent studies. Based on the data we receive from you, you may also be invited to take part in further research by an anonymous invitation sent through the app. |
| Healthy Minds Program | Control Over and Access to Your Information We enable you to have control over the accuracy of your personal information. You can delete or change your personal information with us at any time by logging into your account or contacting us via a method described under “Contact Us” below.  More specific rights for EU, Switzerland, Mexico |
| Hevy Gym Log Workout | In particular, Users have the right to do the following, to the extent permitted by law:  -Withdraw their consent at any time. Users have the right to withdraw consent where they have previously given their consent to the processing of their Personal Data.  Object to processing of their Data. Users have the right to object to the processing of their Data if the processing is carried out on a legal basis other than consent. Further details are provided in the dedicated section below.  -Access their Data. Users have the right to learn if Data is being processed by the Owner, obtain disclosure regarding certain aspects of the processing and obtain a copy of the Data undergoing processing.  Verify and seek rectification. Users have the right to verify the accuracy of their Data and ask for it to be updated or corrected.  -Restrict the processing of their Data. Users have the right to restrict the processing of their Data. In this case, the -Owner will not process their Data for any purpose other than storing it.  -Have their Personal Data deleted or otherwise removed. -Users have the right to obtain the erasure of their Data from the Owner.  -Receive their Data and have it transferred to another controller. Users have the right to receive their Data in a structured, commonly used and machine readable format and, if technically feasible, to have it transmitted to another controller without any hindrance.  -Lodge a complaint. Users have the right to bring a claim before their competent data protection authority. |
| Huawei Health | You have the following rights and options:  5.1 Access your data  You can request information and a copy of your personal data that we have collected and stored in relation to Huawei Health by going to S[...] then clicking or touching Request Your Data.  For additional access requests, please contact us.  5.2 Rectify your data [..].  5.3 Port your data  You can port your personal data that you have provided to us in relation to Huawei Health in a commonly-used machine-readable format by going to [...].  5.4 Erase your data [...]  5.5 Withdraw your consent [...]  5.6 Object to processing  You can object to the processing of your data for analytics and improvement purposes by going to Me > Privacy management and disabling Analysis and improvement.  [...]  5.7 Restrict processing |
| InsideTracker | Your Rights  -Request a Copy of your Personal Information. You have the right to request a copy of any Personal Information that we hold about you. If you would like a copy of your Personal Information, please contact us using the contact information below. We may request proof of your identity before sharing such information. If you discover that the information we hold about you is incorrect or out of date, you may ask us to correct that information by contacting us using the contact information below.  -Cease Processing or Delete Personal Information. You may ask us to stop processing, or delete, the personally identifiable data we hold about you in certain circumstances. It may not be possible for us to stop processing or delete all of the information we hold about you where we are fulfilling a transaction or have a legal basis to retain the information, however please contact us to discuss how we can assist you with your request.  -Withdraw Consent. When we process your information on the basis that you have consented to such processing, you have the right to withdraw your consent, or ask us to stop or restrict processing the Personal Information we have about you, at any time by contacting us using the contact information below.  -Portability. You may also ask us to transfer your Personal Information to a third party in certain circumstances. If you would like any further information about your rights or how to exercise them, please contact us using the contact information below.  -Complaints. If you are in the European Union, you have the right to make a complaint at any time to the relevant data protection authority in your country.  Retention. We will retain your information for as long as needed to fulfill your requests, provide you services, comply with our legal obligations, resolve disputes, and enforce our agreements.  -Unsubscribe. If you receive marketing emails from us, you can unsubscribe to our emails by clicking "unsubscribe" within each email. You may not opt-out of service-related communications, which are not promotional in nature. |
| Insomnia - Cognitive Research | We give you account settings and tools to access and control your personal data, as described below. If you live in certain jurisdictions, you may have legal rights with respect to your information, which your account settings and tools allow you to exercise, as outlined below.  Accessing and Exporting Data  By logging into your account, you can access much of your personal information, including your cognitive activity statistics. Using your account settings, you can also download information in a commonly used file format, including data about your activities and results.  Editing and Deleting Data  By logging into your account and using your account settings, you can change and delete your personal information.  If you choose to delete your account, please note that all data, scores, and information will be permanently deleted, except as noted below.  We retain your personal data even after you have closed your account if reasonably necessary to comply with our legal obligations (including law enforcement requests), meet regulatory requirements, resolve disputes, maintain security, prevent fraud and abuse, enforce our User Agreement, or fulfill your request to “unsubscribe” from further messages from us. We will retain de-personalized information after your account has been closed.  Information you have shared with others (e.g. with a parent, educator, health professional, researcher, or some of our partners) will remain visible after you closed your account or deleted the information from your own profile or mailbox, and we do not control data that other users copied out of our Services. Community features content (e.g. challenges) associated with closed accounts will show an anonymous user as the source.  Note that while most of your information will be deleted within 30 days, it may take up to 90 days to delete all of your information, like data stored in our backup systems.  Objecting to Data Use  We give you account settings and tools to control our data use. For example, through your privacy settings, you can limit how your information is visible to other users of the Services; using your notification settings, you can limit the notifications you receive from us; and under your account settings, you can revoke the access of third-party applications that you previously connected to your CogniFit account.  From Website:  At the end of each training session, a summary is shown with the most relevant data. This helps to know what our strengths and weaknesses are, or if we have improved or worsened our score.  Not only does it provide information on the last session, but it also informs us about our general tendency and evolution. Even though we scored worse in the last session, our evolution can be positive and show progress. |
| Medisafe Pill & Med Reminder | Data protection laws in certain jurisdictions provide individuals with certain statutory rights with respect to their Personal Information.  For example, if you reside in the UK or the EU, you may have the right to request to: (a) receive confirmation as to whether or not Personal Information concerning you is being processed, and access your stored Personal Information, together with supplementary information; (b) receive a copy of Personal Information you directly volunteer to us in a structured, commonly used and machine-readable format; (c) request rectification of your Personal Information that is in our control; (d) request erasure of your Personal Information; (e) object to the processing of Personal Information by us; (f) request to restrict processing of your Personal Information by us; and (g) lodge a complaint with a supervisory authority if you believe your privacy rights have been prejudiced by us.  Data subjects residing in other jurisdictions may also be afforded with certain rights with respect to their personal data, as determined by such jurisdictions’ applicable laws. Such rights maybe similar or may differ from those set out above with respect to EU residents. |
| MyTherapy Pill Reminder | You have the right to request from us access to and rectification or erasure of personal data or restriction of processing concerning you or to object to processing as well as the right to data portability. We do not process your personal data for automated decision-making. You are free to withdraw any consent you have given at any time.  If you have any questions or wish to exercise your rights, please contact us e.g. at support@mytherapyapp.com. |
| NeuroPsy Research | After completing a study, the data will possibly be stored and published in a national or international data archive. These studies will thus follow the recommendations of the German Research Foundation (DFG) and the German Society for Psychology (DGPs) for quality assurance in research. |
| Parkinson’s Cognitive Research | We give you account settings and tools to access and control your personal data, as described below. If you live in certain jurisdictions, you may have legal rights with respect to your information, which your account settings and tools allow you to exercise, as outlined below.  Accessing and Exporting Data  By logging into your account, you can access much of your personal information, including your cognitive activity statistics. Using your account settings, you can also download information in a commonly used file format, including data about your activities and results.  Editing and Deleting Data  By logging into your account and using your account settings, you can change and delete your personal information.  If you choose to delete your account, please note that all data, scores, and information will be permanently deleted, except as noted below.  We retain your personal data even after you have closed your account if reasonably necessary to comply with our legal obligations (including law enforcement requests), meet regulatory requirements, resolve disputes, maintain security, prevent fraud and abuse, enforce our User Agreement, or fulfill your request to “unsubscribe” from further messages from us. We will retain de-personalized information after your account has been closed.  Information you have shared with others (e.g. with a parent, educator, health professional, researcher, or some of our partners) will remain visible after you closed your account or deleted the information from your own profile or mailbox, and we do not control data that other users copied out of our Services. Community features content (e.g. challenges) associated with closed accounts will show an anonymous user as the source.  Note that while most of your information will be deleted within 30 days, it may take up to 90 days to delete all of your information, like data stored in our backup systems.  Objecting to Data Use  We give you account settings and tools to control our data use. For example, through your privacy settings, you can limit how your information is visible to other users of the Services; using your notification settings, you can limit the notifications you receive from us; and under your account settings, you can revoke the access of third-party applications that you previously connected to your CogniFit account.  From Website:  After each training session, CogniFit provides access to updated data so that we can get quick and accurate feedback, seeing what are our strongest and weakest cognitive abilities.  The results of each session are stored in the user's profile, so you can check whether there have been changes from one session to another, whether the scores are improving and whether the patient's cognitive status is progressing positively. |
| Renpho Health | You may at any time review or change the data in your account or terminate your account by:  Logging into your account settings and updating your account; or  Contacting us using the contact information provided below in: “Contact Us.”  Upon your request to terminate your account, we will deactivate or delete your account and personal data from our active databases. However, some data may be retained in our files for permitted or required uses, such as to prevent fraud, troubleshoot problems, assist with any investigations, enforce our Terms of Use and/or comply with legal requirements.  You may also exercise your rights, subject to applicable laws, to request that we provide you with certain information on the personal data we have collected about you, or to correct, delete, or restrict our access to your personal data. Please note that we may retain certain data as required or permitted by applicable law. If you are a California resident or European or UK citizen, your specific rights are detailed, respectively, in the “California Privacy Rights” and “European Data Protection Rights” sections below. Under the laws of some regions and countries, we have the power to refuse your requests that are unreasonable or for which access is not required by local law. |
| Smart Omix by Sharecare | Right of access, restriction of processing, erasure. You may contact us to request information about the Personal Information we have collected from you, or to request that your Personal Information be deleted. We will do our best to honor your requests, subject to any legal, ethical and contractual obligations.  Your right to access or delete the Personal Information we hold about you is not absolute. There are instances where applicable law or regulatory requirements allow or require us to refrain from taking certain actions with respect to some or all of the Personal Information that we hold about you. In addition, some or all of the Personal Information may have been destroyed, erased or made anonymous. In the event we cannot provide you with access to your Personal Information, or we are unable to delete it, we will inform you of the reasons why, subject to any legal or regulatory restrictions  (right to correct information, withdraw consent, opt out, data portability, lodge a complaint, etc.) |
| Symptom & Mood Tracker | Once you have registered, you will benefit from the following functionality:  Access to platform to view any data that you choose to submit  Automatic backup to our secure servers. as well as the ability to restore and recover your account and it’s associated data on another device |
| Symptomate - Symptom Checker | To ensure that you have adequate control over your personal data transferred outside the European Economic Area, you will have the right to obtain a copy of your personal data transferred to third countries at any time.  You have the following rights under the GDPR:  The right to request access to your data and to receive a copy of your data: whenever possible, you can access, update or request deletion of your personal data;  The right to rectify (correct) your data: you have the right to have your information rectified if that information is inaccurate or incomplete;  The right to erasure: you have a right to erasure regarding data that are no longer required for the original purposes or that are processed unlawfully;  The right to data portability: you have the right to be provided with a copy of the information we have regarding you in a structured, machine-readable and commonly used format;  The right to object: you have the right to object to our processing of your personal data – upon your justified objection we will cease any further processing under Article 6(1)(f) of the GDPR;  The right to withdraw consent: you also have the right to withdraw your consent at any time where we relied on your voluntary consent to process your personal information;  The right to lodge a complaint with the competent supervisory authority - about our collection and use of your personal data. |
