# Supplementary material for: Mapping the Apps: Ethical and Legal Issues with Crowdsourced Smartphone Data using mHealth Applications
Source: Asian Bioeth Rev. 2024 Jun 18;16(3):437–70. doi: 10.1007/s41649-024-00296-3 (PMC11250705; doi:10.1007/s41649-024-00296-3)
Supplement: Supplementary file 7 — (DOCX 17.0 kb) [file 41649_2024_296_MOESM7_ESM.docx]

| Appendix 6: Algorithm Mentions in Privacy Policy | |
| --- | --- |
| App | Algorithm in Privacy Policy |
| 23andMe - DNA Testing | No |
| Ada - Check your Health | No |
| Ancestry: Family History & DNA | No |
| Apple Research | No |
| CovidWatcher | No |
| DNA ID, Inc. | N/A |
| DnaNudge | We use personal data for the following purposes: For research and development purposes (including machine learning) in order to improve or personalise the Service and to help us understand our customers and how our Service is used; To aggregate data to allow it to be used for statistical and research purposes |
| FLARe Research | N/A |
| Gene Doe | N/A |
| GenePlanet | Health Score and Food ID; An algorithm based on the results of other analyses and provided information (Habits questionnaire, Body measurements, and Blood test results). Lifestyle Insights and Food ID recommendations are based on the Health Score algorithm, Body measurements, Habits questionnaire, and Blood test results. |
| Mass Science | N/A |
| My Toolbox Genomics | Toolbox Genomics may use your de-identified and anonymized data to help improve our algorithm and reports. Your data will not be sold or shared to or with any 3rd parties/commercial interests. |
| MyGeneRank | Lifestyle Insights and Food ID recommendations are based on the Health Score algorithm, Body measurements, Habits questionnaire, and Blood test results; GenePlanet can process Personal Data for research purposes to gain new potential insights/findings in science   Your Unused Biological Sample and Personal Data will be stored in pseudonymised form until you request their destruction. In cases when they are used for research purposes, they will be anonymised. Data Processed |
| OH Data Port | No |
| Pattern Health | No |
| Project Serotonin | We use your information in accordance with this Privacy Policy for activities necessary for enabling the Service that includes analysis of data, generate and deliver Program recommendations, personalize your Supplements and improve our Program Services; These activities may include but not limited to: vii) perform Research Studies; viii) conducting data analysis to improve existing Program Services or develop new Services; and ix) improving our data analytics and algorithm engine that help us provide more precise and accurately personalized recommendations; We reserve the right to use Anonymous Data and aggregated and other de-identified information for any purpose and disclose Anonymous Data to third parties at our sole discretion, including for research purposes; |
| StuffThatWorks | No |
| Urban Mind | No |
| Withings Health mate | We collect your consent to process personal data for: participating in our research programs.   Product and Service Improvement (including algorithm performance improvement and statistics) |
| ActiveDay - Activity Study | No |
| ADHD - Cognitive Research | No |
| Andaman7 Private Health Record | No |
| Atlas Health | No |
| Behavidence Research App | No |
| Better- Rewards for Health | No |
| Chemo Brain Cognitive Research | No |
| Depression Cognitive Research | No |
| DNA Fit | No |
| Dyscalculia Cognitive Research | No |
| Dyslexia Cognitive Research | No |
| Fibromyalgia - Research | No |
| Google Fit | No |
| Happiness Project- Play Games for Science | No |
| Healthy Minds Program | No |
| Hevy Gym Log Workout | No |
| Huawei Health | No |
| InsideTracker | InsideTracker analyzes your DNA data using an algorithm that determines your genetic potential for certain traits. InsideTracker may use your aggregated, de-identified genetic data for research and development to improve future products. For research that we hope to publish in scientific publications, we will request separate permission through a Research Consent document to use your de-identified Genetic Information. Any Research Consent is optional and voluntary. You will not be required to agree to a Research Consent document in order to use the Platform or Services. |
| Insomnia - Cognitive Research | No |
| Medisafe Pill & Med Reminder | No |
| MyTherapy Pill Reminder | No |
| NeuroPsy Research | No |
| Parkinson's Cognitive Research | No |
| Renpho Health | No |
| Smart Omix by Sharecare | No |
| Symptom & Mood Tracker | No |
| Symptomate - Symptom checker | Automated Decision Making When you are using Services through the Website and the App, we collect certain information regarding health conditions, as well as other information that may be helpful for correct provision of Services, e.g. regarding sex, age, individual risk factor, region of residence, or everyday behavior; Such information regarding health is not combined with information that makes it possible for us to identify the person regarded by the medical history. Such information is combined with a unique identifier in order to obtain insight into statistical data allowing us to improve our Services; You use the Application thanks to our intelligent algorithm, which carefully analyzes your answers given in the interview, so you can learn the possible causes of your symptoms. This analysis is done automatically, based on the information you provide and, as a result, the tool can suggest your potential health condition. This process is referred to as "profiling" and its use if it concerns personal data is regulated by law (Article 22 of GDPR). This analysis is the essence of the service and must be carried out for you to receive an interview result. In addition, we also analyze the way in which you use the Application - thanks to this we can improve the quality of our solution and make the medical device not only safer but also more friendly and effective. |
