# Supplementary material for: Mapping the Apps: Ethical and Legal Issues with Crowdsourced Smartphone Data using mHealth Applications
Source: Asian Bioeth Rev. 2024 Jun 18;16(3):437–70. doi: 10.1007/s41649-024-00296-3 (PMC11250705; doi:10.1007/s41649-024-00296-3)
Supplement: Supplementary file 8 — (DOCX 17.8 kb) [file 41649_2024_296_MOESM8_ESM.docx]

| Appendix 7: Algorithms or AI mentioned in App Descriptions | |
| --- | --- |
| App | Algorithm/AI mentions in App Description |
| 23andMe - DNA Testing | Yes: The 23andMe Ancestry Composition algorithm combines information about these patterns with the unique set of DNA alleles in your genome to estimate your genetic ancestry.  Our Ancestry Composition algorithm handles the challenge of admixture by breaking your chromosomes into short adjacent windows, like boxcars in a train. These windows are small enough that it is generally safe to assume that you inherited all the DNA in any given window from a single ancestor many generations back.  The Ancestry Composition algorithm calculates your ancestry by comparing your genome to the genomes of people whose ancestries we already know. |
| Ada - Check your Health | Yes: our medical AI simplifies healthcare journeys and helps people take care of themselves. |
| Ancestry: Family History & DNA | Yes: If a match is labeled incorrectly or is unassigned, you can change their label. From your list of DNA matches, click on their name > Edit Relationship > select the right parent label > Save. Our algorithm will improve over time—but in the meantime, you can change the label for any match. |
| Apple Research | No |
| CovidWatcher | No |
| DNA ID, Inc. | No |
| DnaNudge | Yes: Every time you scan a barcode, our algorithms look at thousands of ‘parameters’ to deliver a precise recommendation based on your DNA; |
| FLARe Research | No |
| Gene Doe | No |
| GenePlanet | Yes: Through the years of research, our scientists have gained invaluable expertise in bioinformatic analysis, algorithm development, and machine learning systems—the key to complex calculations and the automatisation of processes. |
| Mass Science | N/A |
| My Toolbox Genomics | No |
| MyGeneRank | We have updated the algorithm used to calculate a participant’s genetic risk for CAD within MyGeneRank to include these new discoveries. |
| OH Data Port | No |
| Pattern Health | No |
| Project Serotonin | No |
| StuffThatWorks | Data is analyzed: smart machine learning algorithms seek out which treatments work best |
| Urban Mind | No |
| Withings Health mate | Yes: "simple and smart app" |
| ActiveDay - Activity Study | No |
| ADHD - Cognitive Research | Cognitive Assessment for ADHD Patients (СAB-ADHD) uses patented algorithms and artificial intelligence (AI) which makes it possible to analyze thousands of variables and notify the user of a risk of ADD or ADHD which very satisfactory psychometric results |
| Andaman7 Private Health Record | Patients could request this risk evaluation, directly from within Andaman7, after sharing an anonymous copy of (only) the data needed by the algorithm. A report will be generated by Consonance and sent back to the patients, securely, directly into their health record. And the patients may, if desired, share it further with their doctor. |
| Atlas Health | No |
| Behavidence Research App | Yes: Discover your mental health similarity scores: our AI based algorithm gives you daily feedback on your mental well-being. based on how you interact with your mobile |
| Better- Rewards for Health | No |
| Chemo Brain Cognitive Research | The Cognitive Assessment for Chemo Fog Patients (CAB-CF) from CogniFit uses patented algorithms and artificial intelligence (AI) that makes it possible to analyze thousands of variables and highlight any risk of Mental Fog with very satisfactory psychometric results. |
| Depression Cognitive Research | The Cognitive Assessment for Depression Patients (CAB-DP) from CogniFit uses patented algorithms and artificial intelligence (AI) to analyze thousands of variables and inform you of any risk for depression, with very satisfactory psychometric results. |
| DNA Fit | Fitness insights include: DNAFit Peak Performance Algorithm® |
| Dyscalculia Cognitive Research | The Cognitive Assessment for Dyscalculia Patients (CAB-DC) from CogniFit uses patented algorithms and artificial intelligence (AI), which makes it possible to analyze thousands of variables and detect a possible risk of dyscalculia with very satisfactory psychometric results. |
| Dyslexia Cognitive Research | The Cognitive Assessment for Dyslexia Patients (CAB-DX) uses patented algorithms and artificial intelligence (AI), which makes it possible to analyze thousands of variable and notify users of a risk of dyslexia with very satisfactory psychometric results. |
| Fibromyalgia - Research | CogniFit's Cognitive Assessment for Fibromyalgia Patients (CAB-FB), uses patented algorithms and artificial intelligence (AI), which makes it possible to analyze thousands of variables and notify users whether there is a risk of fibromyalgia with very satisfactory psychometric results. |
| Google Fit | No |
| Happiness Project- Play Games for Science | No |
| Healthy Minds Program | No |
| Hevy Gym Log Workout | No |
| Huawei Health | No |
| InsideTracker | The algorithm that drives the InsideTracker platform is continuously refined, drawing on cutting-edge research and technological advances. It’s smart and ever-evolving, just like your body is.  But we’re more than machines. We build and grow our products using both innovative technology and the human brain power of our accomplished scientific team. |
| Insomnia - Cognitive Research | The Cognitive Assessment for Insomnia Patients (CAB-IN) from CogniFit uses patented algorithms and artificial intelligence (AI). |
| Medisafe Pill & Med Reminder | JITI powers Medisafe’s medication engagement platform as the predictive machine learning engine driving personalized patient support.  JITI technology increases activity, improves adherence, and extends treatment retention through personalized patient support.  Machine-learning optimized interventions determine which channel, and what content, is used for patient support. |
| MyTherapy Pill Reminder | No |
| NeuroPsy Research | No |
| Parkinson's Cognitive Research | CogniFit's Cognitive Assessment for Parkinson’s Disease Patients (CAB-PK), uses patented algorithms and artificial intelligence (AI), which makes it possible to analyze thousands of variables and notify users whether there is a risk of Parkinson's with very satisfactory psychometric results. |
| Renpho Health | The cloud-based App’s intelligent data analysis and tracking abilities makes it your perfect digital personal helper. It can even convert your data stored over time into charts and reports that can easily be shared by e-mail and multiple social media channels. |
| Smart Omix by Sharecare | No |
| Symptom & Mood Tracker | No |
| Symptomate - Symptom checker | With just a few clicks each day our intelligent health diary can help you to gain insights into which daily activities and health factors are positively and negatively affecting your mental and physical health; Sync health data automatically. Such as Sleep, Steps, and Heart Rate from GoogleFit; With feedback and feature requests from a research group of thousands of people with a wide variety of chronic diseases and both mental and physical health conditions |
